# Massive Transcriptional Perturbation in Subgroups of Diffuse Large B-Cell Lymphomas

**DOI:** 10.1371/journal.pone.0076287

**Published:** 2013-11-04

**Authors:** Maciej Rosolowski, Jürgen Läuter, Dmitriy Abramov, Hans G. Drexler, Michael Hummel, Wolfram Klapper, Roderick A.F. MacLeod, Shoji Pellissery, Friedemann Horn, Reiner Siebert, Markus Loeffler

**Affiliations:** 1 Institute of Medical Informatics, Statistics and Epidemiology (IMISE), University of Leipzig, Leipzig, Germany; 2 Otto von Guericke University Magdeburg, Magdeburg, Germany; 3 Department of Pathology, Hematopathology Section and Lymph Node Registry, University of Kiel, Kiel, Germany; 4 Department of Pathology, Russian Federal Research Center, Moscow, Russia; 5 Department of Human and Animal Cell Lines, DSMZ – German Collection of Microorganisms and Cell Cultures, Braunschweig, Germany; 6 Institute of Pathology, Campus Benjamin Franklin, Charité-Universitätsmedizin Berlin, Germany; 7 Institute of Human Genetics, University Hospital Schleswig-Holstein Campus Kiel/University of Kiel, Kiel, Germany; 8 Institute of Clinical Immunology, Medical Faculty, University of Leipzig, Leipzig, Germany; Queen's University Belfast, United Kingdom

## Abstract

Based on the assumption that molecular mechanisms involved in cancerogenesis are characterized by groups of coordinately expressed genes, we developed and validated a novel method for analyzing transcriptional data called Correlated Gene Set Analysis (CGSA). Using 50 extracted gene sets we identified three different profiles of tumors in a cohort of 364 Diffuse large B-cell (DLBCL) and related mature aggressive B-cell lymphomas other than Burkitt lymphoma. The first profile had high level of expression of genes related to proliferation whereas the second profile exhibited a stromal and immune response phenotype. These two profiles were characterized by a large scale gene activation affecting genes which were recently shown to be epigenetically regulated, and which were enriched in oxidative phosphorylation, energy metabolism and nucleoside biosynthesis. The third and novel profile showed only low global gene activation similar to that found in normal B cells but not cell lines. Our study indicates novel levels of complexity of DLBCL with low or high large scale gene activation related to metabolism and biosynthesis and, within the group of highly activated DLBCLs, differential behavior leading to either a proliferative or a stromal and immune response phenotype.

## Introduction

Diffuse large B-cell lymphoma (DLBCL) is a biologically and clinically heterogeneous disease [1], [2], [3], [4], [5], [6]. Patients whose tumors share similar histopathological characteristics differ with respect to underlying genetic changes, clinical outcome and response to specific therapies [1], [2], [4], [5]. Unlike in Burkitt lymphoma where *MYC* translocations occur almost always [4] and recent sequencing studies of our and other groups have shown the presence of highly recurrent mutations [7], [8], [9] no such highly prevalent genetic abnormalities have been found in DLBCL [10], [11], [12], [13]. Analyses of gene expression have led to identification of molecular subtypes of DLBCLs. These include the cell of origin signatures for activated B-cell-like diffuse large B-cell lymphomas (ABC) and the germinal center B-cell-like diffuse large B-cell lymphomas (GCB) [1], [2], the “consensus clusters” [3] referred to as “oxidative phosphorylation” (OxPhos), “B-cell receptor/proliferation” (BCR) and “host response” (HR), and the “pathway activation patterns” [6] (PAPs) denoted by PAP-1 to PAP-4, BL-PAP and “molecularly individual lymphomas” (mind-L).

The heterogeneity of DLBCL as that of several other types of cancer is believed to arise as a consequence of a number of aberrations causing different patterns of deregulation of cell signaling pathways [11], [14]. This view suggests that groups of co-expressed genes which are expected to be observed as a result of deregulation of signaling pathways, may carry most of the information about the heterogeneity of tumors. Here, we present and apply a novel biostatistical approach designed to derive sets of co-expressed genes. These gene sets can be used in subsequent analyses, e.g., tests for association with other phenotypes and in unsupervised analysis of the samples.

Until now, in most gene expression studies of DLBCLs, transcriptional differences related to the cell of origin or to the activation of specific pathways have been of primary biological interest. A feature of the approach introduced here is that it enables performing an analysis without any specific biological hypotheses in mind. This lack of bias is important since there might exist mechanisms of oncogenic gene deregulation, e.g., histone modifications which might play a role in lymphomagenesis or cancer in general across the presently known tumor subtypes.

We apply our method to an extensive data set of recently published 364 DLBCL and related mature aggressive B-cell lymphomas other than Burkitt lymphoma [15]. An unsupervised analysis with respect to the generated gene sets reveals three groups of samples, two of which are characterized by a massive transcriptional activation. We find that this activation is associated with genes which were recently shown to correlate with histone modifications [16]. Moreover, the upregulated genes are enriched in metabolic processes. These findings provide a basis for further functional investigations, in particular in light of recent discoveries related to epigenetic deregulation in lymphoid malignancies [10], [17], [18].

## Results

### Establishment and validation of the method on two BL/DLBCL data sets

We developed a novel method called Correlated Gene Set Analysis (CGSA) for unbiased and hypothesis-free analysis of large gene expression data sets. Bioinformatic details of the method and the discussion of its relationship to other approaches are given in Materials and Methods and in Text S1. In brief, the method performs a dimension reduction by extracting a small number of sets of correlated genes (CGSs). Each set contains a number of genes which are tightly and positively co-expressed with one “central” gene of the set. The extracted CGSs are non-overlapping and reflect the variety of expression patterns present in the analyzed samples. Each CGS can be represented by the summarized expression of its genes and subsequently tested for association with other phenotypes or used in unsupervised analyses.

**Figure 1:**
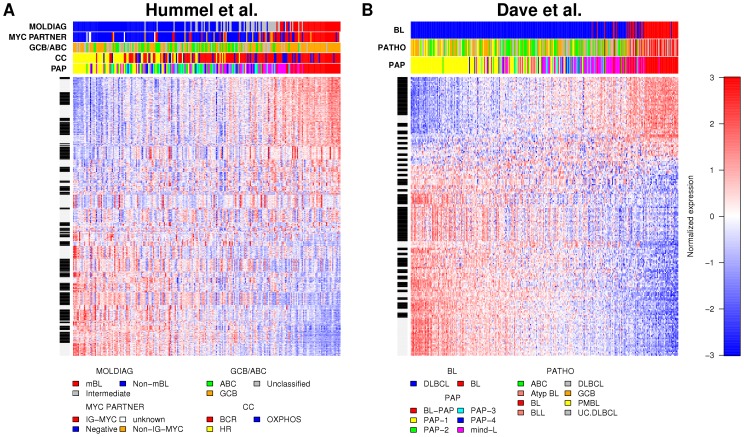
Sets of co-expressed genes reproduce the distinction between Burkitt lymphomas and other types of mature aggressive B-cell lymphomas. Heat maps of 50 CGSs generated in two independent BL/DLBCL data sets: (A) Hummel et al. (2006), n = 220 (GSE4475) and (B) Dave et al. (2006), n = 303 (GSE4732). Each row shows a profile of a gene and genes are grouped into gene sets as indicated by white and black intervals on the vertical bar left. Red represents high expression and blue represents low expression relative to the mean over all samples. Each column corresponds to one sample. Samples are annotated by horizontal color bars above the heat maps. The samples and the gene sets are ordered by the values of their 1^st^ principal component. Annotations from Hummel et al. (2006): “MOLDIAG”: classification into molecularly defined Burkitt lymphoma (mBL), intermediate cases and non-mBL cases, “GCB/ABC”: cell of origin (molecular classification), “MYC PARTNER”: information about the absence (“Negative”) or presence of *MYC* breakpoints and about the translocation partner of *MYC* (IG or non-IG). “CC”: “consensus clusters” [3], [6]. Annotations from Dave et al. (2006): “BL” diagnosis of Burkitt lymphoma or DLBCL based on gene expression, “PATHO”: pathological diagnosis or molecular diagnosis in case of samples analysed prior to Dave et al. (2006). “PAP”: “pathway activation patterns” [6] (available for both data sets [4], [5]).

To validate the method, we first applied CGSA to a large, previously analyzed gene expression data set containing Burkitt lymphomas and DLBCLs (termed “BL/DLBCL data set of Hummel et al. (2006)” [4]). An unsupervised analysis with respect to the generated CGSs was performed to investigate whether the new method was sensitive enough to identify the already well-established signatures. Indeed, CGSA reproduced several previously reported molecular classifications, including the molecularly defined Burkitt lymphoma [4], [5] (Figure 1A, R ^2^ = 0.59, AUC  = 0.99), the activated B-cell-like (ABC) and the GC B-cell-like (GCB) subtypes [1] (Figure S2A, R^2^ = 0.58, AUC  = 0.95) and several of the PAPs [6] (Figure S1A, R^2^ = 0.72, AUC  = 0.82, all adjusted P = 0.001). An independent analysis of another data set (termed “BL/DLBCL data set of Dave et al. (2006)” [5]) corroborated these results (BL vs. DLBCL: Figure 1B, R ^2^ = 0.53, AUC  = 0.98, ABC vs. GCB: Figure S1D, R^2^ = 0.62, AUC  = 0.96, PAPs: Figure S1D, R^2^ = 0.75, AUC  = 0.83, all adjusted P = 0.001; see Text S1 Section 6a,b for details).

Moreover, we proved the robustness of the approach by splitting the BL/DLBCL data set of Hummel et al. (2006), after which unsupervised orderings of samples showed high correlation (Spearman's r = 0.94, r = 0.99, P<1e-5, Text S1 Section 6c). In an analysis across data sets and platforms, using the BL/DLBCL data sets of Hummel et al. (2006) and of Dave et al. (2006), we also observed good reproducibility of the orderings of samples as measured by their association with previously defined molecular subclasses (Figures S1 and S2, Text S1 Section 6d).

### Definition and characterization of the CGSs in diffuse large B-cell lymphoma

Having established the validity of the CGSA on the two BL/DLBCL data sets [4], [5], we applied it to a data set of 364 DLBCL and related mature aggressive B-cell lymphomas other than Burkitt lymphoma (termed “extended DLBCL data set”) [15]. This cohort included 150 cases from the BL/DLBCL data set of Hummel et al. (2006) and 214 additional cases. We focused on DLBCL because our aim was to unravel its high heterogeneity. Since expression patterns within the DLBCLs might be difficult to discern in the presence of the strong contrast with Burkitt lymphoma we decided to exclude this entity.

In the first step of the analysis, we generated 50 new sets of correlated genes (CGSs) in the extended DLBCL data set using exactly the same procedure as applied before to the BL/DLBCL data sets of Hummel et al. (2006) and Dave et al. (2006) (File S1). The CGSs were named according to their “central” gene. Then, we searched for significant relationships between each gene set and a number of available phenotypic and genetic variables. Significant associations were found between some of the gene sets and sex (sex correlated with a set of Y chromosome genes), age, tumor cell content, proliferation (Ki67 index), genetic aberrations (deletions in 6q21, 17p13, presence of t (14;18)), immunophenotype (MUM1, CD10 expression) and the cell of origin (ABC/GCB), respectively (File S2). Several gene sets were characterized by striking enrichment of genes related by their function or cellular and genomic location. For example, CGS 1 (with central gene POSTN) contained mostly genes encoding proteins of the extracellular matrix, CGS 7 (with central gene HIST1H2BK) consisted of the histone cluster 1 genes on 6p21, CGS 8 (HLA-DQA1), CGS 11 (HLA-DQB1) and CGS 16 (HLA-DRB4) were composed of major histocompatibility complex (MHC) class II genes located on 6p21 while CGS 40 (HLA-F) contained MHC class I genes from this chromosome arm. Interestingly, CGS 29 (NUSAP1) was enriched in genes located on chromosome 15q (File S3) although no recurrent genomic aberrations in this region were detected. In summary, several CGSs picked up signals related to chromosomal assignments and functional similarities.

In order to investigate how the 50 CGSs were related to each other, we explored two- and three-dimensional principal component biplots [19] (Figure S4, File S4). Biplot is a plot which represents the samples (here: the tumor samples) and the variables (here: the CGSs) of a data matrix on the same plot. These analyses showed that several of the CGSs could be grouped into three major components. Remarkably, these components were biologically interpretable by the characteristics of their constituent CGSs. In the first component, CGS 6 (MAD2L1), CGS 12 (NME1), CGS 29 (NUSAP1) and CGS 48 (CDC6) were significantly associated with the Ki67 index indicating a link to proliferation (File S2). In the second component, CGS 2 (C1QB), CGS 19 (GZMB), CGS 31 (CD8A) and CGS 34 (CD3E) correlated (R^2^>0.25, File S2) with the tumor cell content or its counterpart, i.e., the amount and function of bystander cells. CGS 2 (C1QB) for which this correlation was strongest was highly enriched in the GO-term “immune response” (P<1e-10, File S3). In the third component, CGS 1 (POSTN) which exhibited significant overlap with the GO-term “extracellular matrix” (P<1e-10) was tightly correlated with CGS 15 (GJA1) (r = 0.88) and CGS 33 (PCOLCE) (r = 0.9). These observations indicated that the three groups of CGSs strongly determining the heterogeneity of the DLBCLs were characterized by a proliferation signature, immune response and stromal signature, respectively. This was additionally supported by an analysis of the overlap between several literature based signatures and the CGSs (File S1). Therefore, our observations were consistent with those from earlier reports [2], [20]. The characteristics of all CGSs are summarized in File S5 and correlations among the CGSs are shown in File S6.

### Unsupervised analysis reveals three subgroups of DLBCL tumors

**Figure 2:**
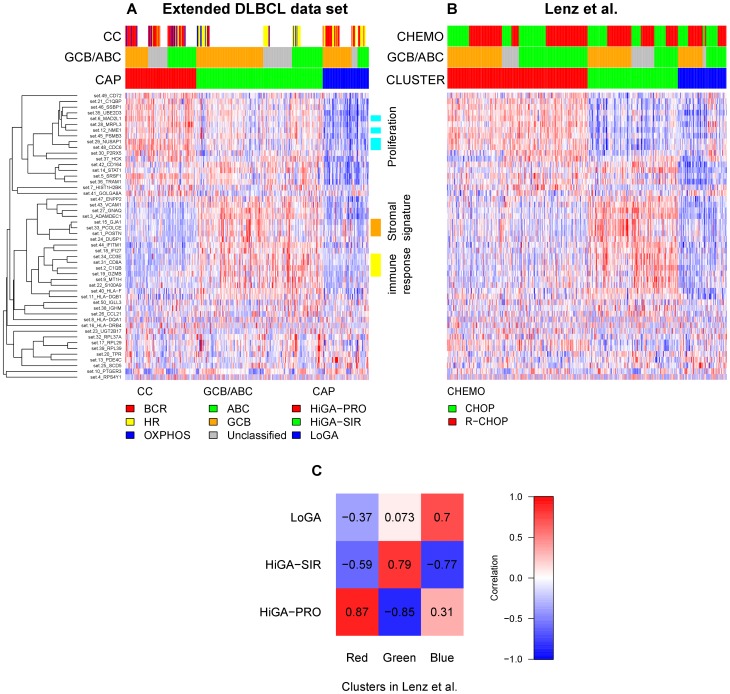
Unsupervised analysis with respect to the CGSs reveals three profiles across previously described molecular subtypes of DLBCL tumors. The three profiles are also detectable in an independent data set. A) Heat map of 50 CGSs generated in the extended DLBCL cohort (n = 364). Each row is a summary value of a CGS and each column corresponds to a sample. Red (blue) indicates a high (low) relative expression. Samples are grouped into HiGA-PRO (red), HiGA-SIR (green) and LoGA (blue). CGSs are hierarchically clustered using average linkage and one minus correlation as the distance measure. The color bars on the right indicate three groups of CGSs associated with proliferation, stroma and immune response, respectively. White spaces in the color bar “CC” are due to the fact that the classification into “consensus clusters” was available only for the 150 cases which overlapped with the BL/DLBCL data set of Hummel et al. (2006). B) Clustering (indicated by the color bar “CLUSTER”) of an independent data set of Lenz et al. (2008) (n = 414) with respect to the CGSs mapped from the extended DLBCL data set. The vertical order of the CGSs is the same as in Panel A. Within the clusters, the samples are sorted by their ABC/GCB/Unclassified subtype and by their treatment (CHOP, R-CHOP). C) Correlation matrix of the centroids of the CAPs and of the clusters generated in the data set of Lenz et al. (2008).

Next, we asked whether we could find discrete subgroups of DLBCL tumors which would show different expression profiles of the 50 CGSs. Indeed, unsupervised analysis of the samples with respect to the CGSs identified three profiles which we refer to as HiGA-PRO (high gene activation with proliferative phenotype), HiGA-SIR (high gene activation with stromal and immune response) and LoGA (low gene activity) profiles and term collectively as CAPs (CGS activation profiles) (Figure 2A, Materials and Methods).

HiGA-PRO was characterized by high expression of the CGSs which were significantly associated with proliferation (Ki67 index), and low expression of the CGSs related to stroma and immune response (Figure 2A). HiGA-SIR exhibited intermediate proliferation and high immune response while the expression of the stromal CGSs was relatively high but differed between the ABC and the GCB DLBCLs. Interestingly, expression of most CGSs in LoGA was lower than in the tumors of the other two CAPs (CGS activation profiles).

To assess reproducibility of the CAPs we mapped the CGSs to an independent data set of 414 DLBCLs (termed “data set of Lenz et al. (2008)”) [20]. Generating three clusters in this data set clearly recapitulated the patterns of expression of the 50 CGSs seen in the CAPs (Figure 2B,C, Text S1).

**Figure 3:**
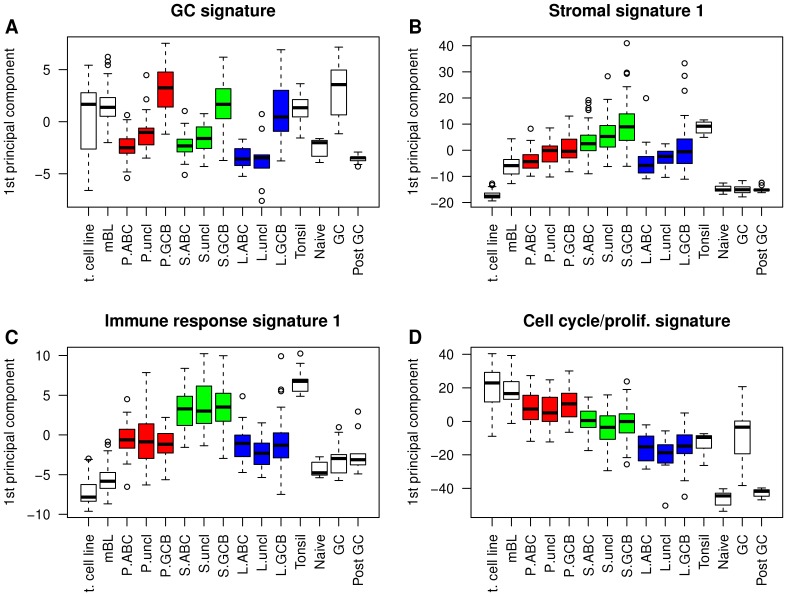
Expression patterns of published signatures indicate that the CAPs provide additional information to the ABC/GCB classification. Shown are box plots of the several published signatures. Genes within each signature are summarized by their first principal component. Samples from our DLBCL cohort are split into the CAPs (“P”: HiGA-PRO, “S”: HiGA-SIR, “L”: LoGA) and the ABC/GCB subtypes. Other sample groups are shown for comparison. They include mBLs from the BL/DLBCL data set of Hummel et al. (2006), normal naïve B cells, germinal center (“GC”) B cells, post GC B cells, normal cells from tonsils and malignant cell lines (“t. cell line”) (Materials and Methods).

Several gene expression signatures published in the context of DLBCL showed patterns of expression across the CAPs which were unrelated to their patterns between the ABC and the GCB DLBCLs (Figure 3). For example, the immune response signature 1 [21] and the cell cycle/proliferation signature [22], [23] were equally expressed in the ABC and GCB DLBCLs (P = 0.975, P = 0.849, respectively) but exhibited strong differences between HiGA-PRO, HiGA-SIR and LoGA (P = 1.63e-32, P = 2.93e-34, respectively). In contrast, the levels of the GC signature [20] differed clearly between the ABC and GCB DLBCLs (P = 2.33e-50) but much less so among the CAPs (P = 2.98e-05). Interestingly, the levels of the stromal signature 1 [20] varied with respect to both characteristics, i.e., the cell of origin and the CAPs (GCB/ABC: P = 2.84e-09, CAPs: P = 8.71e-16, Materials and Methods). These observations demonstrated that the CAPs captured additional transcriptional variation not accounted for by the classification into the ABC and GCB lymphomas although we could observe an overrepresentation of the ABC DLBCLs in HiGA-PRO and of GCB DLBCLs in HiGA-SIR and in LoGA.

Samples previously classified [3], [6] as belonging to the “host response” (HR) cluster were enriched in HiGA-SIR while HiGA-PRO bore similarity to the “BCR/proliferation” [3] cluster. Interestingly, LoGA did not resemble any of the previously identified consensus clusters [3], [6]. In particular, the “OxPhos” cluster and LoGA were non-overlapping.

We observed differences in survival among the CAPs, with the HiGA-SIR being more favorable (5-year survival rates: HiGA-PRO 35% (95% CI: 26%–49%), HiGA-SIR 61% (54%–70%), LoGA 40% (28%–57%), Figure S5). However, we were unable to confirm this result in the corresponding clusters which we found the data set of Lenz et al. (2008) [20]. Nevertheless, some trends could be seen across the data sets. In particular, LoGA and the corresponding blue cluster in the data set of Lenz et al. (2008) [20] demonstrated within the ABC DLBCLs the most unfavorable outcome of all three subtypes in our cohort, in the CHOP-treated and in the R-CHOP-treated cohort of Lenz et al. (2008) [20] (Figure S5).

Further information on the incidence of a number of biological features in the CAPs is provided in Table S1.

### HiGA-PRO and HiGA-SIR but not LoGA show large scale transcriptional activation

**Figure 4:**
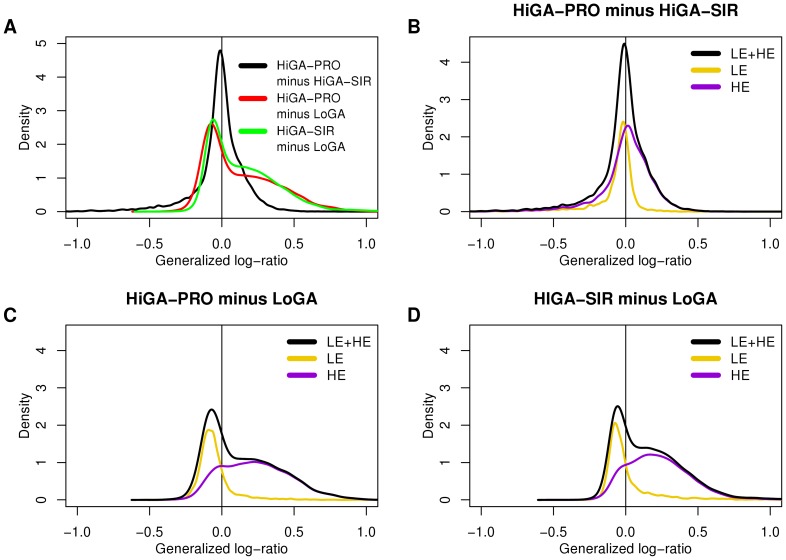
HiGA-PRO and HiGA-SIR show a massive transcriptional activation compared to LoGA. This activation can be attributed to the HE genes but not to the LE genes. No such massive differences in expression can be seen between HiGA-PRO and HiGA-SIR. Shown are densities (kernel density estimates of the distributions) of gene-wise generalized log-ratios (estimated log fold changes) between the CAPs. A) The three densities correspond the contrasts: HiGA-PRO minus HiGA-SIR (black line), HiGA-PRO minus LoGA (red line) and HiGA-SIR minus LoGA (green line). Each density includes all probe sets with unique Entrez IDs (n = 12679). B,C,D) Densities of the estimated log fold changes of the HE and LE genes in the three contrasts from A. The densities are scaled such that the sum of the density of the LE genes (yellow line) and the HE genes (violet line) equals the density of LE and HE genes together (black line). In B, C and D only probe sets with unique Entrez IDs which could be classified to the LE (n = 3585) or HE (n = 7325) groups are included.

The surprisingly low activity of most CGSs in LoGA prompted us to examine its expression profile more closely. An analysis of differential expression between the CAPs with respect to all genes revealed that, consistent with the behavior of the CGSs, there were more upregulated than downregulated genes (5730 and 3325, respectively) in the comparison of HiGA-PRO with LoGA. A similar observation applied to the comparison of HiGA-SIR with LoGA (6433 upregulated and 2835 downregulated genes, false discovery rate (FDR)  = 0.05). Moreover, absolute log fold changes of the genes which were overexpressed in HiGA-PRO relative to LoGA or in HiGA-SIR relative to LoGA were larger than those of the genes downregulated in these comparisons (Figure 4A). In addition to this striking asymmetry, histograms of the fold changes (Figure 4A) suggested existence of two large groups of genes, one of which was upregulated in HiGA-PRO and HiGA-SIR and the other which remained essentially constant across the CAPs.

In the next step of our analysis, we examined the overall distribution of expression values of all genes and samples split into the CAPs. We could see a marked difference between LoGA and the other two profiles (Figure S6A). Importantly, no difference in the overall distribution of expression values could be observed between HiGA-PRO and HiGA-SIR, even though there was a large number of differentially expressed genes between these entities (2916 upregulated and 3090 down regulated genes in HiGA-PRO compared to HiGA-SIR, FDR  = 0.05). To look at a wider context, we made use of data from various types of non-malignant B cells, from mBL tumors [4] and from B-cell lymphoma cell lines. Global distribution of expression in these samples suggested similarity between LoGA and the non-malignant B cells while HiGA-PRO and HiGA-SIR seemed to be more similar to the lymphoma cell lines (Figures S6B–D). Thus, these analyses indicated that HiGA-PRO and HiGA-SIR show large scale transcriptional differences compared to LoGA. Moreover the global expression profile of LoGA tends to resemble that of mature non-malignant B cells.

### Genes shown to carry activating histone marks are associated with the transcriptional activation in HiGA-PRO and HiGA-SIR

To investigate the possible mechanisms responsible for the observed massive difference in expression between LoGA and the remaining DBLCLs, we examined two recently described [16] major classes of genes, termed “lowly expressed” (LE) and “highly expressed” (HE) genes. The two classes had been shown to differ with respect to their mRNA abundance in a broad range of metazoan cells including those of human, mouse and Drosophila. First, we verified that in our data the two classes of genes exhibited clearly different expression levels in a similar way as observed in the original study [16] (Figure S7). Next, we examined differential expression of these groups of genes among the CAPs. The division into the LE and the HE genes explained the shape of the distributions of the estimated log fold changes strikingly well (Figure 4B–D). This analysis also clearly demonstrated that the large scale transcriptional activation in HiGA-PRO and HiGA-SIR could be attributed to the HE genes but not to the LE genes. The LE genes exhibited only a quantitatively small shift towards higher expression in LoGA (median estimated fold change, LoGA vs. HiGA-PRO: 1.08, LoGA vs. HiGA-SIR: 1.05).

**Figure 5:**
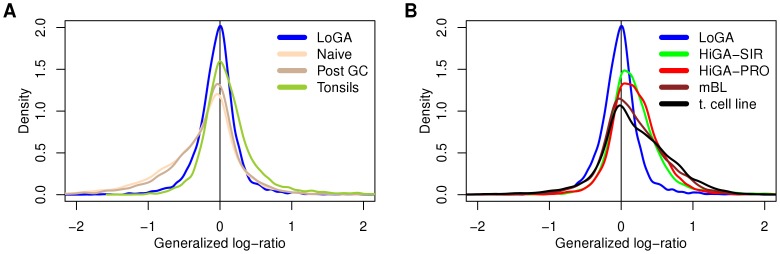
Distributions of the estimated log fold changes of expression levels of the HE genes relative to the normal GC B cells. Shown are densities (kernel density estimates of the distributions) of gene-wise generalized log-ratios of the HE genes between the mean expression of several groups of samples and that of the normal GC B cells. A) Densities corresponding to LoGA and the normal cells. B) Densities corresponding to LoGA and other tumor samples.

To gain an overview of the differences in expression of the LE and HE genes between the CAPs, normal cells, tumor cell lines and mBLs we plotted the estimated log fold changes of all subgroups relative to the normal GC B cells (Figure 5, Figure S8). The LE genes showed virtually no differential expression, consistent with the hypothesis of Hebenstreit et al. [16] that the LE genes are expressed at a very low level and are putatively non-functional. The HE genes exhibited more diverse patterns. First, in the naïve and post GC B cells a number of transcripts were expressed at a lower level as compared to the GC B cells. Second, the HE genes in HiGA-PRO and HiGA-SIR were shifted towards higher expression. And third, in the mBLs and in the B-cell lymphoma cell lines there seemed to be an additional upregulation of a substantial fraction of the HE genes as compared to normal GC B cells. Most strikingly, the log fold changes for LoGA were small and centered at zero, suggesting a lack of a deregulation of a global-scale transcriptional program which is present in the other types of mature aggressive B-cell lymphomas.

In the original work [16] describing the HE and LE genes, the HE genes were shown to be associated with the presence of activating histone marks (H3K9/14ac). In order to explore whether altered histone modification could be associated with the identified CAPs, we used immunohistochemical staining for H3K4me2, H3K27me3 and H3K18ac in 220 cases of the cohort for screening. No significant association was observed at the immunhistochemical level. In contrast, EZH2 Y641 mutation was more frequent in our dataset in LoGA than in the other DLBCLs (P = 0.032, Table S1). EZH2 (Y641F/N) promotes trimethylation of H3K27 which is a posttranslational histone modification associated with repression of transcription.

### Metabolic processes are commonly upregulated in HiGA-PRO and HiGA-SIR

**Figure 6:**
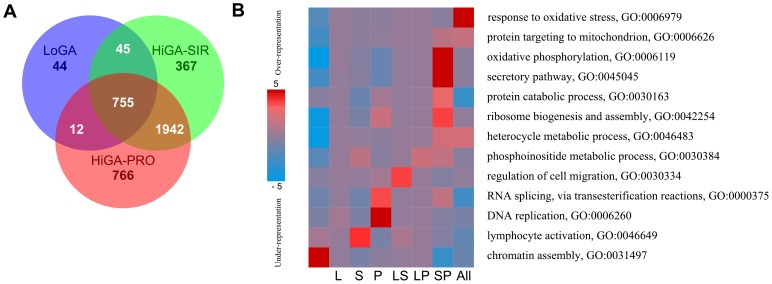
Overexpressed HE genes in the CAPs relative to normal GC B cells. A) Venn diagram showing the intersections of the overexpressed HE genes (FDR <0.05) in each CAP relative to normal GC B cells. B) Enriched GO-terms from the category Biological Process according to an analysis with PAGE. The columns in the heatmap refer to the subsets of the venn diagram, e.g., column “L” refers to the genes which were uniquely overexpressed in LoGA (N = 44), “LS” to the genes which were overexpressed in LoGA and HiGA-SIR (N = 45), “SP” to the genes which were overexpressed in HiGA-SIR and HiGA-PRO and “All” to the genes commonly overexpressed in all the CAPs. The first column corresponds to the non-overexpressed genes (FDR >0.05). Rows correspond to GO terms. Red entries in the heatmap indicate overrepresentation of the GO-terms (measured by negative log10 p-values) while blue entries indicate underrepresentation (measured by log10 p-values).

Finally, we asked which known cellular processes were active in the different CAPs. To this end, we computed the overexpressed HE genes in each CAP relative to the normal GC B cells. The intersections of the three resulting lists of overexpressed genes are shown in form of a venn diagram in Figure 6A. We observed that only few genes were exclusively upregulated in LoGA. In contrast, a large number of genes were specifically overexpressed in HiGA-PRO and HiGA-SIR, respectively. Analysis [24] of the biological functions of the genes in each region of the venn diagram (Figure 6B, File S7) revealed a strong enrichment in oxidative phosphorylation (P = 4.7e-12) among the genes upregulated in HiGA-PRO and HiGA-SIR but not upregulated in LoGA (Figure 6B, Figure S9, File S7, Text S1). These genes were also enriched in secretory pathway (P = 4.1e-07), ribosome biosynthesis (P = 2.95e-04) and protein catabolic process (P = 1.4e-03). Several GO terms were overrepresented in other regions of the venn diagram including response to oxidative stress (P = 1.1e-06) among the genes which were upregulated in all CAPs, DNA replication (P = 2.7e-06) and RNA splicing (P = 3.4e-04) among the genes specifically upregulated in HiGA-PRO, lymphocyte activation (P = 1.4e-04) among the genes unique to HiGA-SIR, and regulation of cell migration (P = 2.6e-04) among the genes overexpressed in HiGA-SIR and LoGA but not overexpressed in HiGA-PRO. Interestingly, chromatin assembly was depleted among the HE genes which were overexpressed in any of the CAPs (P = 6.85-07), and in particular among the genes commonly upregulated in HiGA-PRO and HiGA-SIR (P = 8.0–04). Taken together, these data suggest that a high level of metabolic activation is a common feature of HiGA-PRO and HiGA-SIR but not of LoGA.

## Discussion

In our investigation, we developed and used a biostatistical method (CGSA) for identifying sets of highly correlated genes [25]. The method permitted an unsupervised search for dominant and independent metagenes useful for tumor profiling. Each gene set comprised genes which were coordinately expressed across all specimens. Genes from different gene sets were mutually exclusive. We could show that 50 such gene sets (containing 501 genes, File S1) carry important information regarding previously described tumor signatures. The method clearly detected in an unsupervised way the signature of the molecular Burkitt lymphoma which was described by us and others using more indirect strategies [4], [5]. Furthermore, we also found the previously reported classification into the ABC and GCB lymphomas [1], [2] and several of the “pathway activation patterns” [6]. Having shown that our unsupervised method was sensitive enough to discriminate these known subtypes, we applied it to unravel novel layers of transcriptional profiles in a large data set of DLBCLs from which molecular Burkitt lymphomas had been excluded. In this analysis we also used data from tumor cell lines, from normal B cells and from normal tonsil tissues. By using the CGSA-method we were able to group these lymphomas into three profiles.

Our method has a series of special features (Materials and Methods, Text S1). The guiding principle of the method is that every aspect of the construction and filtering of the CGSs depends solely on permutation-invariant statistics such as the overall variance and covariance of the genes. As a consequence, the probability that we find any false positive association of the CGSs with other characteristics of the patients is kept at a prespecified low level [26]. The CGSs are not necessarily orthogonal to each other as, e.g., in principal component analysis. Therefore, they adapt in a more flexible way to the correlation structure of the genes. Furthermore, the method facilitates biological interpretation by reducing the high dimensional space containing several thousands of genes to a small number of gene sets. Hence, the gene sets capture differential regulation of major gene hubs and, as our analysis indicated, the inferences based on them are robust across data sets and platforms. Importantly, the dimension reduction is not guided by any external criteria and therefore allows for an unbiased view of the data. Finally, our recent research suggests that the CGSs can be used as a basis to construct pathway activation indices. This extension is beyond the scope of this study but it has already been successfully applied elsewhere [27].

**Figure 7:**
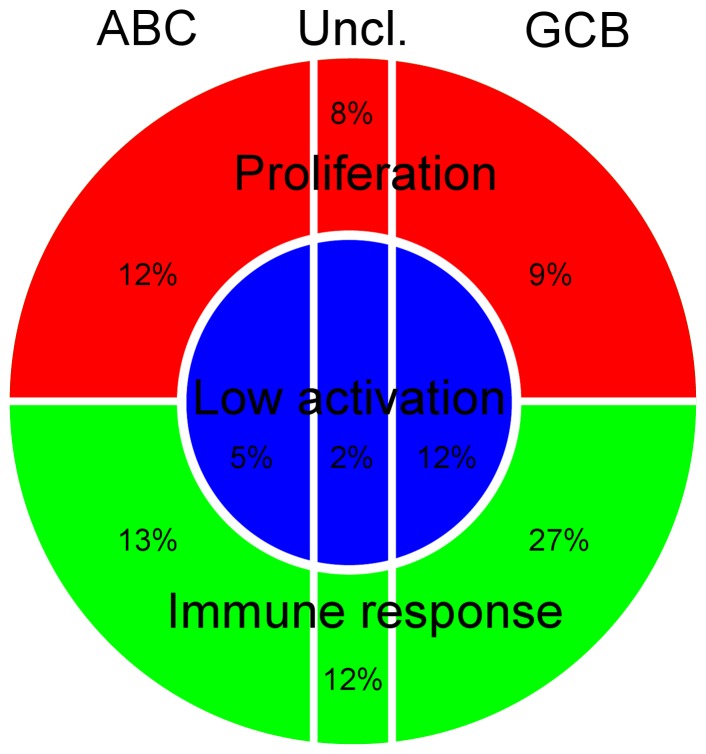
Conceptual scheme of the gene expression heterogeneity in the DLBCLs. In our data set of 364 DLBCL and related mature aggressive B-cell lymphomas other than Burkitt lymphoma we observed about 19% of the tumors with the LoGA profile (blue center), about 52% with the HiGA-SIR profile and about 29% with the HiGA-PRO profile (green and red shell). Every profile contained samples classified as ABC and GCB DLBCLs. The horizontal direction corresponds to the contrast between these subgroups, the vertical direction discriminates the DLBCLs into those with either a proliferative or immune response phenotype. The radial direction from the center of the circle outward represents the global transcriptional activation.

The three profiles which we found with our method in the extended DLBCL cohort (n = 364) were distinct from the classification into the ABC and GCB lymphomas. Figure 7 contains a schematic summary of our findings.

One profile, which we referred to as HiGA-PRO was characterized by high tumor cell content and CGSs related to cell cycle, DNA replication and RNA splicing, suggesting intensive proliferation. On the large scale level several hundred genes of the mitochondrial respiratory chain, nucleoside biosynthesis and metabolic pathways were activated. This is compatible with the needs of high cell turnover. In this profile, both ABC and GCB cells of origin could be found with almost equal frequency. The HiGA-PRO profile was similar to the “BCR/proliferation” consensus cluster [3] and was characteristic of about 29% of our DLBCL cases.

The second profile, HiGA-SIR was characterized by lower tumor cell content and a marked stromal and immune response phenotype. On the large scale level this profile was also linked with high metabolic activation of genes involved in the mitochondrial respiratory chain, nucleoside biosynthesis and other metabolic processes. In this profile both the GCB and ABC subtypes could be found with a clear preponderance of GCB (2 to 1). This profile was similar to the “host response” consensus cluster [3] and was present in about 52% of our cohort. The depletion of *MYC* translocations (Table S1) in HiGA-SIR and its favorable patient outcome in our extended DLBCL cohort (Figure S5) indicated that the lower tumor cell content in HiGA-SIR was likely due to the underlying biology of the tumor cells and not to the way in which the tumor samples were collected. It is also noteworthy that Monti et al. (2005) [3] observed younger patients in their “host response” cluster compared to their “BCR/proliferation” cluster. We could make a similar observation (*P* = 0.007, Mann-Whitney U test) by comparing HiGA-SIR with HiGA-PRO.

The third and novel profile was markedly different and found in about 19% of the cases. It exhibited lower gene activation compared to the two other profiles. Hence we called it LoGA (i.e., low gene activity). There were few indications of mitochondrial metabolic or biosynthetic activation in LoGA beyond the level also encountered in normal B cells. Intriguingly, these tumors were characterized by high tumor cell content, high genetic complexity and active proliferation seen in the Ki67-indices (Figure S10A–C). This suggests that these tumors obtain sufficient supply of energy to proliferate at least partly through other means than mitochondrial activation.

As shown in the enrichment analysis (Figure 6B) all tumors exhibit signals of strong oxidative stress when compared with normal B cells. This may be indicative of a stress climate facilitating mutations in all tumors consistent with the finding of high genetic complexity. Based on these data we speculate about an evolutionary model of DLBCL diversification. It is well established that partly distinct genetic lesions occur in the B cells of origin leading to a GCB-phenotype or to the ABC-phenotype [2], [13]. These events may be the initiating steps. It is tempting to speculate that lymphomagenesis progresses by accumulating and selecting further mutations affecting the cellular differentiation program by rendering cells dependent (HiGA-SIR) or independent (HiGA-PRO) from immune response and stromal interactions. Further genetic and epigenetic mutations may promote and stabilize these patterns by activating the necessary energy and biosynthesis machinery resulting in activation of hundreds of genes in the two HiGA-phenotypes. This hypothesis of a prominent role of energy metabolism for a subset of DLBCLs is supported by recent experimental evidence [28]. However, the newly found LoGA pattern would be indicative of alternative pathways of tumor evolution not leading to high metabolic and oxygen dependent activation.

In two very recent studies Myc has been shown to act as a universal amplifier of expressed genes in a variety of cells including lymphocytes [29], [30]. One could ask whether this effect of Myc could explain the massive shift in transcriptional activity observed in our data between LoGA and the two other profiles. We do not have indications that this might be the case. In particular, *MYC* translocations do not occur less frequently in LoGA (16%) than in HiGA-PRO (13%) and HiGA-SIR (5%).

Our observations have methodological, clinical and biological implications. The biostatistical strategy presented in this study is general and can be applied to gene expression profiling data in different experimental settings. From a clinical perspective it might be interesting to investigate whether the LoGA-tumors are associated with different fludeoxyglucose- (FDG) or amino acid positron emission tomography (PET) profiles than the HiGA-tumors, a question which can be investigated if PET and expression profiling data are available. From the biological perspective, our work shows the need for further research to clarify the underlying causes responsible for the observed massive transcriptional changes in subgroups of diffuse large B-cell lymphoma. This question might be addressed by ongoing or future genome wide sequencing studies.

## Materials and Methods

### Generation of correlated gene sets (CGSs)

To create the CGSs, each gene was treated as a center of a gene set and all genes which were strongly and positively correlated with this central gene were included in this set (including negatively correlated genes would be possible, but in our experience, such genes were rare). Thus, as many candidate gene sets as there were genes in the data set were generated. Next, the gene sets were ranked by a score which reflected their size, the variances of their central genes and how tightly correlated were their member genes with their respective central genes. Then, gene sets which overlapped with any of the higher-ranking gene sets were removed and the top 50 of the remaining gene sets were selected. Finally, a summary of the gene expression values within each gene set was computed. Thus, the dimension of each analyzed data set was reduced from the initial several thousands of features to 50 CGSs. We found in pretests (Figure S3, Text S1) that this number was enough to be able to capture the main factors present in the data set providing at the same time sufficient statistical stability. The method presented here and subsequently called Correlated Gene Set Analysis (CGSA) is an extension of our previously described strategy [25], [26]. Further details of the method are given in Text S1.

### Study population and microarray data

For validating the CGSA method, we used two previously published data sets of mature aggressive B-cell lymphomas [4], [5] (“BL/DLBCL data sets of Hummel et al. (2006) and of Dave et al. (2006)”) as available at Gene Expression Omnibus (http://www.ncbi.nlm.nih.gov/geo/, GEO accession numbers GSE4475, GSE4732, respectively). In the subsequent, main part of our study, we analyzed a cohort consisting of 364 DLBCL and related mature aggressive B-cell lymphomas other than Burkitt lymphoma (“extended DLBCL cohort”) [15]. This cohort included 150 cases from one of the data sets which we used for validating our method (BL/DLBCL data set of Hummel et al. (2006)) and 214 additional cases originally published in two further studies [31], [32] (GEO accession numbers GSE10172 and GSE22470, respectively). We annotated the samples with values of gene expression signatures according to previous publications. These signatures were: the cell-of-origin signature (ABC, GCB) [2], the molecular Burkitt signature [4], the PAP signature [6] and, for the samples from the BL/DLBCL data set of Hummel et al. (2006), the consensus clusters (CC) [3], [6].

Array-based comparative hybridization data (CGH) was available [15] for 273 cases out of the 364 cases of the extended DLBCL data set. The selection of recurrent copy number aberrations was described previously [15], [33].

All cases of the extended DLBCL cohort (n = 364) were collected within the network project Molecular Mechanisms in Malignant Lymphoma (MMML). For validation of the CAPs, we used an independent data set of 414 DLBCL samples [20]. We obtained raw expression data from the GEO (accession number GSE10846) and normalized and summarized them using a similar procedure to that applied to our data from the MMML-project.

### Gene expression of normal cells and lymphoma cell lines

New gene expression data from normal cells and lymphoma cell lines included: 8 samples of naïve B cells, 13 samples of germinal center (GC) B cells, 9 samples of post GC B cells, 10 tissue samples of tonsils and 32 samples of 28 different lymphoma cell lines [34]. Whereas the 10 tonsillar tissue samples were used as whole tissue RNA extracts, the B cell subsets (naïve B cells and post GC (memory) B cells) were isolated from peripheral blood samples of healthy individuals. GC B cells were isolated from suspended tonsillar cells. For isolation of the B cell subsets, FACS sorting employing antibodies against CD19 and IgD (naïve B cells), CD20 and CD38 (tonsillar GC B cells), and CD19 and CD27 (post GC memory B cells) was used.

Affymetrix hybridization to U133A GeneChips was performed according to manufacturer's recommendations as already described [4]. All raw expression data from the MMML-project including the data of the normal B cells and lymphoma cell lines were normalized using the VSN method [35] and the probe-level data were summarized using median polish [36]. The parameters for VSN and median polish were estimated on the samples included in the BL/DLBCL data set of Hummel et al. (2006) and applied to the remaining samples [37]. The new data were deposited at Gene Expression Omnibus (GEO, http://www.ncbi.nlm.nih.gov/geo/), accession number GSE43677.

### Tissue microarrays for histone modifications

Tissue microarrays (TMAs) containing 220 cases from the MMML cohort constructed by the Institute of Pathology, Section Hematopathology and Lymph Node Registry, University Hospital Schleswig-Holstein, Kiel, Germany, were studied herein. The sections were deparaffinized in xylene and rehydrated in graded alcohols. Endogenous peroxidase was quenched with 1% hydrogen peroxide in methanol for 10 minutes. The antigen was retrieved in 0.01 M sodium citrate buffer (pH 6.0). The sections were incubated for 1 hour at room temperature with H3K27me3 mouse mAb (Abcam [ab6002] at 1∶25), H3K4me2 rabbit mAb (Abcam [ab32356] at 1∶50) and H3K18ac rabbit mAb (Abcam [ab40888] at 1∶25). The sections were then treated with N-Histofine^®^ (Nichirei Biosciences, Japan) polymer detection system, chromogen detection with diaminobenzidine (DAB) and counterstaining with Mayer's hematoxylin. Negative control staining was performed without primary antibody. Two pathologists assessed the percentage of tumor cells with positive nuclear staining independently based on the following scoring criteria: 0 (negative), 1 (1–25%), 2 (26–50%), 3 (51–75%), and 4 (76–100%).

### Other genetic and phenotypic data

Recently published phenotypic data was available [15]. These included data from immunohistochemical staining against CD10, CD5, BCL2, BCL6, MUM1, Ki67, data from interphase fluorescence in situ hybridization (FISH) for breakpoints in MYC, BCL6 and immunoglobulin (IG) partners, IGH/BCL2 fusion, overall survival for 282 cases, morphology (centroblastic, immunoblastic, other), tumor cell content, age and gender. Lymphomas with MYC breaks were divided into two categories depending on whether MYC was fused to one of the IG-loci (“IG-MYC”) or not (“non-IG-MYC”). Detailed information on the immunohistochemical staining, FISH and on the incidence of the biological features in the study population was provided previously [15]. The content of tumor cells (percentage of all cells) was estimated in all lymphoma specimens by means of immunohistochemistry for the detection of CD20, CD3 and CD68 on frozen sections. Tumor cell content ranged from 50% to 95% (in 134 of 136 specimens it exceeded 60%), with a median of 85%.

### Unsupervised analysis with respect to the CGSs

For validating the CGSA method on the BL/DLBCL data sets of Hummel et al. (2006) and Dave et al. (2006), a previously published method for unsupervised ordering of samples was used [25]. Briefly, the method projected all samples onto a two dimensional plane spanned by their first two principal axes. Then, the samples were ordered by their angular distance from an arbitrary vector in this plane. A slightly modified version of this method was used to arrange samples of normal B cells and lymphoma cell lines among the tumor samples (Text S1).

To generate the CAPs (CGS activation profiles) in the extended DLBCL data set (n = 364), partitioning around medoids (PAM) [38] algorithm was applied to the 50 CGSs. Euclidean distances with respect to standardized CGSs were used and the number of clusters which maximized the average silhouette width [39] was chosen. The result of the clustering is given in File S8.

### Statistical analyses

The available phenotypic features of the samples were categorized as previously described [15]. The procedure of testing their associations with the CGSs is given in Text S1. Overall survival was defined as time from first day of therapy to death from any cause. Patients without an event in OS were censored at the last day with valid information. Overall survival was estimated by the Kaplan-Meier method and compared using the log-rank test. Tests for association of the ABC and GCB subtypes and the CAPs with several published signatures (Figure 3) were performed using two-way ANOVA with the first principal component of a signature as a dependent variable. Unless otherwise indicated, the computations were carried out using the statistical software R [40] and Bioconductor [41].

## Supporting Information

Figure S1 **CGSs discriminate BL/DLBCLs according to several previously reported molecular classifications.** This result is reproducible across different BL/DLBCL data sets. Heat maps (A) and (D) show expression of the 50 CGSs generated in the BL/DLBCL data set of Hummel et al (2006) and Dave et al (2006), respectively. Heat maps (B) and (C) show the CGSs from the heat maps (A) and (D), respectively, mapped to the other data set. Samples (columns) and gene sets (rows) are arranged in the angular order of their projections onto the plane spanned by the first and the second principal axes (Text S1). This plane is determined in the data set where the CGSs were created and is used to order the samples in the original data set and in the other data set.
(TIF)

Figure S2 **The CGSs generated in the BL/DLBCL data set of Hummel et al (2006) discriminate the ABC and the GCB lymphomas.** This classification can be reproduced in the data set of Dave et al (2006). (A) An ordering of the samples from Hummel et al (2006) by the 1^st^ and 5^th^ principal component (PC1 and PC5, respectively) of the CGSs generated in this data set. (B) An ordering of the samples from Dave at al (2006) using the CGSs and the principal component loadings from (A).
(TIF)

Figure S3 **The results of unsupervised ordering the tumors are robust with respect to the number of gene sets.** Shown are the orderings of tumors in the BL/DLBCL data sets from Hummel et al (2006) and from Dave et al (2006) by the 1^st^ and 2^nd^ PCs of their respective CGSs. In the top, middle and bottom row only the first 40, 30, and 20 CGSs, respectively, were used for computing the PCs.
(TIF)

Figure S4 **Several of the CGSs of the extended DLBCL data set (n = 364) can be grouped into three major components.** Shown is the principal component biplot of the CGSs (grey arrows) and the samples (color circles) based on the PC2 and PC4 of the CGSs. Colors of the circles correspond to the “pathway activation patterns” (PAPs) [6]. The principal components were computed based on the matrix which contains the values of the 50 CGSs for each of the 364 samples. Before this computation, the CGS were scaled to unit variance. The lengths of the arrows represent the standard deviations of the CGSs (all equal to 1), Euclidean distances between the circles represent (up to a scaling factor) the Mahalanobis distances between the samples, and the inner products between the vectors shown as arrows represent the correlations between the CGSs.
(TIF)

Figure S5 **Overall survival in the CAPs and in the corresponding clusters found in the data set of Lenz et al. (2008a).** The three columns show the survival in our extended DLBCL data set, in the CHOP-treated and in the R-CHOP-treated cohort of Lenz et al. (2008a), The three rows represent the results seen in all patients, in the GCB DLBCLs and in the ABC DLBCLs of each cohort. Survival information in our extended DLBCL data set was available for 282 of 364 patients.
(TIF)

Figure S6 **Global distribution of gene expression values of the tumors showing the LoGA profile differs from that of the other lymphomas and is similar to the distribution displayed by the non-malignant GC B cells.** Shown are densities (kernel density estimators) of the VSN-normalized intensities of all genes and of the samples from a given subgroup.
(TIF)

Figure S7 **Distributions of the global expression levels of the LE and of the HE genes in our DLBCL cohort (n = 364) differ from each other in a similar way as in Hebenstreit et al (2011).** Kernel density estimates of the LE and HE genes in all samples from our DLBCL data set. The black curve denotes the sum of the densities corresponding to the LE and the HE genes.
(TIF)

Figure S8 **Distributions of the estimated log fold changes of the LE genes between several groups of samples and the normal GC B cells.** Shown are densities (kernel density estimates) of the distribution of gene-wise generalized log-ratios of the LE genes. Each density corresponds to a comparison between a group of samples and the normal GC B cells. A) Densities corresponding to LoGA and the normal cells. B) Densities corresponding to LoGA and other tumor samples (cf. Figure 5).
(TIF)

Figure S9 **The only difference between this figure and** **Figure 6B** **is that in** **Figure 6B** **the redundantly informative GO terms were left out from the results of the analysis with PAGE while here all significant GO terms are shown.**
(TIF)

Figure S10 **Box plots of genomic complexity, tumor cell content and the Ki67 proliferation index in the CAPs.**
(TIF)

File S1 **Annotation of the probe sets in the 50 CGSs generated in the data set of 364 DLBCL and related mature aggressive B-cell lymphomas other than Burkitt lymphoma.**
(XLSX)

File S2 **Associations between the 50 CGSs and a number of phenotypic characteristics and recurrent genomic aberrations.** Each row corresponds to one CGS. Each column corresponds to one characteristic. A) Values of R-squared (beta statistic) characterizig the association between a CGS and a phenotypic variable. B) Adjusted P-values for the association between a CGS and a phenotypic characteristic. The significant associations are colored.
(XLSX)

File S3 **GO-, KEGG-terms and chromosomal bands enriched in the CGSs.** A GO-term with P-value <0.001 (hypergeometric test) is considered to be significantly enriched. The corresponding significance threshold for a KEGG-term is 0.01 and for a chromosomal band it is 0.001. Listed are significantly enriched terms which consist of more than 10 Entrez IDs. The P-values are not adjusted for multiple testing. In case of no significances empty space is left.
(HTML)

File S4 **Several of the CGSs of the extended DLBCL data set (n = 364) can be grouped into three major components.** Shown is the principal component biplot of the CGSs (black segments) and the samples (color balls) based on the PC1, PC2 and PC4 of the CGSs. Orange balls correspond to the GCB lymphomas and green balls correspond to the ABC lymphomas. Three CGSs: CGS 29 (NUSAP1), CGS 2 (C1QB) and CGS 1 (POSTN) are shown as red segments to visualize the directions of the proliferation, immune response and stromal signatures, respectively.
(MPG)

File S5 **Summary of the characteristics of the 50 CGSs.**
(XLSX)

File S6 **Correlation matrix of the 50 CGSs (Pearson's correlations).**
(XLSX)

File S7 **P-values for enrichment (red) or depletion (blue) of GO BP categories in the subsets of the Venn diagram in** **Figure 6A** **based on the analysis with PAGE.** The shown p-values are the basis of Figure 6B. Columns denote the subsets of the Venn diagram and rows denote the GO BP (Biological Process) categories. Significant enrichment (depletion) (P<0.005, default threshold of PAGE) is shown in red (blue).
(XLSX)

File S8 **Clustering of the 364 patients into the CAPs.**
(XLSX)

Table S1 **Incidence of biologic features in the CAPs.**
(PDF)

Text S1 **Supporting material.**
(PDF)
