# Supplementary figures and images for: Massive Transcriptional Perturbation in Subgroups of Diffuse Large B-Cell Lymphomas
Source: PLoS One. 2013 Nov 4;8(11):e76287. doi: 10.1371/journal.pone.0076287 (PMC3817189; doi:10.1371/journal.pone.0076287)

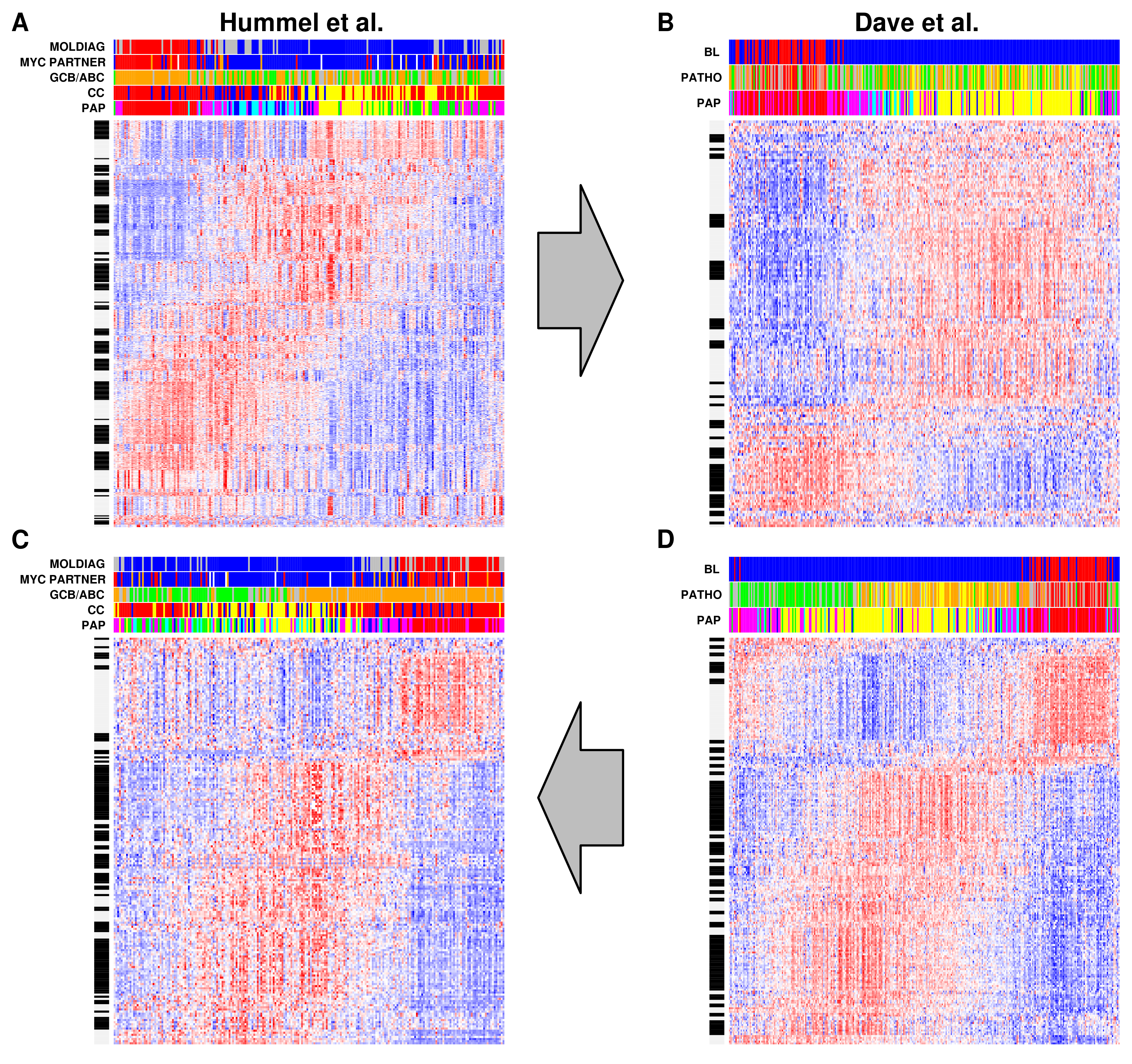

Supplement: Figure S1 — CGSs discriminate BL/DLBCLs according to several previously reported molecular classifications. This result is reproducible across different BL/DLBCL data sets. Heat maps (A) and (D) show expression of the 50 CGSs generated in the BL/DLBCL data set of Hummel et al (2006) and Dave et al (2006), respectively. Heat maps (B) and (C) show the CGSs from the heat maps (A) and (D), respectively, mapped to the other data set. Samples (columns) and gene sets (rows) are arranged in the angular order of their projections onto the plane spanned by the first and the second principal axes (Text S1). This plane is determined in the data set where the CGSs were created and is used to order the samples in the original data set and in the other data set. (TIF) [file pone.0076287.s001.tif]

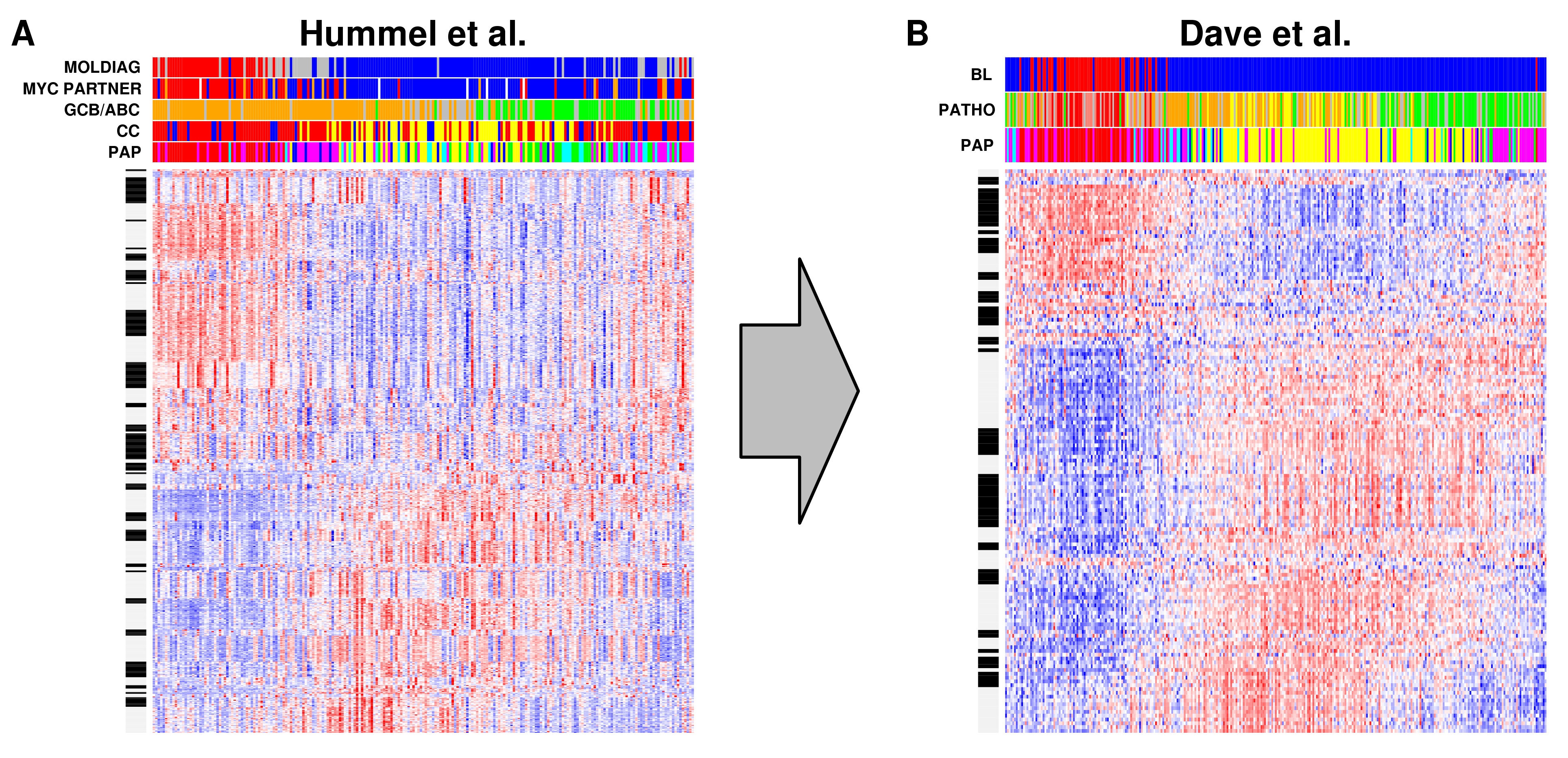

Supplement: Figure S2 — The CGSs generated in the BL/DLBCL data set of Hummel et al (2006) discriminate the ABC and the GCB lymphomas. This classification can be reproduced in the data set of Dave et al (2006). (A) An ordering of the samples from Hummel et al (2006) by the 1st and 5th principal component (PC1 and PC5, respectively) of the CGSs generated in this data set. (B) An ordering of the samples from Dave at al (2006) using the CGSs and the principal component loadings from (A). (TIF) [file pone.0076287.s002.tif]

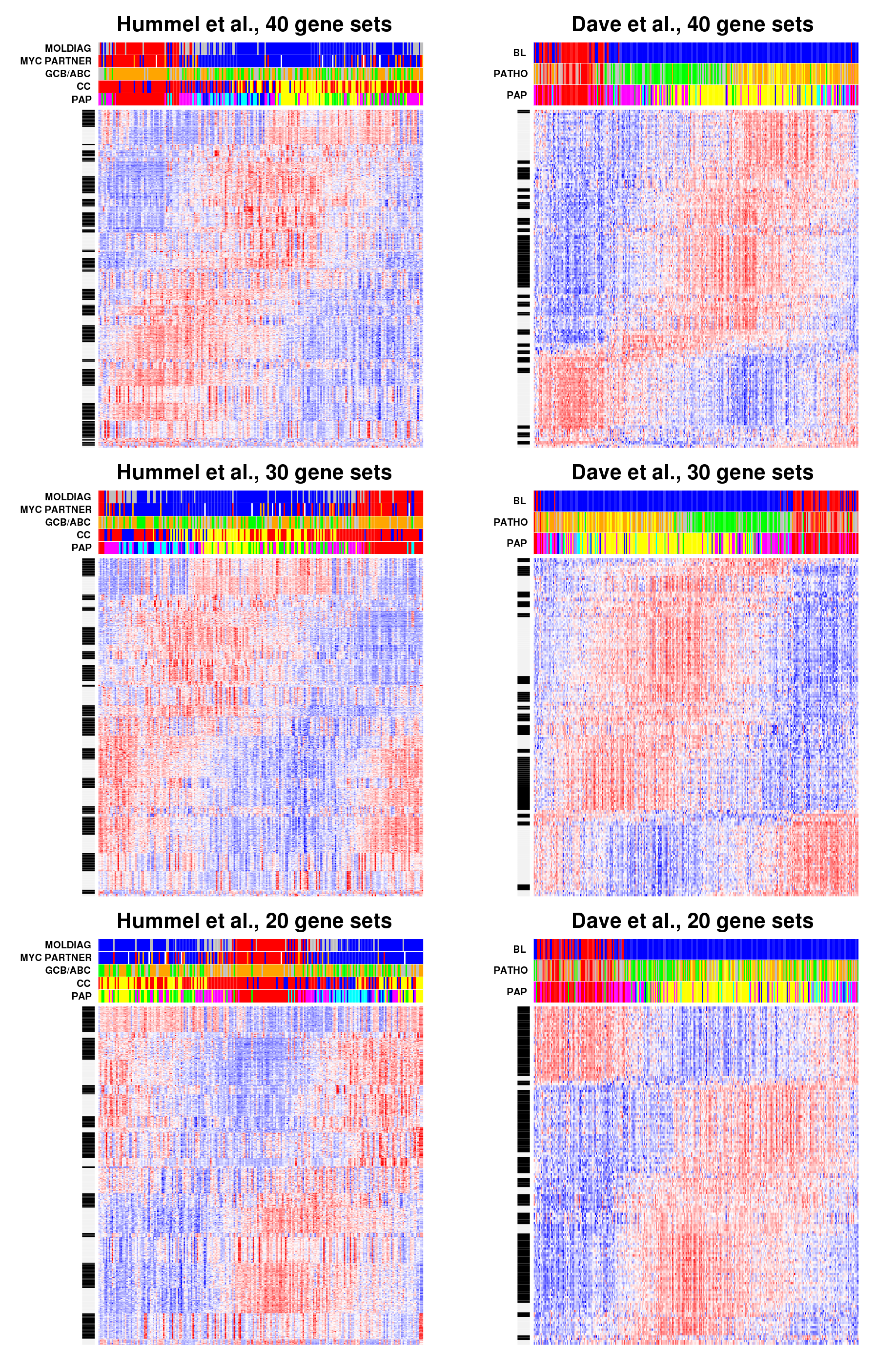

Supplement: Figure S3 — The results of unsupervised ordering the tumors are robust with respect to the number of gene sets. Shown are the orderings of tumors in the BL/DLBCL data sets from Hummel et al (2006) and from Dave et al (2006) by the 1st and 2nd PCs of their respective CGSs. In the top, middle and bottom row only the first 40, 30, and 20 CGSs, respectively, were used for computing the PCs. (TIF) [file pone.0076287.s003.tif]

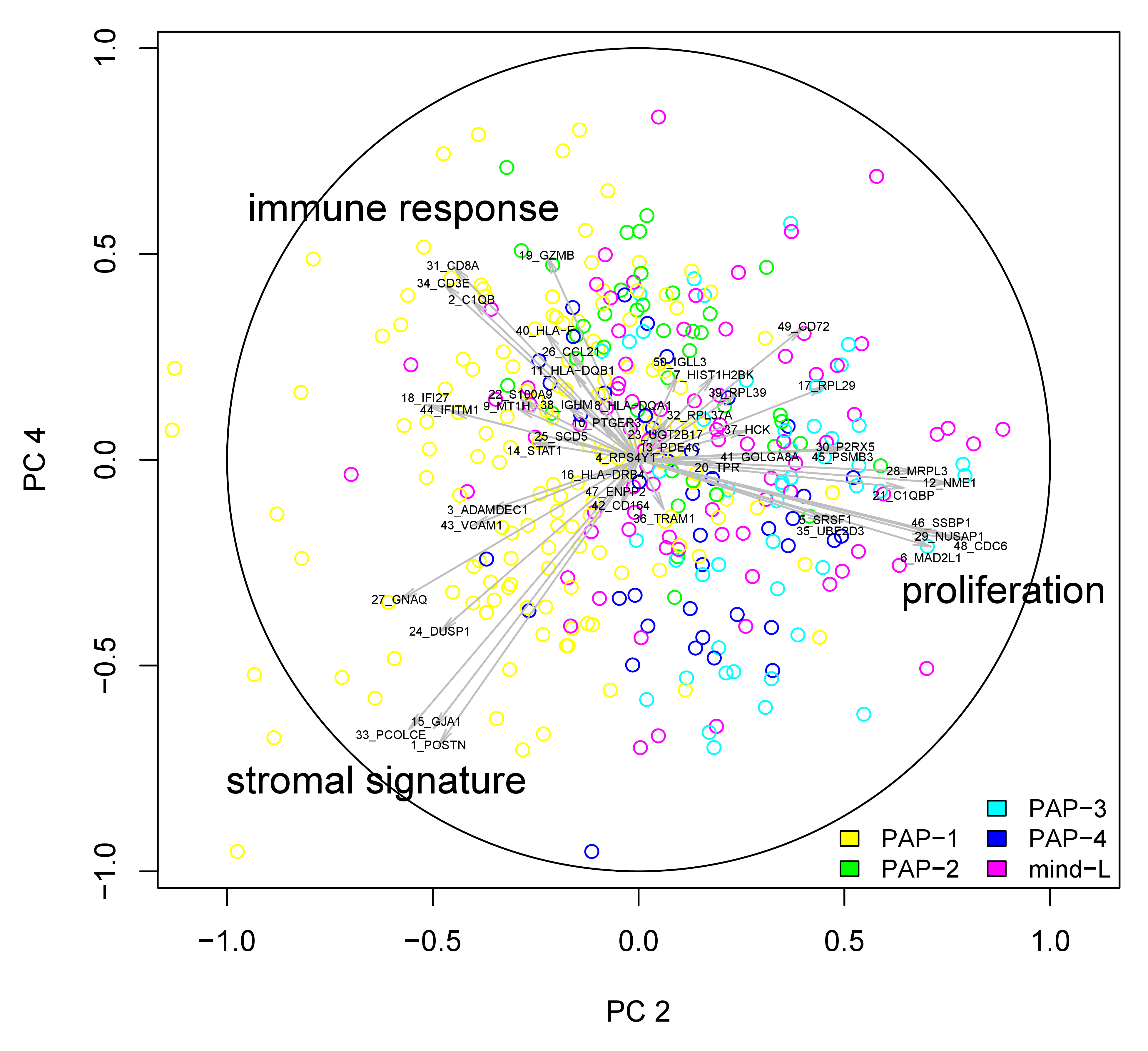

Supplement: Figure S4 — Several of the CGSs of the extended DLBCL data set (n = 364) can be grouped into three major components. Shown is the principal component biplot of the CGSs (grey arrows) and the samples (color circles) based on the PC2 and PC4 of the CGSs. Colors of the circles correspond to the “pathway activation patterns” (PAPs) [6]. The principal components were computed based on the matrix which contains the values of the 50 CGSs for each of the 364 samples. Before this computation, the CGS were scaled to unit variance. The lengths of the arrows represent the standard deviations of the CGSs (all equal to 1), Euclidean distances between the circles represent (up to a scaling factor) the Mahalanobis distances between the samples, and the inner products between the vectors shown as arrows represent the correlations between the CGSs. (TIF) [file pone.0076287.s004.tif]

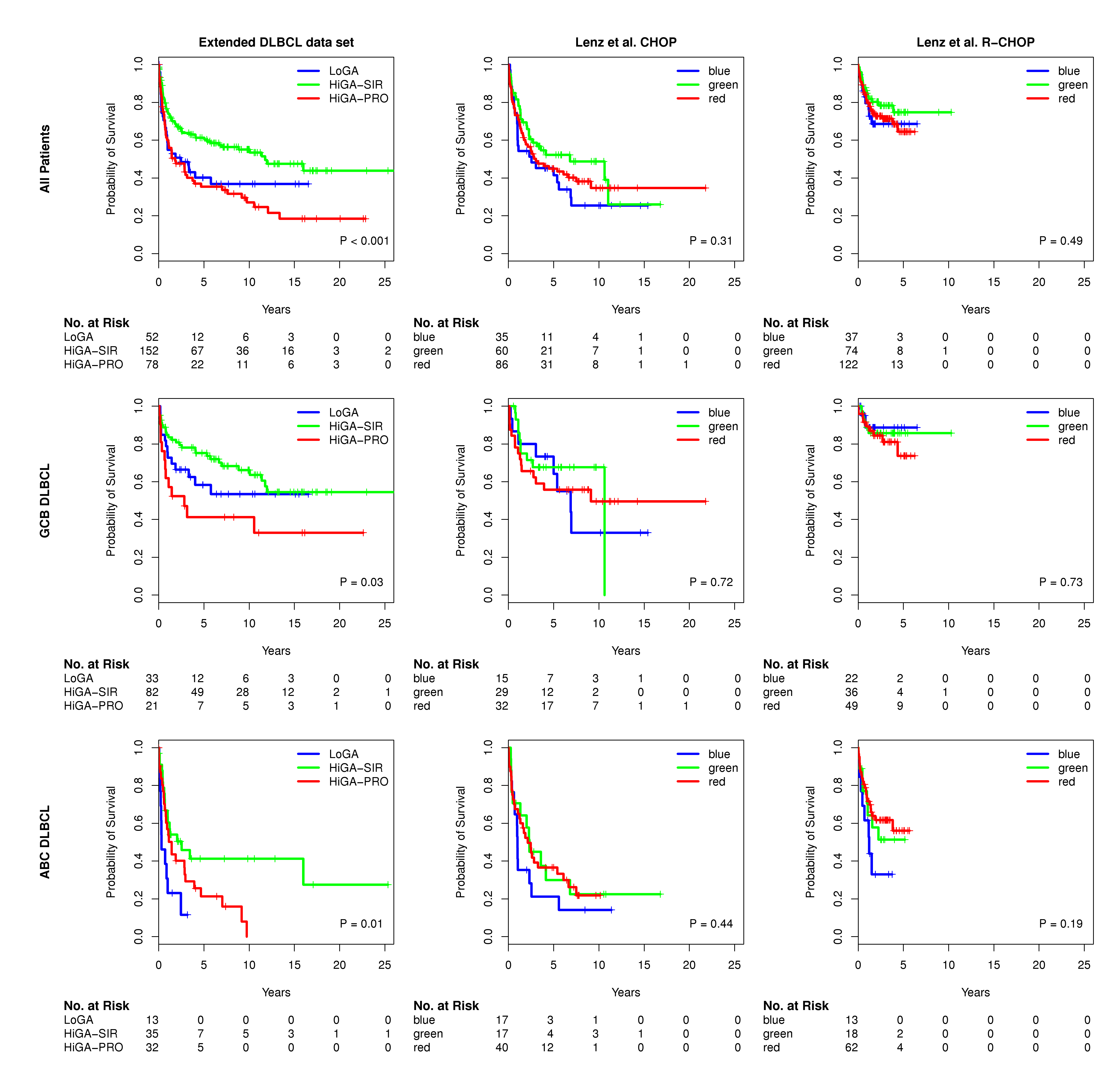

Supplement: Figure S5 — Overall survival in the CAPs and in the corresponding clusters found in the data set of Lenz et al. (2008a). The three columns show the survival in our extended DLBCL data set, in the CHOP-treated and in the R-CHOP-treated cohort of Lenz et al. (2008a), The three rows represent the results seen in all patients, in the GCB DLBCLs and in the ABC DLBCLs of each cohort. Survival information in our extended DLBCL data set was available for 282 of 364 patients. (TIF) [file pone.0076287.s005.tif]

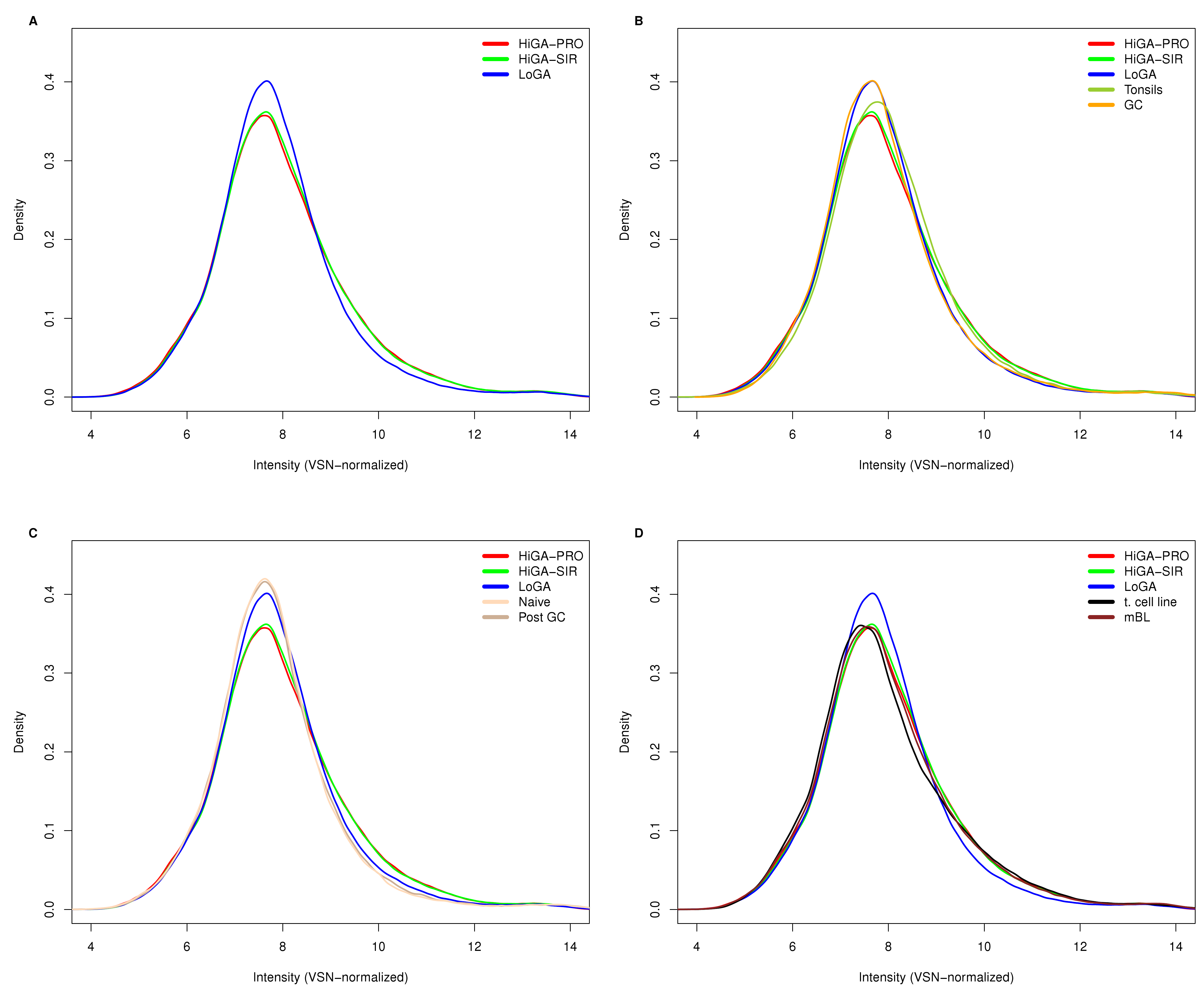

Supplement: Figure S6 — Global distribution of gene expression values of the tumors showing the LoGA profile differs from that of the other lymphomas and is similar to the distribution displayed by the non-malignant GC B cells. Shown are densities (kernel density estimators) of the VSN-normalized intensities of all genes and of the samples from a given subgroup. (TIF) [file pone.0076287.s006.tif]

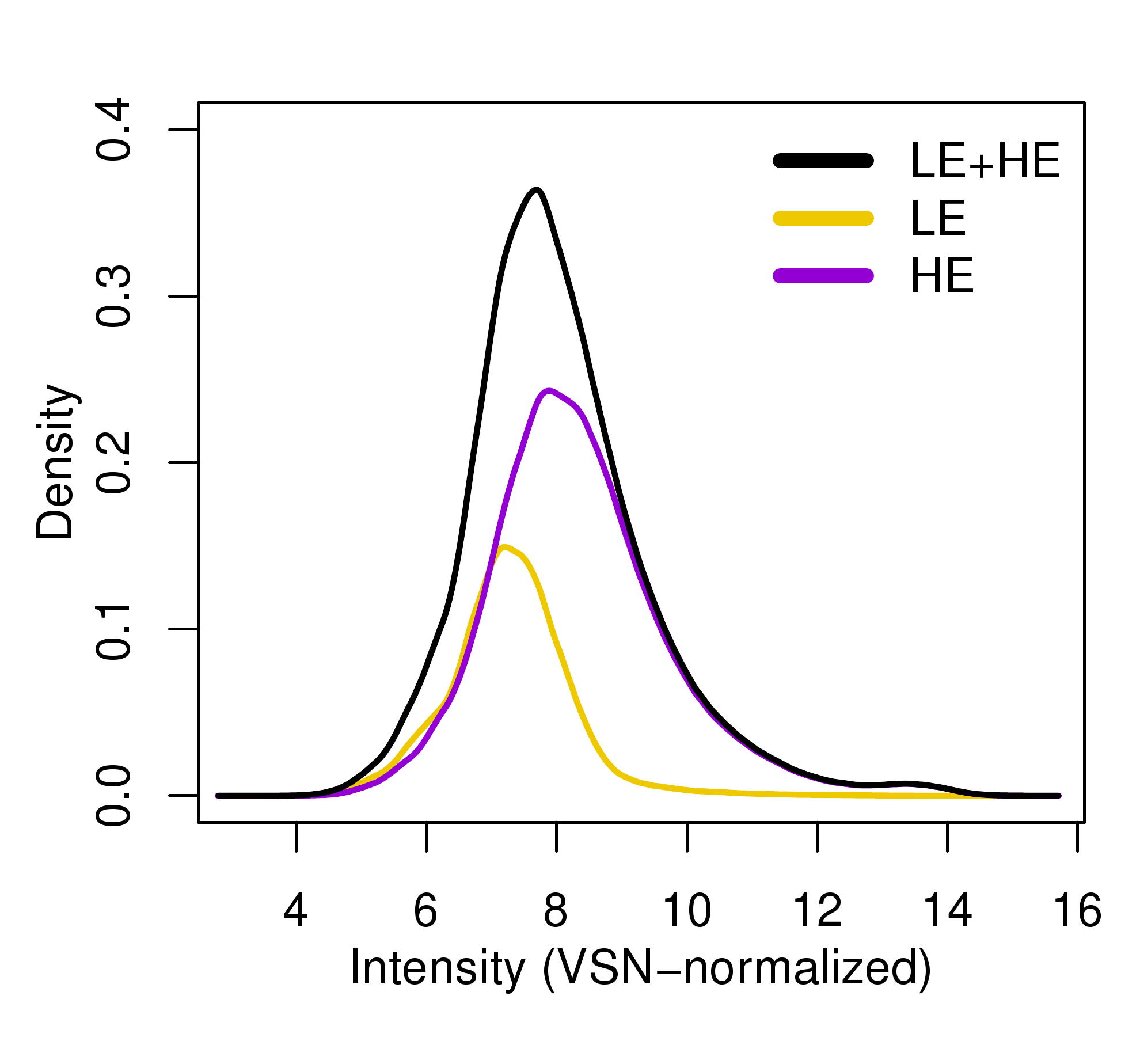

Supplement: Figure S7 — Distributions of the global expression levels of the LE and of the HE genes in our DLBCL cohort (n = 364) differ from each other in a similar way as in Hebenstreit et al (2011). Kernel density estimates of the LE and HE genes in all samples from our DLBCL data set. The black curve denotes the sum of the densities corresponding to the LE and the HE genes. (TIF) [file pone.0076287.s007.tif]

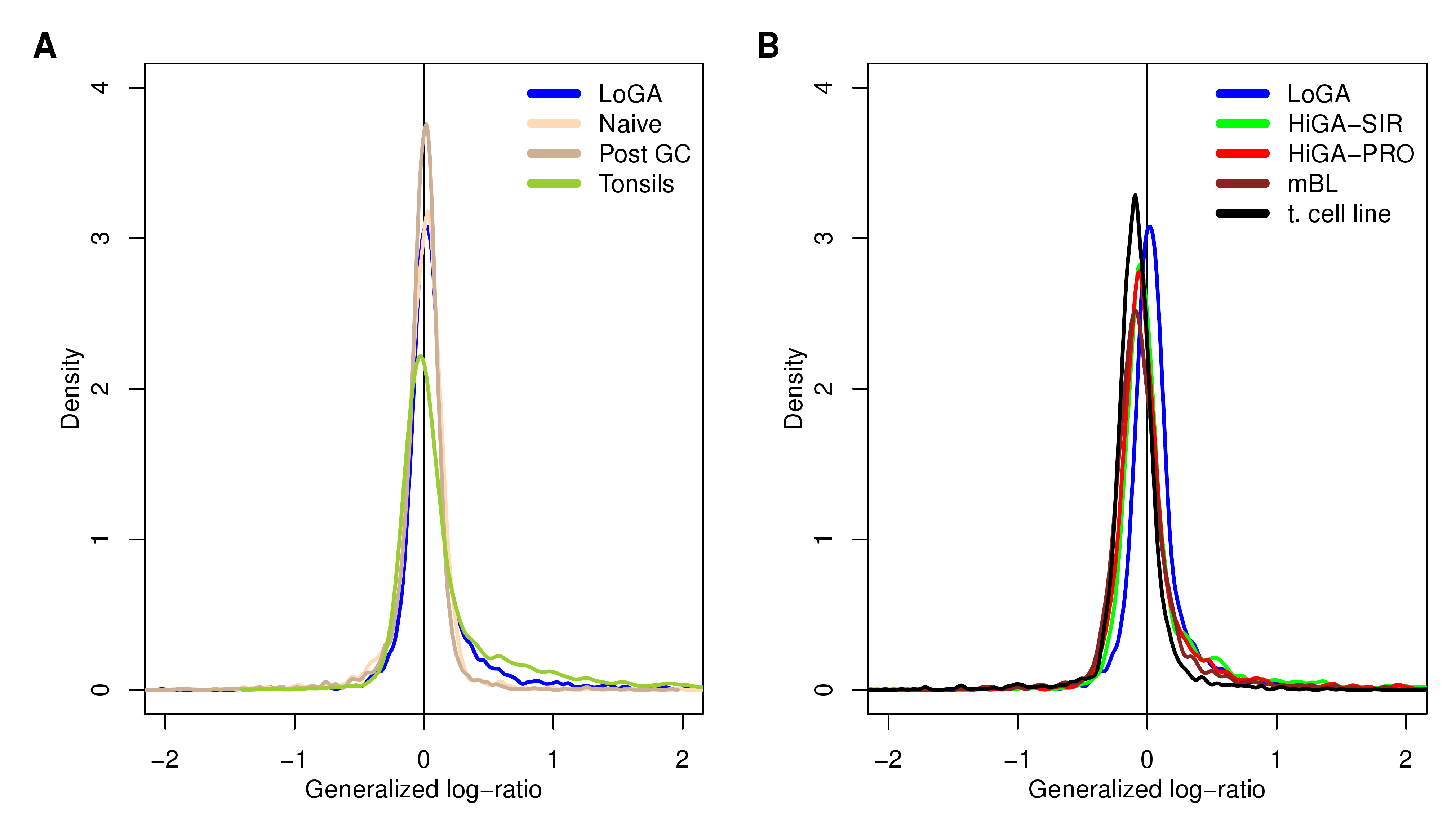

Supplement: Figure S8 — Distributions of the estimated log fold changes of the LE genes between several groups of samples and the normal GC B cells. Shown are densities (kernel density estimates) of the distribution of gene-wise generalized log-ratios of the LE genes. Each density corresponds to a comparison between a group of samples and the normal GC B cells. A) Densities corresponding to LoGA and the normal cells. B) Densities corresponding to LoGA and other tumor samples (cf. Figure 5). (TIF) [file pone.0076287.s008.tif]

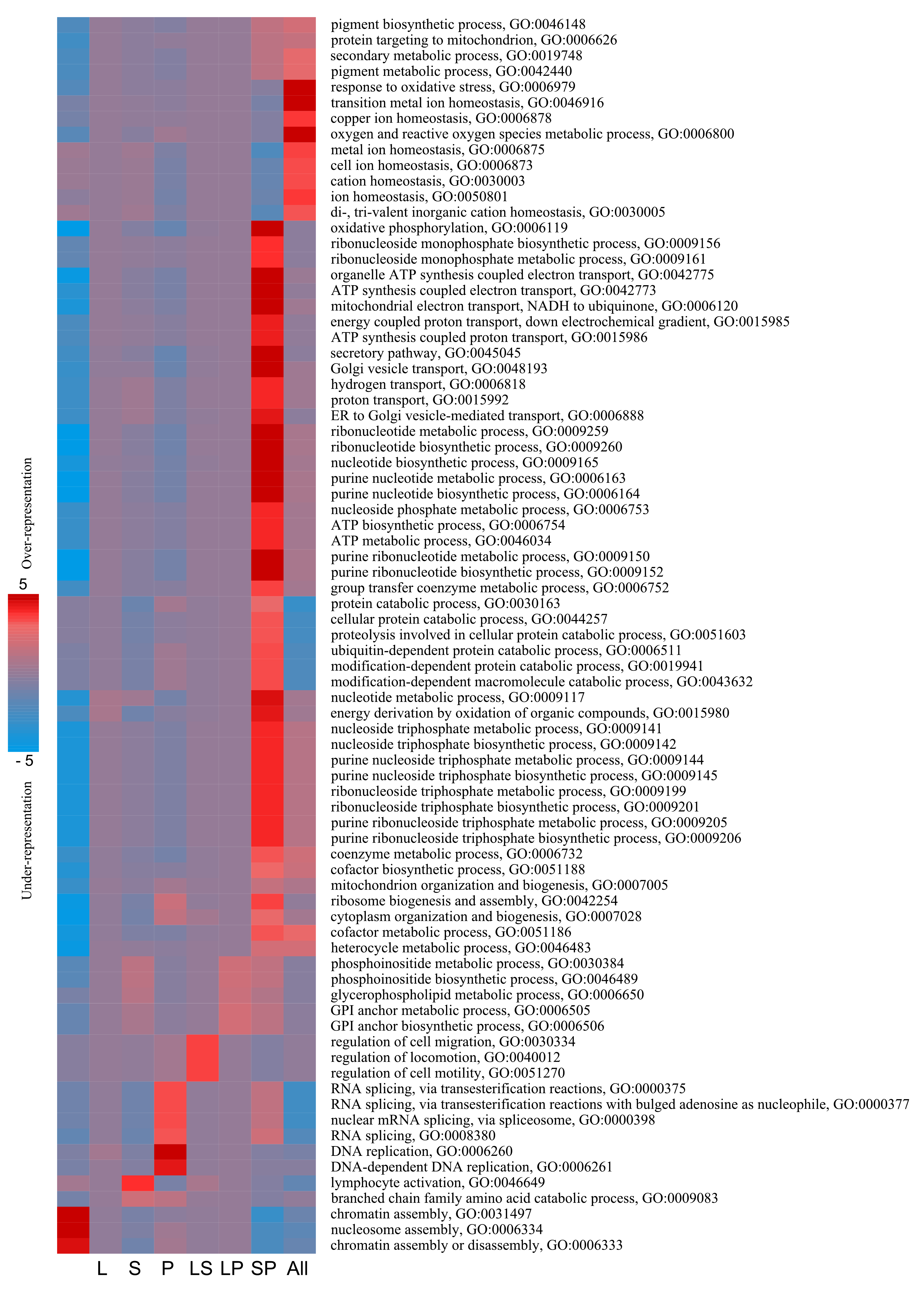

Supplement: Figure S9 — The only difference between this figure and Figure 6B is that in Figure 6B the redundantly informative GO terms were left out from the results of the analysis with PAGE while here all significant GO terms are shown. (TIF) [file pone.0076287.s009.tif]

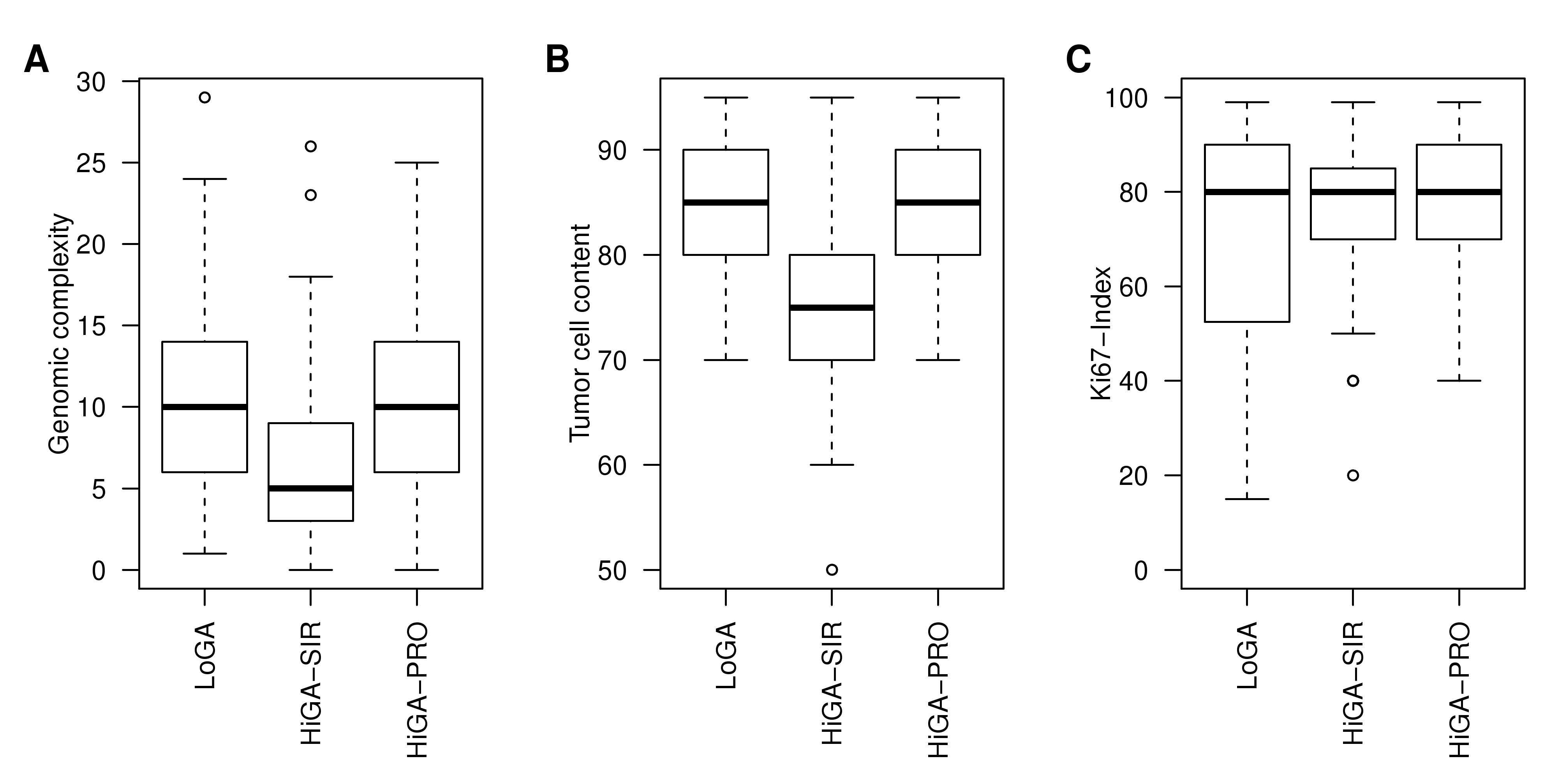

Supplement: Figure S10 — Box plots of genomic complexity, tumor cell content and the Ki67 proliferation index in the CAPs. (TIF) [file pone.0076287.s010.tif]
