# Supplementary material for: Massive Transcriptional Perturbation in Subgroups of Diffuse Large B-Cell Lymphomas
Source: PLoS One. 2013 Nov 4;8(11):e76287. doi: 10.1371/journal.pone.0076287 (PMC3817189; doi:10.1371/journal.pone.0076287)
Supplement: File S3 — GO-, KEGG-terms and chromosomal bands enriched in the CGSs. A GO-term with P-value <0.001 (hypergeometric test) is considered to be significantly enriched. The corresponding significance threshold for a KEGG-term is 0.01 and for a chromosomal band it is 0.001. Listed are significantly enriched terms which consist of more than 10 Entrez IDs. The P-values are not adjusted for multiple testing. In case of no significances empty space is left. (HTML) [file pone.0076287.s013.html]

# File S3. GO-, KEGG-terms and chromosomal bands enriched in the CGSs.

A GO-term with P-value < 0.001 (hypergeometric test) is considered to be significantly enriched.
The corresponding significance threshold for a KEGG-term is 0.01 and for a chromosomal band it is 0.001.   
Listed are significantly enriched terms which consist of more than 10 Entrez IDs.
The P-values are not adjusted for multiple testing. In case of no significances empty space is left.  
  

# CGS 1 POSTN n= 28


Gene to GO BP test for over-representation

| GOBPID | Pvalue | OddsRatio | ExpCount | Count | Size | Term |
| GO:0030198 | 0.0000000000 | 79.8713450292 | 0 | 10 | 67 | extracellular matrix organization |
| GO:0030199 | 0.0000000000 | 222.7685185185 | 0 | 7 | 19 | collagen fibril organization |
| GO:0043062 | 0.0000000000 | 52.7131782946 | 0 | 10 | 96 | extracellular structure organization |
| GO:0043588 | 0.0000000061 | 114.5166666667 | 0 | 5 | 20 | skin development |
| GO:0007155 | 0.0000000082 | 14.1036552100 | 2 | 12 | 435 | cell adhesion |
| GO:0022610 | 0.0000000082 | 14.1036552100 | 2 | 12 | 435 | biological adhesion |
| GO:0001501 | 0.0000000177 | 24.2658284688 | 1 | 8 | 139 | skeletal system development |
| GO:0001568 | 0.0000000239 | 18.8043750000 | 1 | 9 | 209 | blood vessel development |
| GO:0001944 | 0.0000000294 | 18.3320121951 | 1 | 9 | 214 | vasculature development |
| GO:0032963 | 0.0000000543 | 68.6100000000 | 0 | 5 | 30 | collagen metabolic process |
| GO:0044259 | 0.0000000764 | 63.5092592593 | 0 | 5 | 32 | multicellular organismal macromolecule metabolic process |
| GO:0032964 | 0.0000000932 | 145.5449735450 | 0 | 4 | 13 | collagen biosynthetic process |
| GO:0048731 | 0.0000001065 | 9.2182170543 | 5 | 17 | 1307 | system development |
| GO:0007275 | 0.0000001653 | 9.0320164763 | 6 | 18 | 1544 | multicellular organismal development |
| GO:0044236 | 0.0000002148 | 50.3823529412 | 0 | 5 | 39 | multicellular organismal metabolic process |
| GO:0032501 | 0.0000002280 | 9.6898608350 | 7 | 20 | 2032 | multicellular organismal process |
| GO:0018149 | 0.0000002354 | 109.1111111111 | 0 | 4 | 16 | peptide cross-linking |
| GO:0048513 | 0.0000002536 | 8.7267326733 | 4 | 15 | 1025 | organ development |
| GO:0048856 | 0.0000003585 | 8.3419170243 | 5 | 17 | 1415 | anatomical structure development |
| GO:0008544 | 0.0000006392 | 25.2668730650 | 0 | 6 | 91 | epidermis development |
| GO:0032502 | 0.0000007716 | 7.9620973910 | 6 | 18 | 1699 | developmental process |
| GO:0007398 | 0.0000009342 | 23.5801041064 | 0 | 6 | 97 | ectoderm development |
| GO:0009887 | 0.0000010554 | 11.6950158228 | 1 | 9 | 325 | organ morphogenesis |
| GO:0016477 | 0.0000044443 | 11.2278615417 | 1 | 8 | 285 | cell migration |
| GO:0048870 | 0.0000054618 | 10.8994840041 | 1 | 8 | 293 | cell motility |
| GO:0051674 | 0.0000054618 | 10.8994840041 | 1 | 8 | 293 | localization of cell |
| GO:0009653 | 0.0000090693 | 7.3380523381 | 2 | 11 | 677 | anatomical structure morphogenesis |
| GO:0031589 | 0.0000129769 | 20.4909638554 | 0 | 5 | 88 | cell-substrate adhesion |
| GO:0040011 | 0.0000279813 | 8.5810055866 | 1 | 8 | 366 | locomotion |
| GO:0009611 | 0.0000346633 | 8.3111748765 | 1 | 8 | 377 | response to wounding |
| GO:0006928 | 0.0000374049 | 8.2170004731 | 1 | 8 | 381 | cellular component movement |
| GO:0006029 | 0.0000532856 | 52.0303030303 | 0 | 3 | 21 | proteoglycan metabolic process |
| GO:0042060 | 0.0000766020 | 13.8606557377 | 0 | 5 | 127 | wound healing |
| GO:0009888 | 0.0000845006 | 7.2632317844 | 2 | 8 | 427 | tissue development |
| GO:0042476 | 0.0002759913 | 28.3181818182 | 0 | 3 | 36 | odontogenesis |
| GO:0001503 | 0.0004293554 | 13.3313696613 | 0 | 4 | 101 | ossification |
| GO:0030204 | 0.0005554310 | 74.7608695652 | 0 | 2 | 10 | chondroitin sulfate metabolic process |
| GO:0060323 | 0.0005554310 | 74.7608695652 | 0 | 2 | 10 | head morphogenesis |
| GO:0070206 | 0.0005554310 | 74.7608695652 | 0 | 2 | 10 | protein trimerization |
| GO:0010171 | 0.0008110255 | 59.7913043478 | 0 | 2 | 12 | body morphogenesis |
| GO:0050654 | 0.0009563611 | 54.3478260870 | 0 | 2 | 13 | chondroitin sulfate proteoglycan metabolic process |

  


Gene to GO CC test for over-representation

| GOCCID | Pvalue | OddsRatio | ExpCount | Count | Size | Term |
| GO:0005578 | 0.0000000000 | 113.2337448560 | 1 | 19 | 154 | proteinaceous extracellular matrix |
| GO:0031012 | 0.0000000000 | 92.2619528620 | 1 | 19 | 184 | extracellular matrix |
| GO:0044421 | 0.0000000000 | 56.1681415929 | 2 | 22 | 474 | extracellular region part |
| GO:0005576 | 0.0000000000 | 53.4040268456 | 3 | 24 | 769 | extracellular region |
| GO:0044420 | 0.0000000000 | 103.6273584906 | 0 | 12 | 65 | extracellular matrix part |
| GO:0005581 | 0.0000000000 | 173.1529411765 | 0 | 8 | 25 | collagen |
| GO:0005615 | 0.0000000000 | 26.8462273161 | 1 | 16 | 365 | extracellular space |
| GO:0005604 | 0.0000136207 | 33.9814814815 | 0 | 4 | 40 | basement membrane |

  


Gene to GO MF test for over-representation

| GOMFID | Pvalue | OddsRatio | ExpCount | Count | Size | Term |
| GO:0005201 | 0.0000000000 | 151.4305019305 | 0 | 11 | 48 | extracellular matrix structural constituent |
| GO:0005539 | 0.0000000000 | 57.8722826087 | 0 | 9 | 78 | glycosaminoglycan binding |
| GO:0001871 | 0.0000000000 | 51.8011363636 | 0 | 9 | 86 | pattern binding |
| GO:0030247 | 0.0000000000 | 51.8011363636 | 0 | 9 | 86 | polysaccharide binding |
| GO:0005198 | 0.0000000000 | 21.9591988131 | 1 | 13 | 350 | structural molecule activity |
| GO:0030246 | 0.0000000055 | 22.4775000000 | 1 | 9 | 184 | carbohydrate binding |
| GO:0008201 | 0.0000000452 | 40.8401913876 | 0 | 6 | 61 | heparin binding |
| GO:0019838 | 0.0000039699 | 26.4962686567 | 0 | 5 | 72 | growth factor binding |
| GO:0005518 | 0.0001026883 | 40.5909090909 | 0 | 3 | 27 | collagen binding |
| GO:0046332 | 0.0001889251 | 32.4454545455 | 0 | 3 | 33 | SMAD binding |
| GO:0005178 | 0.0004176410 | 24.3000000000 | 0 | 3 | 43 | integrin binding |

  


Gene to KEGG test for over-representation

| KEGGID | Pvalue | OddsRatio | ExpCount | Count | Size | Term |
| 04512 | 0.0000000000 | 150.2500000000 | 0 | 10 | 50 | ECM-receptor interaction |
| 04510 | 0.0000000001 | 45.2093023256 | 1 | 10 | 139 | Focal adhesion |
| 05146 | 0.0000000006 | 49.2919254658 | 0 | 8 | 77 | Amoebiasis |
| 05222 | 0.0034687482 | 12.0282258065 | 0 | 3 | 65 | Small cell lung cancer |

  
  
  

# CGS 2 C1QB n= 29


Gene to GO BP test for over-representation

| GOBPID | Pvalue | OddsRatio | ExpCount | Count | Size | Term |
| GO:0006954 | 0.0000000000 | 27.2312775330 | 1 | 13 | 240 | inflammatory response |
| GO:0006952 | 0.0000000000 | 19.6359223301 | 2 | 15 | 427 | defense response |
| GO:0009611 | 0.0000000000 | 19.3460478915 | 1 | 14 | 377 | response to wounding |
| GO:0006955 | 0.0000000000 | 17.5793216630 | 2 | 15 | 472 | immune response |
| GO:0002376 | 0.0000000078 | 11.0253209700 | 3 | 15 | 716 | immune system process |
| GO:0006956 | 0.0000000155 | 91.8048128342 | 0 | 5 | 22 | complement activation |
| GO:0002455 | 0.0000000197 | 86.6919191919 | 0 | 5 | 23 | humoral immune response mediated by circulating immunoglobulin |
| GO:0002541 | 0.0000000249 | 82.1172248804 | 0 | 5 | 24 | activation of plasma proteins involved in acute inflammatory response |
| GO:0006959 | 0.0000000693 | 37.5384615385 | 0 | 6 | 58 | humoral immune response |
| GO:0002526 | 0.0000001263 | 33.6256157635 | 0 | 6 | 64 | acute inflammatory response |
| GO:0050778 | 0.0000001459 | 23.0422330097 | 0 | 7 | 110 | positive regulation of immune response |
| GO:0006958 | 0.0000002450 | 108.6640316206 | 0 | 4 | 15 | complement activation, classical pathway |
| GO:0045087 | 0.0000004139 | 19.5623966942 | 1 | 7 | 128 | innate immune response |
| GO:0002684 | 0.0000004330 | 15.3318077803 | 1 | 8 | 192 | positive regulation of immune system process |
| GO:0048584 | 0.0000004330 | 15.3318077803 | 1 | 8 | 192 | positive regulation of response to stimulus |
| GO:0002253 | 0.0000004501 | 26.6575342466 | 0 | 6 | 79 | activation of immune response |
| GO:0050896 | 0.0000005344 | 7.6890080429 | 7 | 20 | 1885 | response to stimulus |
| GO:0051605 | 0.0000007563 | 37.9323725055 | 0 | 5 | 46 | protein maturation by peptide bond cleavage |
| GO:0006950 | 0.0000007926 | 7.2524901186 | 5 | 16 | 1166 | response to stress |
| GO:0016064 | 0.0000015542 | 32.3674242424 | 0 | 5 | 53 | immunoglobulin mediated immune response |
| GO:0019724 | 0.0000015542 | 32.3674242424 | 0 | 5 | 53 | B cell mediated immunity |
| GO:0016485 | 0.0000026684 | 28.7457912458 | 0 | 5 | 59 | protein processing |
| GO:0050776 | 0.0000030534 | 14.2524242424 | 1 | 7 | 172 | regulation of immune response |
| GO:0051604 | 0.0000050389 | 25.0073313783 | 0 | 5 | 67 | protein maturation |
| GO:0002682 | 0.0000094064 | 9.8939876381 | 1 | 8 | 289 | regulation of immune system process |
| GO:0002449 | 0.0000136779 | 20.0914994097 | 0 | 5 | 82 | lymphocyte mediated immunity |
| GO:0002460 | 0.0000136779 | 20.0914994097 | 0 | 5 | 82 | adaptive immune response based on somatic recombination of immune receptors built from immunoglobulin superfamily domains |
| GO:0002250 | 0.0000145159 | 19.8310023310 | 0 | 5 | 83 | adaptive immune response |
| GO:0002252 | 0.0000240191 | 12.8266666667 | 1 | 6 | 156 | immune effector process |
| GO:0002443 | 0.0000310387 | 16.7786561265 | 0 | 5 | 97 | leukocyte mediated immunity |
| GO:0048583 | 0.0000437647 | 7.8841803650 | 1 | 8 | 357 | regulation of response to stimulus |
| GO:0002920 | 0.0006488511 | 68.7600000000 | 0 | 2 | 10 | regulation of humoral immune response |

  


Gene to GO CC test for over-representation

| GOCCID | Pvalue | OddsRatio | ExpCount | Count | Size | Term |
| GO:0005576 | 0.0000000835 | 9.4455425369 | 3 | 14 | 769 | extracellular region |
| GO:0005615 | 0.0000313877 | 8.2795223352 | 1 | 8 | 365 | extracellular space |
| GO:0005886 | 0.0000586666 | 4.9180602007 | 6 | 15 | 1510 | plasma membrane |
| GO:0044421 | 0.0001995521 | 6.2444093065 | 2 | 8 | 474 | extracellular region part |
| GO:0044425 | 0.0002014871 | 4.3213367609 | 9 | 18 | 2352 | membrane part |
| GO:0031224 | 0.0002233794 | 4.2318254083 | 7 | 16 | 1903 | intrinsic to membrane |
| GO:0016021 | 0.0007545692 | 3.7129978471 | 7 | 15 | 1873 | integral to membrane |

  


Gene to GO MF test for over-representation

| GOMFID | Pvalue | OddsRatio | ExpCount | Count | Size | Term |
| GO:0019865 | 0.0000000551 | 170.4761904762 | 0 | 4 | 12 | immunoglobulin binding |
| GO:0004871 | 0.0000003354 | 9.1747027741 | 3 | 13 | 770 | signal transducer activity |
| GO:0060089 | 0.0000003354 | 9.1747027741 | 3 | 13 | 770 | molecular transducer activity |
| GO:0004872 | 0.0000003659 | 10.5008588606 | 2 | 11 | 510 | receptor activity |
| GO:0032403 | 0.0002127891 | 11.0204402516 | 1 | 5 | 164 | protein complex binding |

  


Gene to KEGG test for over-representation

| KEGGID | Pvalue | OddsRatio | ExpCount | Count | Size | Term |
| 04610 | 0.0000003619 | 52.3263888889 | 0 | 5 | 37 | Complement and coagulation cascades |
| 05322 | 0.0001582874 | 19.5737704918 | 0 | 4 | 65 | Systemic lupus erythematosus |
| 04620 | 0.0033430528 | 12.3140495868 | 0 | 3 | 69 | Toll-like receptor signaling pathway |
| 05020 | 0.0054922555 | 21.9057971014 | 0 | 2 | 25 | Prion diseases |

  


Gene to ChrMap test for over-representation

| ChrMapID | Pvalue | OddsRatio | ExpCount | Count | Size |
| 1p36.12 | 0.0006526845 | 67.1028806584 | 0 | 2 | 11 |

  
  

# CGS 3 ADAMDEC1 n= 30

  


Gene to GO CC test for over-representation

| GOCCID | Pvalue | OddsRatio | ExpCount | Count | Size | Term |
| GO:0042470 | 0.0002564324 | 15.1646753247 | 0 | 4 | 81 | melanosome |
| GO:0048770 | 0.0002564324 | 15.1646753247 | 0 | 4 | 81 | pigment granule |

  
  
  


Gene to ChrMap test for over-representation

| ChrMapID | Pvalue | OddsRatio | ExpCount | Count | Size |
| 7q36.1 | 0.0008366428 | 58.2214285714 | 0 | 2 | 12 |

  
  

# CGS 4 RPS4Y1 n= 6

  
  
  
  


Gene to ChrMap test for over-representation

| ChrMapID | Pvalue | OddsRatio | ExpCount | Count | Size |
| Y | 0.0000000000 | Inf | 0 | 6 | 21 |

  
  

# CGS 5 SRSF1 n= 30

  


Gene to GO CC test for over-representation

| GOCCID | Pvalue | OddsRatio | ExpCount | Count | Size | Term |
| GO:0044428 | 0.0000136143 | 5.6937123494 | 5 | 15 | 1343 | nuclear part |
| GO:0031981 | 0.0000274452 | 5.6265208476 | 4 | 13 | 1058 | nuclear lumen |
| GO:0005634 | 0.0000504575 | 5.4588827203 | 11 | 21 | 2903 | nucleus |
| GO:0070013 | 0.0002125146 | 4.4736931050 | 5 | 13 | 1281 | intracellular organelle lumen |
| GO:0043233 | 0.0002536785 | 4.3815614618 | 5 | 13 | 1303 | organelle lumen |
| GO:0031974 | 0.0003111697 | 4.2766500217 | 5 | 13 | 1329 | membrane-enclosed lumen |
| GO:0005730 | 0.0003530331 | 5.7053877297 | 2 | 8 | 515 | nucleolus |

  
  
  
  
  

# CGS 6 MAD2L1 n= 30


Gene to GO BP test for over-representation

| GOBPID | Pvalue | OddsRatio | ExpCount | Count | Size | Term |
| GO:0022402 | 0.0000002120 | 9.4598969831 | 2 | 12 | 465 | cell cycle process |
| GO:0022403 | 0.0000013459 | 9.4724637681 | 2 | 10 | 355 | cell cycle phase |
| GO:0007059 | 0.0000108006 | 20.9723076923 | 0 | 5 | 70 | chromosome segregation |
| GO:0000279 | 0.0000128185 | 9.2601398601 | 1 | 8 | 268 | M phase |
| GO:0016071 | 0.0000135359 | 9.1866759195 | 1 | 8 | 270 | mRNA metabolic process |
| GO:0007049 | 0.0000141576 | 6.0993117011 | 3 | 12 | 690 | cell cycle |
| GO:0000278 | 0.0000144112 | 7.9255362202 | 2 | 9 | 362 | mitotic cell cycle |
| GO:0000280 | 0.0000155085 | 10.7761674718 | 1 | 7 | 196 | nuclear division |
| GO:0007067 | 0.0000155085 | 10.7761674718 | 1 | 7 | 196 | mitosis |
| GO:0006996 | 0.0000156188 | 5.4694415174 | 4 | 14 | 963 | organelle organization |
| GO:0048285 | 0.0000182766 | 10.4905871806 | 1 | 7 | 201 | organelle fission |
| GO:0000087 | 0.0000194930 | 10.3804347826 | 1 | 7 | 203 | M phase of mitotic cell cycle |
| GO:0006397 | 0.0000401332 | 9.2148221344 | 1 | 7 | 227 | mRNA processing |
| GO:0051276 | 0.0000840775 | 6.9957219251 | 2 | 8 | 348 | chromosome organization |
| GO:0010564 | 0.0001349984 | 11.9787610619 | 1 | 5 | 118 | regulation of cell cycle process |
| GO:0006323 | 0.0001438780 | 17.7887874837 | 0 | 4 | 63 | DNA packaging |
| GO:0006396 | 0.0002862482 | 5.7842305115 | 2 | 8 | 415 | RNA processing |
| GO:0000070 | 0.0003363518 | 26.2528735632 | 0 | 3 | 32 | mitotic sister chromatid segregation |
| GO:0000819 | 0.0003363518 | 26.2528735632 | 0 | 3 | 32 | sister chromatid segregation |
| GO:0071103 | 0.0003989579 | 13.4181459566 | 0 | 4 | 82 | DNA conformation change |
| GO:0008380 | 0.0004237159 | 7.2949561404 | 1 | 6 | 234 | RNA splicing |
| GO:0051301 | 0.0004744519 | 7.1330472103 | 1 | 6 | 239 | cell division |
| GO:0016070 | 0.0008120219 | 3.5586340206 | 6 | 14 | 1372 | RNA metabolic process |

  


Gene to GO CC test for over-representation

| GOCCID | Pvalue | OddsRatio | ExpCount | Count | Size | Term |
| GO:0005634 | 0.0000000006 | 21.9081739130 | 12 | 28 | 2903 | nucleus |
| GO:0044428 | 0.0000024747 | 5.9644970414 | 5 | 17 | 1343 | nuclear part |
| GO:0005694 | 0.0000420199 | 7.7619834711 | 1 | 8 | 338 | chromosome |
| GO:0000793 | 0.0000487416 | 15.0041237113 | 0 | 5 | 102 | condensed chromosome |
| GO:0043231 | 0.0001314358 | 8.4279817510 | 19 | 28 | 4631 | intracellular membrane-bounded organelle |
| GO:0043227 | 0.0001350358 | 8.4036458333 | 19 | 28 | 4636 | membrane-bounded organelle |
| GO:0044446 | 0.0003038169 | 4.0117539180 | 12 | 22 | 3021 | intracellular organelle part |
| GO:0044422 | 0.0003715265 | 3.9315485997 | 12 | 22 | 3057 | organelle part |
| GO:0044427 | 0.0008389367 | 6.3575268817 | 1 | 6 | 285 | chromosomal part |
| GO:0000775 | 0.0009708305 | 10.4485981308 | 0 | 4 | 111 | chromosome, centromeric region |

  


Gene to GO MF test for over-representation

| GOMFID | Pvalue | OddsRatio | ExpCount | Count | Size | Term |
| GO:0003723 | 0.0005918911 | 5.1773833770 | 2 | 8 | 499 | RNA binding |

  


Gene to KEGG test for over-representation

| KEGGID | Pvalue | OddsRatio | ExpCount | Count | Size | Term |
| 03040 | 0.0000741277 | 16.0348583878 | 0 | 5 | 107 | Spliceosome |

  


Gene to ChrMap test for over-representation

| ChrMapID | Pvalue | OddsRatio | ExpCount | Count | Size |
| 4 | 0.0000000005 | 17.0553745928 | 1 | 12 | 319 |
| 4q | 0.0000001187 | 14.6471674877 | 1 | 9 | 241 |
| 4q2 | 0.0000001571 | 22.2754940711 | 0 | 7 | 117 |
| 4q21 | 0.0004622559 | 23.1396011396 | 0 | 3 | 42 |

  
  

# CGS 7 HIST1H2BK n= 10


Gene to GO BP test for over-representation

| GOBPID | Pvalue | OddsRatio | ExpCount | Count | Size | Term |
| GO:0006334 | 0.0000000000 | Inf | 0 | 5 | 41 | nucleosome assembly |
| GO:0031497 | 0.0000000000 | Inf | 0 | 5 | 43 | chromatin assembly |
| GO:0034728 | 0.0000000000 | Inf | 0 | 5 | 48 | nucleosome organization |
| GO:0065004 | 0.0000000000 | Inf | 0 | 5 | 53 | protein-DNA complex assembly |
| GO:0006323 | 0.0000000001 | Inf | 0 | 5 | 63 | DNA packaging |
| GO:0006333 | 0.0000000001 | Inf | 0 | 5 | 72 | chromatin assembly or disassembly |
| GO:0071103 | 0.0000000002 | Inf | 0 | 5 | 82 | DNA conformation change |
| GO:0006325 | 0.0000000698 | Inf | 0 | 5 | 258 | chromatin organization |
| GO:0034622 | 0.0000000769 | Inf | 0 | 5 | 263 | cellular macromolecular complex assembly |
| GO:0034621 | 0.0000001622 | Inf | 0 | 5 | 305 | cellular macromolecular complex subunit organization |
| GO:0051276 | 0.0000003150 | Inf | 0 | 5 | 348 | chromosome organization |
| GO:0065003 | 0.0000024384 | Inf | 0 | 5 | 523 | macromolecular complex assembly |
| GO:0043933 | 0.0000037554 | Inf | 0 | 5 | 570 | macromolecular complex subunit organization |
| GO:0022607 | 0.0000078949 | Inf | 0 | 5 | 661 | cellular component assembly |
| GO:0044085 | 0.0000157906 | Inf | 1 | 5 | 759 | cellular component biogenesis |
| GO:0006996 | 0.0000520641 | Inf | 1 | 5 | 963 | organelle organization |
| GO:0042742 | 0.0006349346 | 84.5925925926 | 0 | 2 | 56 | defense response to bacterium |

  


Gene to GO CC test for over-representation

| GOCCID | Pvalue | OddsRatio | ExpCount | Count | Size | Term |
| GO:0000786 | 0.0000000000 | Inf | 0 | 5 | 24 | nucleosome |
| GO:0032993 | 0.0000000000 | Inf | 0 | 5 | 50 | protein-DNA complex |
| GO:0000785 | 0.0000000017 | Inf | 0 | 5 | 133 | chromatin |
| GO:0044427 | 0.0000000817 | Inf | 0 | 5 | 285 | chromosomal part |
| GO:0005694 | 0.0000001927 | Inf | 0 | 5 | 338 | chromosome |
| GO:0043228 | 0.0004631220 | Inf | 1 | 5 | 1596 | non-membrane-bounded organelle |
| GO:0043232 | 0.0004631220 | Inf | 1 | 5 | 1596 | intracellular non-membrane-bounded organelle |

  


Gene to GO MF test for over-representation

| GOMFID | Pvalue | OddsRatio | ExpCount | Count | Size | Term |
| GO:0003677 | 0.0000313974 | Inf | 1 | 5 | 906 | DNA binding |
| GO:0003676 | 0.0003594112 | Inf | 1 | 5 | 1474 | nucleic acid binding |

  


Gene to KEGG test for over-representation

| KEGGID | Pvalue | OddsRatio | ExpCount | Count | Size | Term |
| 05322 | 0.0000000000 | Inf | 0 | 10 | 65 | Systemic lupus erythematosus |

  


Gene to ChrMap test for over-representation

| ChrMapID | Pvalue | OddsRatio | ExpCount | Count | Size |
| 6p21.3 | 0.0000000000 | 660.3545454545 | 0 | 9 | 119 |
| 6p2 | 0.0000000000 | Inf | 0 | 10 | 252 |
| 6p | 0.0000000000 | Inf | 0 | 10 | 287 |
| 6p21 | 0.0000000000 | 442.7116564417 | 0 | 9 | 172 |
| 6 | 0.0000000000 | Inf | 1 | 10 | 478 |

  
  

# CGS 8 HLA-DQA1 n= 2


Gene to GO BP test for over-representation

| GOBPID | Pvalue | OddsRatio | ExpCount | Count | Size | Term |
| GO:0002504 | 0.0000032667 | Inf | 0 | 2 | 13 | antigen processing and presentation of peptide or polysaccharide antigen via MHC class II |
| GO:0019882 | 0.0000414616 | Inf | 0 | 2 | 45 | antigen processing and presentation |

  


Gene to GO CC test for over-representation

| GOCCID | Pvalue | OddsRatio | ExpCount | Count | Size | Term |
| GO:0042611 | 0.0000076626 | Inf | 0 | 2 | 21 | MHC protein complex |
| GO:0005765 | 0.0000522150 | Inf | 0 | 2 | 54 | lysosomal membrane |
| GO:0005774 | 0.0000932646 | Inf | 0 | 2 | 72 | vacuolar membrane |
| GO:0044437 | 0.0001012555 | Inf | 0 | 2 | 75 | vacuolar part |
| GO:0010008 | 0.0003059559 | Inf | 0 | 2 | 130 | endosome membrane |
| GO:0044440 | 0.0003059559 | Inf | 0 | 2 | 130 | endosomal part |
| GO:0000323 | 0.0003862306 | Inf | 0 | 2 | 146 | lytic vacuole |
| GO:0005764 | 0.0003862306 | Inf | 0 | 2 | 146 | lysosome |
| GO:0005773 | 0.0005179905 | Inf | 0 | 2 | 169 | vacuole |

  
  


Gene to KEGG test for over-representation

| KEGGID | Pvalue | OddsRatio | ExpCount | Count | Size | Term |
| 05310 | 0.0000224346 | Inf | 0 | 2 | 15 | Asthma |
| 05320 | 0.0000694404 | Inf | 0 | 2 | 26 | Autoimmune thyroid disease |
| 05330 | 0.0000749957 | Inf | 0 | 2 | 27 | Allograft rejection |
| 05332 | 0.0000807646 | Inf | 0 | 2 | 28 | Graft-versus-host disease |
| 04940 | 0.0001059768 | Inf | 0 | 2 | 32 | Type I diabetes mellitus |
| 04672 | 0.0001128140 | Inf | 0 | 2 | 33 | Intestinal immune network for IgA production |
| 05416 | 0.0002724202 | Inf | 0 | 2 | 51 | Viral myocarditis |
| 04612 | 0.0003057516 | Inf | 0 | 2 | 54 | Antigen processing and presentation |
| 05140 | 0.0003057516 | Inf | 0 | 2 | 54 | Leishmaniasis |
| 05322 | 0.0004444188 | Inf | 0 | 2 | 65 | Systemic lupus erythematosus |
| 04514 | 0.0007627765 | Inf | 0 | 2 | 85 | Cell adhesion molecules (CAMs) |
| 04145 | 0.0013520587 | Inf | 0 | 2 | 113 | Phagosome |

  


Gene to ChrMap test for over-representation

| ChrMapID | Pvalue | OddsRatio | ExpCount | Count | Size |
| 6p21.3 | 0.0002093188 | Inf | 0 | 2 | 119 |
| 6p21 | 0.0004384335 | Inf | 0 | 2 | 172 |
| 6p2 | 0.0009428735 | Inf | 0 | 2 | 252 |

  
  

# CGS 9 MT1H n= 8

  
  


Gene to GO MF test for over-representation

| GOMFID | Pvalue | OddsRatio | ExpCount | Count | Size | Term |
| GO:0046872 | 0.0000432161 | Inf | 2 | 7 | 1714 | metal ion binding |
| GO:0043169 | 0.0000480280 | Inf | 2 | 7 | 1740 | cation binding |
| GO:0043167 | 0.0000492014 | Inf | 2 | 7 | 1746 | ion binding |
| GO:0005507 | 0.0004225489 | 92.3225806452 | 0 | 2 | 33 | copper ion binding |

  
  


Gene to ChrMap test for over-representation

| ChrMapID | Pvalue | OddsRatio | ExpCount | Count | Size |
| 16q13 | 0.0000000000 | 6357.5555555556 | 0 | 7 | 16 |
| 16q1 | 0.0000000000 | 1783.0312500000 | 0 | 7 | 39 |
| 16q | 0.0000000000 | 355.5379746835 | 0 | 7 | 165 |
| 16 | 0.0000000017 | 162.9732937685 | 0 | 7 | 344 |

  
  

# CGS 10 PTGER3 n= 30


Gene to GO BP test for over-representation

| GOBPID | Pvalue | OddsRatio | ExpCount | Count | Size | Term |
| GO:0007606 | 0.0008512464 | 58.7521367521 | 0 | 2 | 11 | sensory perception of chemical stimulus |

  


Gene to GO CC test for over-representation

| GOCCID | Pvalue | OddsRatio | ExpCount | Count | Size | Term |
| GO:0044459 | 0.0001996472 | 4.8980589207 | 3 | 11 | 919 | plasma membrane part |

  


Gene to GO MF test for over-representation

| GOMFID | Pvalue | OddsRatio | ExpCount | Count | Size | Term |
| GO:0004871 | 0.0005583090 | 4.4349030471 | 3 | 10 | 770 | signal transducer activity |
| GO:0060089 | 0.0005583090 | 4.4349030471 | 3 | 10 | 770 | molecular transducer activity |

  


Gene to KEGG test for over-representation

| KEGGID | Pvalue | OddsRatio | ExpCount | Count | Size | Term |
| 04080 | 0.0010577109 | 19.6842105263 | 0 | 3 | 60 | Neuroactive ligand-receptor interaction |
| 04020 | 0.0028083458 | 13.7407407407 | 0 | 3 | 84 | Calcium signaling pathway |
| 04730 | 0.0085070009 | 17.6081871345 | 0 | 2 | 40 | Long-term depression |
| 05414 | 0.0093536150 | 16.7166666667 | 0 | 2 | 42 | Dilated cardiomyopathy |

  


Gene to ChrMap test for over-representation

| ChrMapID | Pvalue | OddsRatio | ExpCount | Count | Size |
| 8p12 | 0.0006987925 | 64.6984126984 | 0 | 2 | 11 |

  
  

# CGS 11 HLA-DQB1 n= 7


Gene to GO BP test for over-representation

| GOBPID | Pvalue | OddsRatio | ExpCount | Count | Size | Term |
| GO:0002504 | 0.0000000000 | Inf | 0 | 6 | 13 | antigen processing and presentation of peptide or polysaccharide antigen via MHC class II |
| GO:0019882 | 0.0000000000 | Inf | 0 | 6 | 45 | antigen processing and presentation |
| GO:0006955 | 0.0000000985 | Inf | 0 | 6 | 472 | immune response |
| GO:0002376 | 0.0000012135 | Inf | 1 | 6 | 716 | immune system process |
| GO:0050896 | 0.0004093609 | Inf | 2 | 6 | 1885 | response to stimulus |

  


Gene to GO CC test for over-representation

| GOCCID | Pvalue | OddsRatio | ExpCount | Count | Size | Term |
| GO:0042611 | 0.0000000000 | Inf | 0 | 6 | 21 | MHC protein complex |
| GO:0005765 | 0.0000000000 | Inf | 0 | 6 | 54 | lysosomal membrane |
| GO:0005774 | 0.0000000000 | Inf | 0 | 6 | 72 | vacuolar membrane |
| GO:0044437 | 0.0000000000 | Inf | 0 | 6 | 75 | vacuolar part |
| GO:0010008 | 0.0000000000 | Inf | 0 | 6 | 130 | endosome membrane |
| GO:0044440 | 0.0000000000 | Inf | 0 | 6 | 130 | endosomal part |
| GO:0000323 | 0.0000000001 | Inf | 0 | 6 | 146 | lytic vacuole |
| GO:0005764 | 0.0000000001 | Inf | 0 | 6 | 146 | lysosome |
| GO:0005773 | 0.0000000001 | Inf | 0 | 6 | 169 | vacuole |
| GO:0005768 | 0.0000000011 | Inf | 0 | 6 | 239 | endosome |
| GO:0005770 | 0.0000000853 | 236.6451612903 | 0 | 4 | 66 | late endosome |
| GO:0005789 | 0.0000014217 | 100.6857142857 | 0 | 5 | 355 | endoplasmic reticulum membrane |
| GO:0042175 | 0.0000016107 | 98.0362116992 | 0 | 5 | 364 | nuclear membrane-endoplasmic reticulum network |
| GO:0031902 | 0.0000019171 | 230.1875000000 | 0 | 3 | 35 | late endosome membrane |
| GO:0044432 | 0.0000024818 | 89.3622448980 | 0 | 5 | 397 | endoplasmic reticulum part |
| GO:0071212 | 0.0000029490 | 86.1083743842 | 0 | 5 | 411 | subsynaptic reticulum |
| GO:0044459 | 0.0000036047 | Inf | 1 | 6 | 919 | plasma membrane part |
| GO:0005794 | 0.0000100414 | 65.9980806142 | 0 | 5 | 526 | Golgi apparatus |
| GO:0031090 | 0.0000103421 | Inf | 1 | 6 | 1095 | organelle membrane |
| GO:0005783 | 0.0000180283 | 58.0153321976 | 0 | 5 | 592 | endoplasmic reticulum |
| GO:0005886 | 0.0000713878 | Inf | 1 | 6 | 1510 | plasma membrane |
| GO:0043234 | 0.0000944491 | Inf | 1 | 6 | 1582 | protein complex |
| GO:0012505 | 0.0000993354 | 39.4591346154 | 1 | 5 | 837 | endomembrane system |
| GO:0016021 | 0.0002605114 | Inf | 2 | 6 | 1873 | integral to membrane |
| GO:0031224 | 0.0002866077 | Inf | 2 | 6 | 1903 | intrinsic to membrane |
| GO:0032991 | 0.0003349318 | Inf | 2 | 6 | 1953 | macromolecular complex |

  


Gene to GO MF test for over-representation

| GOMFID | Pvalue | OddsRatio | ExpCount | Count | Size | Term |
| GO:0004888 | 0.0000035654 | Inf | 0 | 4 | 314 | transmembrane receptor activity |
| GO:0004872 | 0.0000249966 | Inf | 0 | 4 | 510 | receptor activity |
| GO:0004871 | 0.0001304054 | Inf | 0 | 4 | 770 | signal transducer activity |
| GO:0060089 | 0.0001304054 | Inf | 0 | 4 | 770 | molecular transducer activity |

  


Gene to KEGG test for over-representation

| KEGGID | Pvalue | OddsRatio | ExpCount | Count | Size | Term |
| 05310 | 0.0000000000 | Inf | 0 | 6 | 15 | Asthma |
| 05320 | 0.0000000000 | Inf | 0 | 6 | 26 | Autoimmune thyroid disease |
| 05330 | 0.0000000000 | Inf | 0 | 6 | 27 | Allograft rejection |
| 05332 | 0.0000000000 | Inf | 0 | 6 | 28 | Graft-versus-host disease |
| 04940 | 0.0000000000 | Inf | 0 | 6 | 32 | Type I diabetes mellitus |
| 04672 | 0.0000000000 | Inf | 0 | 6 | 33 | Intestinal immune network for IgA production |
| 05416 | 0.0000000000 | Inf | 0 | 6 | 51 | Viral myocarditis |
| 04612 | 0.0000000000 | Inf | 0 | 6 | 54 | Antigen processing and presentation |
| 05140 | 0.0000000000 | Inf | 0 | 6 | 54 | Leishmaniasis |
| 05322 | 0.0000000001 | Inf | 0 | 6 | 65 | Systemic lupus erythematosus |
| 04514 | 0.0000000004 | Inf | 0 | 6 | 85 | Cell adhesion molecules (CAMs) |
| 04145 | 0.0000000022 | Inf | 0 | 6 | 113 | Phagosome |
| 04640 | 0.0048745918 | 27.2636363636 | 0 | 2 | 57 | Hematopoietic cell lineage |

  


Gene to ChrMap test for over-representation

| ChrMapID | Pvalue | OddsRatio | ExpCount | Count | Size |
| 6p21.3 | 0.0000000000 | Inf | 0 | 7 | 119 |
| 6p21 | 0.0000000000 | Inf | 0 | 7 | 172 |
| 6p2 | 0.0000000000 | Inf | 0 | 7 | 252 |
| 6p | 0.0000000001 | Inf | 0 | 7 | 287 |
| 6 | 0.0000000022 | Inf | 0 | 7 | 478 |

  
  

# CGS 12 NME1 n= 18

  


Gene to GO CC test for over-representation

| GOCCID | Pvalue | OddsRatio | ExpCount | Count | Size | Term |
| GO:0031974 | 0.0000000505 | 16.1585551331 | 3 | 14 | 1329 | membrane-enclosed lumen |
| GO:0070013 | 0.0000004265 | 12.5447949527 | 3 | 13 | 1281 | intracellular organelle lumen |
| GO:0043233 | 0.0000005241 | 12.2865116279 | 3 | 13 | 1303 | organelle lumen |
| GO:0044429 | 0.0000237416 | 11.3538961039 | 1 | 7 | 399 | mitochondrial part |
| GO:0043231 | 0.0002120146 | Inf | 11 | 18 | 4631 | intracellular membrane-bounded organelle |
| GO:0043227 | 0.0002161807 | Inf | 11 | 18 | 4636 | membrane-bounded organelle |
| GO:0005739 | 0.0002270856 | 6.9340314136 | 2 | 8 | 772 | mitochondrion |
| GO:0044446 | 0.0002748665 | 7.2854291417 | 7 | 15 | 3021 | intracellular organelle part |
| GO:0044422 | 0.0003212861 | 7.1400394477 | 7 | 15 | 3057 | organelle part |
| GO:0031981 | 0.0003457055 | 6.0409914204 | 3 | 9 | 1058 | nuclear lumen |
| GO:0005665 | 0.0004286032 | 83.8068181818 | 0 | 2 | 13 | DNA-directed RNA polymerase II, core complex |
| GO:0005759 | 0.0004387502 | 13.7828571429 | 0 | 4 | 154 | mitochondrial matrix |
| GO:0031980 | 0.0004387502 | 13.7828571429 | 0 | 4 | 154 | mitochondrial lumen |
| GO:0000428 | 0.0009315436 | 54.1838235294 | 0 | 2 | 19 | DNA-directed RNA polymerase complex |
| GO:0030880 | 0.0009315436 | 54.1838235294 | 0 | 2 | 19 | RNA polymerase complex |
| GO:0055029 | 0.0009315436 | 54.1838235294 | 0 | 2 | 19 | nuclear DNA-directed RNA polymerase complex |
| GO:0005730 | 0.0009916433 | 6.7554027505 | 1 | 6 | 515 | nucleolus |

  
  


Gene to KEGG test for over-representation

| KEGGID | Pvalue | OddsRatio | ExpCount | Count | Size | Term |
| 05016 | 0.0001177886 | 29.5050505051 | 0 | 4 | 136 | Huntington's disease |
| 00240 | 0.0002877910 | 36.7868852459 | 0 | 3 | 64 | Pyrimidine metabolism |
| 00230 | 0.0008986289 | 24.4120879121 | 0 | 3 | 94 | Purine metabolism |
| 03020 | 0.0009229102 | 63.8736842105 | 0 | 2 | 21 | RNA polymerase |
| 01100 | 0.0070566850 | 8.9602102102 | 2 | 5 | 671 | Metabolic pathways |

  


Gene to ChrMap test for over-representation

| ChrMapID | Pvalue | OddsRatio | ExpCount | Count | Size |
| 17q2 | 0.0001175395 | 13.2826086957 | 1 | 5 | 235 |
| 17q | 0.0004224844 | 9.9557186235 | 1 | 5 | 309 |

  
  

# CGS 13 PDE4C n= 30

  
  
  
  
  
  

# CGS 14 STAT1 n= 9


Gene to GO BP test for over-representation

| GOBPID | Pvalue | OddsRatio | ExpCount | Count | Size | Term |
| GO:0016236 | 0.0000641605 | 255.3333333333 | 0 | 2 | 11 | macroautophagy |
| GO:0009267 | 0.0003195530 | 104.2575757576 | 0 | 2 | 24 | cellular response to starvation |
| GO:0006914 | 0.0003471389 | 99.7101449275 | 0 | 2 | 25 | autophagy |
| GO:0031667 | 0.0004319117 | 29.4130434783 | 0 | 3 | 141 | response to nutrient levels |
| GO:0042594 | 0.0005018947 | 81.8452380952 | 0 | 2 | 30 | response to starvation |
| GO:0009991 | 0.0005704759 | 26.6486842105 | 0 | 3 | 155 | response to extracellular stimulus |
| GO:0031669 | 0.0009400855 | 58.6666666667 | 0 | 2 | 41 | cellular response to nutrient levels |

  


Gene to GO CC test for over-representation

| GOCCID | Pvalue | OddsRatio | ExpCount | Count | Size | Term |
| GO:0000323 | 0.0000000014 | 155.4857142857 | 0 | 6 | 146 | lytic vacuole |
| GO:0005764 | 0.0000000014 | 155.4857142857 | 0 | 6 | 146 | lysosome |
| GO:0005773 | 0.0000000035 | 133.1226993865 | 0 | 6 | 169 | vacuole |

  
  


Gene to KEGG test for over-representation

| KEGGID | Pvalue | OddsRatio | ExpCount | Count | Size | Term |
| 04142 | 0.0000182271 | 48.9218106996 | 0 | 4 | 85 | Lysosome |

  
  
  

# CGS 15 GJA1 n= 7


Gene to GO BP test for over-representation

| GOBPID | Pvalue | OddsRatio | ExpCount | Count | Size | Term |
| GO:0035107 | 0.0006673804 | 76.2222222222 | 0 | 2 | 47 | appendage morphogenesis |
| GO:0035108 | 0.0006673804 | 76.2222222222 | 0 | 2 | 47 | limb morphogenesis |
| GO:0035468 | 0.0006841733 | 29.2850877193 | 0 | 3 | 231 | positive regulation of signaling pathway |
| GO:0048736 | 0.0007554043 | 71.4270833333 | 0 | 2 | 50 | appendage development |
| GO:0060173 | 0.0007554043 | 71.4270833333 | 0 | 2 | 50 | limb development |
| GO:0010647 | 0.0008326726 | 27.2991803279 | 0 | 3 | 247 | positive regulation of cell communication |

  


Gene to GO CC test for over-representation

| GOCCID | Pvalue | OddsRatio | ExpCount | Count | Size | Term |
| GO:0031226 | 0.0008140257 | 17.1015576324 | 1 | 4 | 539 | intrinsic to plasma membrane |

  
  


Gene to KEGG test for over-representation

| KEGGID | Pvalue | OddsRatio | ExpCount | Count | Size | Term |
| 05412 | 0.0004470785 | 167.8333333333 | 0 | 2 | 38 | Arrhythmogenic right ventricular cardiomyopathy (ARVC) |

  
  
  

# CGS 16 HLA-DRB4 n= 2

  
  
  


Gene to KEGG test for over-representation

| KEGGID | Pvalue | OddsRatio | ExpCount | Count | Size | Term |
| 05310 | 0.0049019608 | Inf | 0 | 1 | 15 | Asthma |
| 05320 | 0.0084967320 | Inf | 0 | 1 | 26 | Autoimmune thyroid disease |
| 05330 | 0.0088235294 | Inf | 0 | 1 | 27 | Allograft rejection |
| 05332 | 0.0091503268 | Inf | 0 | 1 | 28 | Graft-versus-host disease |

  
  
  

# CGS 17 RPL29 n= 30


Gene to GO BP test for over-representation

| GOBPID | Pvalue | OddsRatio | ExpCount | Count | Size | Term |
| GO:0006414 | 0.0000000000 | Inf | 0 | 29 | 96 | translational elongation |
| GO:0006412 | 0.0000000000 | Inf | 1 | 29 | 299 | translation |
| GO:0044267 | 0.0000000000 | Inf | 6 | 29 | 1507 | cellular protein metabolic process |
| GO:0019538 | 0.0000000000 | Inf | 7 | 29 | 1718 | protein metabolic process |
| GO:0034645 | 0.0000000000 | Inf | 8 | 29 | 1848 | cellular macromolecule biosynthetic process |
| GO:0009059 | 0.0000000000 | Inf | 8 | 29 | 1887 | macromolecule biosynthetic process |
| GO:0010467 | 0.0000000000 | Inf | 9 | 29 | 2040 | gene expression |
| GO:0044249 | 0.0000000000 | Inf | 10 | 29 | 2292 | cellular biosynthetic process |
| GO:0009058 | 0.0000000000 | Inf | 10 | 29 | 2345 | biosynthetic process |
| GO:0044260 | 0.0000000001 | Inf | 13 | 29 | 3142 | cellular macromolecule metabolic process |
| GO:0043170 | 0.0000000011 | Inf | 14 | 29 | 3403 | macromolecule metabolic process |
| GO:0042254 | 0.0000001696 | 22.2563261481 | 0 | 7 | 104 | ribosome biogenesis |
| GO:0044237 | 0.0000002001 | Inf | 17 | 29 | 4066 | cellular metabolic process |
| GO:0044238 | 0.0000002292 | Inf | 17 | 29 | 4085 | primary metabolic process |
| GO:0022613 | 0.0000029221 | 14.1833232992 | 1 | 7 | 158 | ribonucleoprotein complex biogenesis |
| GO:0008152 | 0.0000038261 | Inf | 19 | 29 | 4500 | metabolic process |
| GO:0042273 | 0.0000107189 | 99.1442307692 | 0 | 3 | 11 | ribosomal large subunit biogenesis |
| GO:0006364 | 0.0000145105 | 19.7048611111 | 0 | 5 | 77 | rRNA processing |
| GO:0016072 | 0.0000154593 | 19.4320776256 | 0 | 5 | 78 | rRNA metabolic process |
| GO:0042274 | 0.0000184748 | 79.2923076923 | 0 | 3 | 13 | ribosomal small subunit biogenesis |
| GO:0034470 | 0.0002005366 | 10.9928385417 | 1 | 5 | 133 | ncRNA processing |
| GO:0034660 | 0.0004087709 | 9.3500000000 | 1 | 5 | 155 | ncRNA metabolic process |

  


Gene to GO CC test for over-representation

| GOCCID | Pvalue | OddsRatio | ExpCount | Count | Size | Term |
| GO:0005840 | 0.0000000000 | 539.4793388430 | 1 | 27 | 148 | ribosome |
| GO:0022626 | 0.0000000000 | 411.0969387755 | 0 | 22 | 71 | cytosolic ribosome |
| GO:0033279 | 0.0000000000 | 264.0723684211 | 0 | 22 | 98 | ribosomal subunit |
| GO:0030529 | 0.0000000000 | 187.3491124260 | 1 | 27 | 365 | ribonucleoprotein complex |
| GO:0044445 | 0.0000000000 | 164.7152103560 | 1 | 21 | 124 | cytosolic part |
| GO:0005829 | 0.0000000000 | 202.4350649351 | 4 | 29 | 953 | cytosol |
| GO:0022625 | 0.0000000000 | 222.7878787879 | 0 | 12 | 34 | cytosolic large ribosomal subunit |
| GO:0015934 | 0.0000000000 | 151.6391096979 | 0 | 13 | 50 | large ribosomal subunit |
| GO:0032991 | 0.0000000000 | 82.1465696466 | 8 | 29 | 1953 | macromolecular complex |
| GO:0043228 | 0.0000000000 | 33.2982791587 | 6 | 27 | 1596 | non-membrane-bounded organelle |
| GO:0043232 | 0.0000000000 | 33.2982791587 | 6 | 27 | 1596 | intracellular non-membrane-bounded organelle |
| GO:0022627 | 0.0000000000 | 121.1208791209 | 0 | 9 | 35 | cytosolic small ribosomal subunit |
| GO:0015935 | 0.0000000000 | 78.5785714286 | 0 | 9 | 49 | small ribosomal subunit |
| GO:0044444 | 0.0000000002 | 40.9300196207 | 13 | 29 | 3087 | cytoplasmic part |
| GO:0005737 | 0.0000002149 | Inf | 18 | 30 | 4444 | cytoplasm |
| GO:0044446 | 0.0003038169 | 4.0117539180 | 12 | 22 | 3021 | intracellular organelle part |
| GO:0044422 | 0.0003715265 | 3.9315485997 | 12 | 22 | 3057 | organelle part |

  


Gene to GO MF test for over-representation

| GOMFID | Pvalue | OddsRatio | ExpCount | Count | Size | Term |
| GO:0003735 | 0.0000000000 | 662.5312500000 | 1 | 27 | 123 | structural constituent of ribosome |
| GO:0005198 | 0.0000000000 | 190.5882352941 | 1 | 27 | 350 | structural molecule activity |
| GO:0003723 | 0.0000000000 | 38.5461215933 | 2 | 22 | 499 | RNA binding |
| GO:0003676 | 0.0000000000 | 15.7600000000 | 6 | 24 | 1474 | nucleic acid binding |

  


Gene to KEGG test for over-representation

| KEGGID | Pvalue | OddsRatio | ExpCount | Count | Size | Term |
| 03010 | 0.0000000000 | Inf | 1 | 27 | 84 | Ribosome |

  


Gene to ChrMap test for over-representation

| ChrMapID | Pvalue | OddsRatio | ExpCount | Count | Size |
| 3p2 | 0.0004199968 | 9.2346820809 | 1 | 5 | 178 |
| 3p | 0.0009712800 | 7.6095693780 | 1 | 5 | 214 |

  
  

# CGS 18 IFI27 n= 5

  
  
  
  
  
  

# CGS 19 GZMB n= 3


Gene to GO BP test for over-representation

| GOBPID | Pvalue | OddsRatio | ExpCount | Count | Size | Term |
| GO:0019835 | 0.0000097896 | 1254.0000000000 | 0 | 2 | 13 | cytolysis |

  
  


Gene to GO MF test for over-representation

| GOMFID | Pvalue | OddsRatio | ExpCount | Count | Size | Term |
| GO:0004252 | 0.0000639062 | Inf | 0 | 2 | 58 | serine-type endopeptidase activity |
| GO:0008236 | 0.0000880692 | Inf | 0 | 2 | 68 | serine-type peptidase activity |
| GO:0017171 | 0.0000933657 | Inf | 0 | 2 | 70 | serine hydrolase activity |
| GO:0004175 | 0.0005818829 | Inf | 0 | 2 | 174 | endopeptidase activity |

  


Gene to KEGG test for over-representation

| KEGGID | Pvalue | OddsRatio | ExpCount | Count | Size | Term |
| 05320 | 0.0084967320 | Inf | 0 | 1 | 26 | Autoimmune thyroid disease |
| 05330 | 0.0088235294 | Inf | 0 | 1 | 27 | Allograft rejection |
| 05332 | 0.0091503268 | Inf | 0 | 1 | 28 | Graft-versus-host disease |

  


Gene to ChrMap test for over-representation

| ChrMapID | Pvalue | OddsRatio | ExpCount | Count | Size |
| 14q11.2 | 0.0000530737 | 494.2424242424 | 0 | 2 | 35 |
| 14q11 | 0.0000660752 | 440.5945945946 | 0 | 2 | 39 |
| 14q1 | 0.0002601027 | 216.3466666667 | 0 | 2 | 77 |

  
  

# CGS 20 TPR n= 11


Gene to GO BP test for over-representation

| GOBPID | Pvalue | OddsRatio | ExpCount | Count | Size | Term |
| GO:0006913 | 0.0008737326 | 21.9090909091 | 0 | 3 | 157 | nucleocytoplasmic transport |
| GO:0051169 | 0.0008737326 | 21.9090909091 | 0 | 3 | 157 | nuclear transport |

  
  


Gene to GO MF test for over-representation

| GOMFID | Pvalue | OddsRatio | ExpCount | Count | Size | Term |
| GO:0042277 | 0.0004072600 | 27.8138925295 | 0 | 3 | 112 | peptide binding |
| GO:0003755 | 0.0004723916 | 81.3750000000 | 0 | 2 | 24 | peptidyl-prolyl cis-trans isomerase activity |
| GO:0016859 | 0.0005554332 | 74.5729166667 | 0 | 2 | 26 | cis-trans isomerase activity |

  
  
  
  

# CGS 21 C1QBP n= 8

  
  
  
  


Gene to ChrMap test for over-representation

| ChrMapID | Pvalue | OddsRatio | ExpCount | Count | Size |
| 17p1 | 0.0000000000 | 518.5137614679 | 0 | 7 | 116 |
| 17p | 0.0000000000 | 509.0450450450 | 0 | 7 | 118 |
| 17p13 | 0.0000000000 | 324.3200000000 | 0 | 6 | 81 |
| 17 | 0.0000000079 | 128.7369668246 | 0 | 7 | 429 |
| 17p13.3 | 0.0002681367 | 113.3194444444 | 0 | 2 | 26 |

  
  

# CGS 22 S100A9 n= 2


Gene to GO BP test for over-representation

| GOBPID | Pvalue | OddsRatio | ExpCount | Count | Size | Term |
| GO:0006935 | 0.0003788084 | Inf | 0 | 2 | 135 | chemotaxis |
| GO:0042330 | 0.0003788084 | Inf | 0 | 2 | 135 | taxis |
| GO:0007626 | 0.0006230969 | Inf | 0 | 2 | 173 | locomotory behavior |

  
  
  
  


Gene to ChrMap test for over-representation

| ChrMapID | Pvalue | OddsRatio | ExpCount | Count | Size |
| 1q21 | 0.0001303137 | Inf | 0 | 2 | 94 |
| 1q2 | 0.0008127387 | Inf | 0 | 2 | 234 |

  
  

# CGS 23 UGT2B17 n= 2


Gene to GO BP test for over-representation

| GOBPID | Pvalue | OddsRatio | ExpCount | Count | Size | Term |
| GO:0008202 | 0.0003731964 | Inf | 0 | 2 | 134 | steroid metabolic process |

  


Gene to GO CC test for over-representation

| GOCCID | Pvalue | OddsRatio | ExpCount | Count | Size | Term |
| GO:0005792 | 0.0003399267 | Inf | 0 | 2 | 137 | microsome |
| GO:0042598 | 0.0003550330 | Inf | 0 | 2 | 140 | vesicular fraction |

  


Gene to GO MF test for over-representation

| GOMFID | Pvalue | OddsRatio | ExpCount | Count | Size | Term |
| GO:0015020 | 0.0000017397 | Inf | 0 | 2 | 10 | glucuronosyltransferase activity |
| GO:0005501 | 0.0000021263 | Inf | 0 | 2 | 11 | retinoid binding |
| GO:0019840 | 0.0000030155 | Inf | 0 | 2 | 13 | isoprenoid binding |
| GO:0033293 | 0.0000257481 | Inf | 0 | 2 | 37 | monocarboxylic acid binding |
| GO:0008194 | 0.0000553235 | Inf | 0 | 2 | 54 | UDP-glycosyltransferase activity |
| GO:0016758 | 0.0001191138 | Inf | 0 | 2 | 79 | transferase activity, transferring hexosyl groups |
| GO:0031406 | 0.0001380189 | Inf | 0 | 2 | 85 | carboxylic acid binding |
| GO:0016757 | 0.0002668751 | Inf | 0 | 2 | 118 | transferase activity, transferring glycosyl groups |
| GO:0008289 | 0.0009742509 | Inf | 0 | 2 | 225 | lipid binding |

  


Gene to KEGG test for over-representation

| KEGGID | Pvalue | OddsRatio | ExpCount | Count | Size | Term |
| 00140 | 0.0000326904 | Inf | 0 | 2 | 18 | Steroid hormone biosynthesis |
| 00500 | 0.0000405959 | Inf | 0 | 2 | 20 | Starch and sucrose metabolism |
| 00830 | 0.0000493561 | Inf | 0 | 2 | 22 | Retinol metabolism |
| 00860 | 0.0000493561 | Inf | 0 | 2 | 22 | Porphyrin and chlorophyll metabolism |
| 00983 | 0.0000589710 | Inf | 0 | 2 | 24 | Drug metabolism - other enzymes |
| 00980 | 0.0001198649 | Inf | 0 | 2 | 34 | Metabolism of xenobiotics by cytochrome P450 |
| 00982 | 0.0001346076 | Inf | 0 | 2 | 36 | Drug metabolism - cytochrome P450 |

  


Gene to ChrMap test for over-representation

| ChrMapID | Pvalue | OddsRatio | ExpCount | Count | Size |
| 4q13 | 0.0000040546 | Inf | 0 | 2 | 17 |
| 4q1 | 0.0000209587 | Inf | 0 | 2 | 38 |
| 4q | 0.0008621989 | Inf | 0 | 2 | 241 |

  
  

# CGS 24 DUSP1 n= 3


Gene to GO BP test for over-representation

| GOBPID | Pvalue | OddsRatio | ExpCount | Count | Size | Term |
| GO:0051591 | 0.0000439937 | 550.6400000000 | 0 | 2 | 27 | response to cAMP |
| GO:0009605 | 0.0001702783 | Inf | 0 | 3 | 384 | response to external stimulus |
| GO:0000302 | 0.0002065628 | 244.7142857143 | 0 | 2 | 58 | response to reactive oxygen species |
| GO:0051384 | 0.0002362121 | 228.2666666667 | 0 | 2 | 62 | response to glucocorticoid stimulus |
| GO:0031960 | 0.0002678362 | 213.8750000000 | 0 | 2 | 66 | response to corticosteroid stimulus |
| GO:0009416 | 0.0003279232 | 192.5915492958 | 0 | 2 | 73 | response to light stimulus |
| GO:0051789 | 0.0004345200 | 166.4878048780 | 0 | 2 | 84 | response to protein stimulus |
| GO:0010033 | 0.0005524764 | Inf | 0 | 3 | 568 | response to organic substance |
| GO:0032870 | 0.0007726966 | 123.6000000000 | 0 | 2 | 112 | cellular response to hormone stimulus |
| GO:0071495 | 0.0008005097 | 121.3571428571 | 0 | 2 | 114 | cellular response to endogenous stimulus |

  
  
  


Gene to KEGG test for over-representation

| KEGGID | Pvalue | OddsRatio | ExpCount | Count | Size | Term |
| 04010 | 0.0027863777 | Inf | 0 | 2 | 162 | MAPK signaling pathway |

  
  
  

# CGS 25 SCD5 n= 6

  
  
  
  
  
  

# CGS 26 CCL21 n= 2

  
  
  
  
  
  

# CGS 27 GNAQ n= 11

  
  
  
  
  
  

# CGS 28 MRPL3 n= 12

  


Gene to GO CC test for over-representation

| GOCCID | Pvalue | OddsRatio | ExpCount | Count | Size | Term |
| GO:0000313 | 0.0007971422 | 60.6255144033 | 0 | 2 | 29 | organellar ribosome |
| GO:0005761 | 0.0007971422 | 60.6255144033 | 0 | 2 | 29 | mitochondrial ribosome |

  
  
  


Gene to ChrMap test for over-representation

| ChrMapID | Pvalue | OddsRatio | ExpCount | Count | Size |
| 3q | 0.0000000004 | 62.9126984127 | 0 | 8 | 260 |
| 3 | 0.0000000013 | 49.7677419355 | 1 | 9 | 474 |
| 3q2 | 0.0000052816 | 29.7135416667 | 0 | 5 | 197 |
| 3q1 | 0.0000745839 | 48.3511904762 | 0 | 3 | 59 |
| 3q23 | 0.0000879703 | 204.2750000000 | 0 | 2 | 10 |

  
  

# CGS 29 NUSAP1 n= 8


Gene to GO BP test for over-representation

| GOBPID | Pvalue | OddsRatio | ExpCount | Count | Size | Term |
| GO:0000279 | 0.0000016654 | 63.1273764259 | 0 | 5 | 268 | M phase |
| GO:0007049 | 0.0000062214 | 54.5614035088 | 1 | 6 | 690 | cell cycle |
| GO:0022403 | 0.0000067072 | 46.8142857143 | 0 | 5 | 355 | cell cycle phase |
| GO:0000280 | 0.0000205426 | 46.6111111111 | 0 | 4 | 196 | nuclear division |
| GO:0007067 | 0.0000205426 | 46.6111111111 | 0 | 4 | 196 | mitosis |
| GO:0048285 | 0.0000226976 | 45.3942470389 | 0 | 4 | 201 | organelle fission |
| GO:0000087 | 0.0000236048 | 44.9246231156 | 0 | 4 | 203 | M phase of mitotic cell cycle |
| GO:0022402 | 0.0000253207 | 35.0217391304 | 0 | 5 | 465 | cell cycle process |
| GO:0051301 | 0.0000449780 | 37.8382978723 | 0 | 4 | 239 | cell division |
| GO:0000278 | 0.0002284616 | 24.3798882682 | 0 | 4 | 362 | mitotic cell cycle |
| GO:0006281 | 0.0008360879 | 24.7573891626 | 0 | 3 | 206 | DNA repair |

  


Gene to GO CC test for over-representation

| GOCCID | Pvalue | OddsRatio | ExpCount | Count | Size | Term |
| GO:0005694 | 0.0000037494 | 53.0330330330 | 0 | 5 | 338 | chromosome |
| GO:0043228 | 0.0005686745 | 21.9132075472 | 2 | 6 | 1596 | non-membrane-bounded organelle |
| GO:0043232 | 0.0005686745 | 21.9132075472 | 2 | 6 | 1596 | intracellular non-membrane-bounded organelle |
| GO:0031981 | 0.0009646250 | 15.0617283951 | 1 | 5 | 1058 | nuclear lumen |

  
  
  


Gene to ChrMap test for over-representation

| ChrMapID | Pvalue | OddsRatio | ExpCount | Count | Size |
| 15q15 | 0.0000000027 | 453.6111111111 | 0 | 4 | 22 |
| 15 | 0.0000000167 | 101.0211864407 | 0 | 6 | 242 |
| 15q | 0.0000000167 | 101.0211864407 | 0 | 6 | 242 |
| 15q1 | 0.0000000992 | 169.4791666667 | 0 | 4 | 52 |

  
  

# CGS 30 P2RX5 n= 2

  
  
  
  


Gene to ChrMap test for over-representation

| ChrMapID | Pvalue | OddsRatio | ExpCount | Count | Size |
| 17p13 | 0.0000965949 | Inf | 0 | 2 | 81 |
| 17p1 | 0.0001988543 | Inf | 0 | 2 | 116 |
| 17p | 0.0002058008 | Inf | 0 | 2 | 118 |

  
  

# CGS 31 CD8A n= 6


Gene to GO BP test for over-representation

| GOBPID | Pvalue | OddsRatio | ExpCount | Count | Size | Term |
| GO:0023033 | 0.0000664847 | Inf | 1 | 6 | 1393 | signaling pathway |
| GO:0007166 | 0.0000828302 | 41.1564171123 | 1 | 5 | 753 | cell surface receptor linked signaling pathway |
| GO:0042110 | 0.0001799382 | 46.9513888889 | 0 | 3 | 147 | T cell activation |
| GO:0007169 | 0.0003173503 | 38.4571428571 | 0 | 3 | 178 | transmembrane receptor protein tyrosine kinase signaling pathway |
| GO:0023052 | 0.0003914308 | Inf | 2 | 6 | 1871 | signaling |
| GO:0046649 | 0.0004815315 | 33.1831683168 | 0 | 3 | 205 | lymphocyte activation |
| GO:0045321 | 0.0008033816 | 27.6514522822 | 0 | 3 | 244 | leukocyte activation |

  


Gene to GO CC test for over-representation

| GOCCID | Pvalue | OddsRatio | ExpCount | Count | Size | Term |
| GO:0042101 | 0.0000300055 | 410.5000000000 | 0 | 2 | 11 | T cell receptor complex |
| GO:0005886 | 0.0000713878 | Inf | 1 | 6 | 1510 | plasma membrane |
| GO:0016021 | 0.0002605114 | Inf | 2 | 6 | 1873 | integral to membrane |
| GO:0031224 | 0.0002866077 | Inf | 2 | 6 | 1903 | intrinsic to membrane |
| GO:0005887 | 0.0003544048 | 25.9697542533 | 0 | 4 | 533 | integral to plasma membrane |
| GO:0031226 | 0.0003701726 | 25.6560747664 | 0 | 4 | 539 | intrinsic to plasma membrane |

  


Gene to GO MF test for over-representation

| GOMFID | Pvalue | OddsRatio | ExpCount | Count | Size | Term |
| GO:0015026 | 0.0000001170 | 652.3636363636 | 0 | 3 | 14 | coreceptor activity |
| GO:0042287 | 0.0000525375 | 298.9583333333 | 0 | 2 | 14 | MHC protein binding |
| GO:0004872 | 0.0003339602 | 26.4071146245 | 0 | 4 | 510 | receptor activity |

  


Gene to KEGG test for over-representation

| KEGGID | Pvalue | OddsRatio | ExpCount | Count | Size | Term |
| 05340 | 0.0005508745 | 108.1428571429 | 0 | 2 | 30 | Primary immunodeficiency |
| 04612 | 0.0017931763 | 57.7692307692 | 0 | 2 | 54 | Antigen processing and presentation |
| 04640 | 0.0019972952 | 54.5636363636 | 0 | 2 | 57 | Hematopoietic cell lineage |
| 04514 | 0.0044126993 | 35.8192771084 | 0 | 2 | 85 | Cell adhesion molecules (CAMs) |
| 04660 | 0.0047253134 | 34.5348837209 | 0 | 2 | 88 | T cell receptor signaling pathway |

  


Gene to ChrMap test for over-representation

| ChrMapID | Pvalue | OddsRatio | ExpCount | Count | Size |
| 2p12 | 0.0000200716 | 511.0625000000 | 0 | 2 | 10 |

  
  

# CGS 32 RPL37A n= 5

  
  
  


Gene to KEGG test for over-representation

| KEGGID | Pvalue | OddsRatio | ExpCount | Count | Size | Term |
| 03010 | 0.0070567205 | 24.1707317073 | 0 | 2 | 84 | Ribosome |

  
  
  

# CGS 33 PCOLCE n= 9

  


Gene to GO CC test for over-representation

| GOCCID | Pvalue | OddsRatio | ExpCount | Count | Size | Term |
| GO:0031012 | 0.0000421954 | 32.0666666667 | 0 | 4 | 184 | extracellular matrix |

  
  


Gene to KEGG test for over-representation

| KEGGID | Pvalue | OddsRatio | ExpCount | Count | Size | Term |
| 04510 | 0.0059641081 | 42.6277372263 | 0 | 2 | 139 | Focal adhesion |

  
  
  

# CGS 34 CD3E n= 10


Gene to GO BP test for over-representation

| GOBPID | Pvalue | OddsRatio | ExpCount | Count | Size | Term |
| GO:0045086 | 0.0000004768 | 328.1904761905 | 0 | 3 | 12 | positive regulation of interleukin-2 biosynthetic process |
| GO:0045076 | 0.0000006194 | 295.3285714286 | 0 | 3 | 13 | regulation of interleukin-2 biosynthetic process |
| GO:0042110 | 0.0000009409 | 47.5985915493 | 0 | 5 | 147 | T cell activation |
| GO:0042094 | 0.0000009839 | 246.0357142857 | 0 | 3 | 15 | interleukin-2 biosynthetic process |
| GO:0032663 | 0.0000028629 | 163.8809523810 | 0 | 3 | 21 | regulation of interleukin-2 production |
| GO:0002684 | 0.0000035361 | 35.9037433155 | 0 | 5 | 192 | positive regulation of immune system process |
| GO:0046649 | 0.0000048839 | 33.5050000000 | 0 | 5 | 205 | lymphocyte activation |
| GO:0032623 | 0.0000049358 | 134.0064935065 | 0 | 3 | 25 | interleukin-2 production |
| GO:0042102 | 0.0000096023 | 105.1989795918 | 0 | 3 | 31 | positive regulation of T cell proliferation |
| GO:0002376 | 0.0000112939 | 20.3779971791 | 1 | 7 | 716 | immune system process |
| GO:0045321 | 0.0000114792 | 27.8744769874 | 0 | 5 | 244 | leukocyte activation |
| GO:0001775 | 0.0000184785 | 25.1401515152 | 0 | 5 | 269 | cell activation |
| GO:0042108 | 0.0000209616 | 79.5057915058 | 0 | 3 | 40 | positive regulation of cytokine biosynthetic process |
| GO:0002682 | 0.0000261894 | 23.2992957746 | 0 | 5 | 289 | regulation of immune system process |
| GO:0001817 | 0.0000291206 | 33.6666666667 | 0 | 4 | 138 | regulation of cytokine production |
| GO:0042129 | 0.0000299912 | 69.9897959184 | 0 | 3 | 45 | regulation of T cell proliferation |
| GO:0050671 | 0.0000320592 | 68.3521594684 | 0 | 3 | 46 | positive regulation of lymphocyte proliferation |
| GO:0032946 | 0.0000342190 | 66.7889610390 | 0 | 3 | 47 | positive regulation of mononuclear cell proliferation |
| GO:0070665 | 0.0000342190 | 66.7889610390 | 0 | 3 | 47 | positive regulation of leukocyte proliferation |
| GO:0001816 | 0.0000437264 | 30.2102908277 | 0 | 4 | 153 | cytokine production |
| GO:0042035 | 0.0000520659 | 57.5630252101 | 0 | 3 | 54 | regulation of cytokine biosynthetic process |
| GO:0042089 | 0.0000645723 | 53.3454545455 | 0 | 3 | 58 | cytokine biosynthetic process |
| GO:0042107 | 0.0000679796 | 52.3852040816 | 0 | 3 | 59 | cytokine metabolic process |
| GO:0050776 | 0.0000692119 | 26.7182539683 | 0 | 4 | 172 | regulation of immune response |
| GO:0050870 | 0.0000789046 | 49.6997578692 | 0 | 3 | 62 | positive regulation of T cell activation |
| GO:0042098 | 0.0000827866 | 48.8642857143 | 0 | 3 | 63 | T cell proliferation |
| GO:0050670 | 0.0000867918 | 48.0562060890 | 0 | 3 | 64 | regulation of lymphocyte proliferation |
| GO:0032944 | 0.0000909220 | 47.2741935484 | 0 | 3 | 65 | regulation of mononuclear cell proliferation |
| GO:0070663 | 0.0000909220 | 47.2741935484 | 0 | 3 | 65 | regulation of leukocyte proliferation |
| GO:0030217 | 0.0001394874 | 40.6488095238 | 0 | 3 | 75 | T cell differentiation |
| GO:0051251 | 0.0001754408 | 37.4890109890 | 0 | 3 | 81 | positive regulation of lymphocyte activation |
| GO:0002696 | 0.0002169570 | 34.7806122449 | 0 | 3 | 87 | positive regulation of leukocyte activation |
| GO:0046651 | 0.0002320946 | 33.9617940199 | 0 | 3 | 89 | lymphocyte proliferation |
| GO:0050863 | 0.0002320946 | 33.9617940199 | 0 | 3 | 89 | regulation of T cell activation |
| GO:0032943 | 0.0002399143 | 33.5665024631 | 0 | 3 | 90 | mononuclear cell proliferation |
| GO:0070661 | 0.0002399143 | 33.5665024631 | 0 | 3 | 90 | leukocyte proliferation |
| GO:0050867 | 0.0002479036 | 33.1801948052 | 0 | 3 | 91 | positive regulation of cell activation |
| GO:0030098 | 0.0003374768 | 29.7507288630 | 0 | 3 | 101 | lymphocyte differentiation |
| GO:0050778 | 0.0004340493 | 27.2122830441 | 0 | 3 | 110 | positive regulation of immune response |
| GO:0051249 | 0.0004576831 | 26.7051114024 | 0 | 3 | 112 | regulation of lymphocyte activation |
| GO:0050794 | 0.0005704467 | Inf | 5 | 10 | 3277 | regulation of cellular process |
| GO:0002694 | 0.0006317478 | 23.8138173302 | 0 | 3 | 125 | regulation of leukocyte activation |
| GO:0050852 | 0.0006982232 | 66.1057692308 | 0 | 2 | 28 | T cell receptor signaling pathway |
| GO:0050865 | 0.0007574274 | 22.3219780220 | 0 | 3 | 133 | regulation of cell activation |
| GO:0042981 | 0.0008254082 | 10.6570945946 | 1 | 5 | 597 | regulation of apoptosis |
| GO:0002521 | 0.0008615105 | 21.3182773109 | 0 | 3 | 139 | leukocyte differentiation |
| GO:0043067 | 0.0008645273 | 10.5401337793 | 1 | 5 | 603 | regulation of programmed cell death |
| GO:0010941 | 0.0008913827 | 10.4634551495 | 1 | 5 | 607 | regulation of cell death |

  


Gene to GO CC test for over-representation

| GOCCID | Pvalue | OddsRatio | ExpCount | Count | Size | Term |
| GO:0001772 | 0.0000718383 | 234.4761904762 | 0 | 2 | 11 | immunological synapse |
| GO:0042101 | 0.0000718383 | 234.4761904762 | 0 | 2 | 11 | T cell receptor complex |
| GO:0009897 | 0.0001477574 | 41.0449438202 | 0 | 3 | 92 | external side of plasma membrane |

  


Gene to GO MF test for over-representation

| GOMFID | Pvalue | OddsRatio | ExpCount | Count | Size | Term |
| GO:0030159 | 0.0000912635 | 204.9714285714 | 0 | 2 | 12 | receptor signaling complex scaffold activity |
| GO:0032947 | 0.0002613669 | 113.7460317460 | 0 | 2 | 20 | protein complex scaffold |
| GO:0005070 | 0.0009060988 | 58.3591836735 | 0 | 2 | 37 | SH3/SH2 adaptor activity |

  


Gene to KEGG test for over-representation

| KEGGID | Pvalue | OddsRatio | ExpCount | Count | Size | Term |
| 04660 | 0.0000209413 | 47.1269841270 | 0 | 4 | 88 | T cell receptor signaling pathway |
| 04640 | 0.0067430202 | 21.8036363636 | 0 | 2 | 57 | Hematopoietic cell lineage |

  


Gene to ChrMap test for over-representation

| ChrMapID | Pvalue | OddsRatio | ExpCount | Count | Size |
| 10p15 | 0.0002031355 | 127.5781250000 | 0 | 2 | 18 |

  
  

# CGS 35 UBE2D3 n= 11


Gene to GO BP test for over-representation

| GOBPID | Pvalue | OddsRatio | ExpCount | Count | Size | Term |
| GO:0070979 | 0.0000024167 | 172.1250000000 | 0 | 3 | 18 | protein K11-linked ubiquitination |
| GO:0016567 | 0.0000068382 | 29.4298245614 | 0 | 5 | 195 | protein ubiquitination |
| GO:0006511 | 0.0000096055 | 27.3529411765 | 0 | 5 | 209 | ubiquitin-dependent protein catabolic process |
| GO:0019941 | 0.0000110316 | 26.5476190476 | 0 | 5 | 215 | modification-dependent protein catabolic process |
| GO:0032446 | 0.0000110316 | 26.5476190476 | 0 | 5 | 215 | protein modification by small protein conjugation |
| GO:0043632 | 0.0000110316 | 26.5476190476 | 0 | 5 | 215 | modification-dependent macromolecule catabolic process |
| GO:0070647 | 0.0000216910 | 22.9269972452 | 0 | 5 | 247 | protein modification by small protein conjugation or removal |
| GO:0051603 | 0.0000225574 | 22.7322404372 | 0 | 5 | 249 | proteolysis involved in cellular protein catabolic process |
| GO:0044257 | 0.0000230007 | 22.6360544218 | 0 | 5 | 250 | cellular protein catabolic process |
| GO:0030163 | 0.0000405036 | 20.0000000000 | 0 | 5 | 281 | protein catabolic process |
| GO:0044265 | 0.0001145619 | 15.8817829457 | 1 | 5 | 349 | cellular macromolecule catabolic process |
| GO:0044248 | 0.0001879486 | 11.8805687204 | 1 | 6 | 639 | cellular catabolic process |
| GO:0009057 | 0.0002293749 | 13.5776942356 | 1 | 5 | 404 | macromolecule catabolic process |
| GO:0051246 | 0.0003319135 | 12.4768518519 | 1 | 5 | 437 | regulation of protein metabolic process |
| GO:0006508 | 0.0003575909 | 12.2646165528 | 1 | 5 | 444 | proteolysis |
| GO:0070936 | 0.0004757995 | 80.4795321637 | 0 | 2 | 21 | protein K48-linked ubiquitination |
| GO:0009056 | 0.0005139891 | 9.6947368421 | 1 | 6 | 766 | catabolic process |
| GO:0000209 | 0.0007331646 | 63.6666666667 | 0 | 2 | 26 | protein polyubiquitination |
| GO:0043161 | 0.0009395811 | 20.1607142857 | 0 | 3 | 129 | proteasomal ubiquitin-dependent protein catabolic process |

  


Gene to GO CC test for over-representation

| GOCCID | Pvalue | OddsRatio | ExpCount | Count | Size | Term |
| GO:0000151 | 0.0001883197 | 35.6298701299 | 0 | 3 | 80 | ubiquitin ligase complex |

  


Gene to GO MF test for over-representation

| GOMFID | Pvalue | OddsRatio | ExpCount | Count | Size | Term |
| GO:0004842 | 0.0000003431 | 55.6289308176 | 0 | 5 | 111 | ubiquitin-protein ligase activity |
| GO:0019787 | 0.0000006470 | 48.6294765840 | 0 | 5 | 126 | small conjugating protein ligase activity |
| GO:0016881 | 0.0000012153 | 42.5362318841 | 0 | 5 | 143 | acid-amino acid ligase activity |
| GO:0016879 | 0.0000022563 | 37.2876857749 | 0 | 5 | 162 | ligase activity, forming carbon-nitrogen bonds |
| GO:0016874 | 0.0000212826 | 23.0112881806 | 0 | 5 | 256 | ligase activity |

  


Gene to KEGG test for over-representation

| KEGGID | Pvalue | OddsRatio | ExpCount | Count | Size | Term |
| 04120 | 0.0000015800 | 53.0286738351 | 0 | 5 | 98 | Ubiquitin mediated proteolysis |

  


Gene to ChrMap test for over-representation

| ChrMapID | Pvalue | OddsRatio | ExpCount | Count | Size |
| 4 | 0.0000000367 | 44.1314102564 | 0 | 7 | 319 |
| 4q | 0.0000002490 | 40.5702127660 | 0 | 6 | 241 |
| 4q2 | 0.0000120785 | 40.7939317320 | 0 | 4 | 117 |
| 4q21.1 | 0.0000895921 | 201.7530864198 | 0 | 2 | 11 |
| 4q24 | 0.0000895921 | 201.7530864198 | 0 | 2 | 11 |

  
  

# CGS 36 TRAM1 n= 6


Gene to GO BP test for over-representation

| GOBPID | Pvalue | OddsRatio | ExpCount | Count | Size | Term |
| GO:0006613 | 0.0000275611 | 459.7333333333 | 0 | 2 | 12 | cotranslational protein targeting to membrane |
| GO:0006612 | 0.0001807075 | 163.7619047619 | 0 | 2 | 30 | protein targeting to membrane |

  


Gene to GO CC test for over-representation

| GOCCID | Pvalue | OddsRatio | ExpCount | Count | Size | Term |
| GO:0012505 | 0.0000993354 | 39.4591346154 | 1 | 5 | 837 | endomembrane system |

  
  


Gene to KEGG test for over-representation

| KEGGID | Pvalue | OddsRatio | ExpCount | Count | Size | Term |
| 04141 | 0.0049887925 | 46.9120000000 | 0 | 2 | 127 | Protein processing in endoplasmic reticulum |

  
  
  

# CGS 37 HCK n= 2

  
  
  
  
  
  

# CGS 38 IGHM n= 2

  
  
  
  
  
  

# CGS 39 RPL39 n= 18


Gene to GO BP test for over-representation

| GOBPID | Pvalue | OddsRatio | ExpCount | Count | Size | Term |
| GO:0006414 | 0.0000000000 | 420.4938271605 | 0 | 15 | 96 | translational elongation |
| GO:0006412 | 0.0000000000 | 116.3556338028 | 1 | 15 | 299 | translation |
| GO:0044267 | 0.0000000024 | 28.9845741113 | 4 | 16 | 1507 | cellular protein metabolic process |
| GO:0019538 | 0.0000000182 | 24.3995299647 | 4 | 16 | 1718 | protein metabolic process |
| GO:0042274 | 0.0000000227 | 218.5396825397 | 0 | 4 | 13 | ribosomal small subunit biogenesis |
| GO:0034645 | 0.0000008474 | 13.8025095472 | 5 | 15 | 1848 | cellular macromolecule biosynthetic process |
| GO:0009059 | 0.0000011361 | 13.4107905983 | 5 | 15 | 1887 | macromolecule biosynthetic process |
| GO:0006364 | 0.0000011521 | 36.4369658120 | 0 | 5 | 77 | rRNA processing |
| GO:0016072 | 0.0000012291 | 35.9325605901 | 0 | 5 | 78 | rRNA metabolic process |
| GO:0010467 | 0.0000033759 | 12.0197530864 | 5 | 15 | 2040 | gene expression |
| GO:0042254 | 0.0000051385 | 26.3947163947 | 0 | 5 | 104 | ribosome biogenesis |
| GO:0044249 | 0.0000168583 | 10.1361440492 | 6 | 15 | 2292 | cellular biosynthetic process |
| GO:0034470 | 0.0000171524 | 20.3275240385 | 0 | 5 | 133 | ncRNA processing |
| GO:0009058 | 0.0000230458 | 9.7918454936 | 6 | 15 | 2345 | biosynthetic process |
| GO:0034660 | 0.0000359996 | 17.2897435897 | 0 | 5 | 155 | ncRNA metabolic process |
| GO:0022613 | 0.0000394819 | 16.9431875314 | 0 | 5 | 158 | ribonucleoprotein complex biogenesis |
| GO:0044260 | 0.0001643691 | 9.6404350608 | 8 | 16 | 3142 | cellular macromolecule metabolic process |
| GO:0006396 | 0.0004517733 | 7.9266503667 | 1 | 6 | 415 | RNA processing |
| GO:0043170 | 0.0005201566 | 8.2810746974 | 9 | 16 | 3403 | macromolecule metabolic process |

  


Gene to GO CC test for over-representation

| GOCCID | Pvalue | OddsRatio | ExpCount | Count | Size | Term |
| GO:0005840 | 0.0000000000 | 189.4179104478 | 0 | 14 | 148 | ribosome |
| GO:0022626 | 0.0000000000 | 248.3728813559 | 0 | 12 | 71 | cytosolic ribosome |
| GO:0033279 | 0.0000000000 | 169.7674418605 | 0 | 12 | 98 | ribosomal subunit |
| GO:0044445 | 0.0000000000 | 129.8928571429 | 0 | 12 | 124 | cytosolic part |
| GO:0030529 | 0.0000000000 | 100.5142857143 | 1 | 15 | 365 | ribonucleoprotein complex |
| GO:0022627 | 0.0000000000 | 218.0444444444 | 0 | 8 | 35 | cytosolic small ribosomal subunit |
| GO:0015935 | 0.0000000000 | 143.3170731707 | 0 | 8 | 49 | small ribosomal subunit |
| GO:0005829 | 0.0000000000 | 55.0608324440 | 2 | 16 | 953 | cytosol |
| GO:0043228 | 0.0000000001 | 62.5199493350 | 4 | 17 | 1596 | non-membrane-bounded organelle |
| GO:0043232 | 0.0000000001 | 62.5199493350 | 4 | 17 | 1596 | intracellular non-membrane-bounded organelle |
| GO:0032991 | 0.0000000019 | 47.8564049587 | 5 | 17 | 1953 | macromolecular complex |
| GO:0022625 | 0.0000010838 | 70.0571428571 | 0 | 4 | 34 | cytosolic large ribosomal subunit |
| GO:0044444 | 0.0000037053 | 23.8996742671 | 8 | 17 | 3087 | cytoplasmic part |
| GO:0015934 | 0.0000052532 | 45.5900621118 | 0 | 4 | 50 | large ribosomal subunit |
| GO:0044446 | 0.0000336342 | 11.6632279534 | 7 | 16 | 3021 | intracellular organelle part |
| GO:0044422 | 0.0000400723 | 11.4304505097 | 7 | 16 | 3057 | organelle part |
| GO:0005737 | 0.0001008201 | Inf | 11 | 18 | 4444 | cytoplasm |
| GO:0005730 | 0.0009916433 | 6.7554027505 | 1 | 6 | 515 | nucleolus |

  


Gene to GO MF test for over-representation

| GOMFID | Pvalue | OddsRatio | ExpCount | Count | Size | Term |
| GO:0003735 | 0.0000000000 | 226.8899082569 | 0 | 14 | 123 | structural constituent of ribosome |
| GO:0005198 | 0.0000000000 | 102.0895522388 | 1 | 15 | 350 | structural molecule activity |
| GO:0003723 | 0.0000119266 | 10.8904276986 | 1 | 8 | 499 | RNA binding |
| GO:0003676 | 0.0001959809 | 6.1353383459 | 4 | 11 | 1474 | nucleic acid binding |
| GO:0003729 | 0.0002542319 | 29.6958333333 | 0 | 3 | 51 | mRNA binding |
| GO:0019843 | 0.0009862917 | 52.6323529412 | 0 | 2 | 19 | rRNA binding |

  


Gene to KEGG test for over-representation

| KEGGID | Pvalue | OddsRatio | ExpCount | Count | Size | Term |
| 03010 | 0.0000000000 | 272.2676056338 | 0 | 13 | 84 | Ribosome |

  
  
  

# CGS 40 HLA-F n= 8


Gene to GO BP test for over-representation

| GOBPID | Pvalue | OddsRatio | ExpCount | Count | Size | Term |
| GO:0002474 | 0.0000000000 | Inf | 0 | 6 | 16 | antigen processing and presentation of peptide antigen via MHC class I |
| GO:0048002 | 0.0000000000 | Inf | 0 | 6 | 24 | antigen processing and presentation of peptide antigen |
| GO:0019882 | 0.0000000000 | Inf | 0 | 6 | 45 | antigen processing and presentation |
| GO:0006955 | 0.0000000985 | Inf | 0 | 6 | 472 | immune response |
| GO:0002376 | 0.0000012135 | Inf | 1 | 6 | 716 | immune system process |
| GO:0050896 | 0.0004093609 | Inf | 2 | 6 | 1885 | response to stimulus |

  


Gene to GO CC test for over-representation

| GOCCID | Pvalue | OddsRatio | ExpCount | Count | Size | Term |
| GO:0042612 | 0.0000000000 | Inf | 0 | 6 | 12 | MHC class I protein complex |
| GO:0042611 | 0.0000000000 | Inf | 0 | 6 | 21 | MHC protein complex |
| GO:0044459 | 0.0000036047 | Inf | 1 | 6 | 919 | plasma membrane part |
| GO:0005886 | 0.0000713878 | Inf | 1 | 6 | 1510 | plasma membrane |
| GO:0043234 | 0.0000944491 | Inf | 1 | 6 | 1582 | protein complex |
| GO:0016021 | 0.0002605114 | Inf | 2 | 6 | 1873 | integral to membrane |
| GO:0031224 | 0.0002866077 | Inf | 2 | 6 | 1903 | intrinsic to membrane |
| GO:0032991 | 0.0003349318 | Inf | 2 | 6 | 1953 | macromolecular complex |

  


Gene to GO MF test for over-representation

| GOMFID | Pvalue | OddsRatio | ExpCount | Count | Size | Term |
| GO:0004888 | 0.0000000066 | Inf | 0 | 6 | 314 | transmembrane receptor activity |
| GO:0004872 | 0.0000001236 | Inf | 0 | 6 | 510 | receptor activity |
| GO:0004871 | 0.0000014788 | Inf | 1 | 6 | 770 | signal transducer activity |
| GO:0060089 | 0.0000014788 | Inf | 1 | 6 | 770 | molecular transducer activity |

  


Gene to KEGG test for over-representation

| KEGGID | Pvalue | OddsRatio | ExpCount | Count | Size | Term |
| 05320 | 0.0000000000 | Inf | 0 | 6 | 26 | Autoimmune thyroid disease |
| 05330 | 0.0000000000 | Inf | 0 | 6 | 27 | Allograft rejection |
| 05332 | 0.0000000000 | Inf | 0 | 6 | 28 | Graft-versus-host disease |
| 04940 | 0.0000000000 | Inf | 0 | 6 | 32 | Type I diabetes mellitus |
| 05416 | 0.0000000000 | Inf | 0 | 6 | 51 | Viral myocarditis |
| 04612 | 0.0000000000 | Inf | 0 | 6 | 54 | Antigen processing and presentation |
| 04514 | 0.0000000004 | Inf | 0 | 6 | 85 | Cell adhesion molecules (CAMs) |
| 04145 | 0.0000000022 | Inf | 0 | 6 | 113 | Phagosome |
| 04144 | 0.0000000066 | Inf | 0 | 6 | 135 | Endocytosis |
| 04650 | 0.0000000557 | 204.1780821918 | 0 | 5 | 78 | Natural killer cell mediated cytotoxicity |

  


Gene to ChrMap test for over-representation

| ChrMapID | Pvalue | OddsRatio | ExpCount | Count | Size |
| 6p21.3 | 0.0000000000 | Inf | 0 | 7 | 119 |
| 6p21 | 0.0000000000 | Inf | 0 | 7 | 172 |
| 6p2 | 0.0000000000 | Inf | 0 | 7 | 252 |
| 6p | 0.0000000001 | Inf | 0 | 7 | 287 |
| 6 | 0.0000000022 | Inf | 0 | 7 | 478 |

  
  

# CGS 41 GOLGA8A n= 2

  


Gene to GO CC test for over-representation

| GOCCID | Pvalue | OddsRatio | ExpCount | Count | Size | Term |
| GO:0032580 | 0.0000284610 | Inf | 0 | 2 | 40 | Golgi cisterna membrane |
| GO:0031985 | 0.0000394441 | Inf | 0 | 2 | 47 | Golgi cisterna |
| GO:0031984 | 0.0000429105 | Inf | 0 | 2 | 49 | organelle subcompartment |
| GO:0005795 | 0.0000645846 | Inf | 0 | 2 | 60 | Golgi stack |

  
  
  


Gene to ChrMap test for over-representation

| ChrMapID | Pvalue | OddsRatio | ExpCount | Count | Size |
| 15q1 | 0.0000395324 | Inf | 0 | 2 | 52 |
| 15 | 0.0008693839 | Inf | 0 | 2 | 242 |
| 15q | 0.0008693839 | Inf | 0 | 2 | 242 |

  
  

# CGS 42 CD164 n= 6

  
  
  
  


Gene to ChrMap test for over-representation

| ChrMapID | Pvalue | OddsRatio | ExpCount | Count | Size |
| 6q21 | 0.0000000016 | 680.0833333333 | 0 | 4 | 28 |
| 6q | 0.0000000346 | 219.8626373626 | 0 | 5 | 187 |
| 6q2 | 0.0000009430 | 125.8906250000 | 0 | 4 | 132 |
| 6 | 0.0000037896 | 81.5221987315 | 0 | 5 | 478 |

  
  

# CGS 43 VCAM1 n= 3

  
  
  
  
  
  

# CGS 44 IFITM1 n= 3


Gene to GO BP test for over-representation

| GOBPID | Pvalue | OddsRatio | ExpCount | Count | Size | Term |
| GO:0009615 | 0.0000039247 | Inf | 0 | 3 | 110 | response to virus |
| GO:0045087 | 0.0000062080 | Inf | 0 | 3 | 128 | innate immune response |
| GO:0051707 | 0.0000429541 | Inf | 0 | 3 | 243 | response to other organism |
| GO:0009607 | 0.0000794018 | Inf | 0 | 3 | 298 | response to biotic stimulus |
| GO:0006952 | 0.0002343108 | Inf | 0 | 3 | 427 | defense response |
| GO:0006955 | 0.0003166844 | Inf | 0 | 3 | 472 | immune response |
| GO:0051704 | 0.0004799060 | Inf | 0 | 3 | 542 | multi-organism process |

  
  
  
  


Gene to ChrMap test for over-representation

| ChrMapID | Pvalue | OddsRatio | ExpCount | Count | Size |
| 11p15.5 | 0.0000001079 | Inf | 0 | 3 | 40 |
| 11p15 | 0.0000018201 | Inf | 0 | 3 | 101 |
| 11p1 | 0.0000077389 | Inf | 0 | 3 | 163 |
| 11p | 0.0000078831 | Inf | 0 | 3 | 164 |
| 11 | 0.0001865881 | Inf | 0 | 3 | 469 |

  
  

# CGS 45 PSMB3 n= 9


Gene to GO BP test for over-representation

| GOBPID | Pvalue | OddsRatio | ExpCount | Count | Size | Term |
| GO:0043161 | 0.0000135807 | 43.3728000000 | 0 | 4 | 129 | proteasomal ubiquitin-dependent protein catabolic process |
| GO:0010498 | 0.0000148786 | 42.3375000000 | 0 | 4 | 132 | proteasomal protein catabolic process |
| GO:0051436 | 0.0000409037 | 64.6132075472 | 0 | 3 | 56 | negative regulation of ubiquitin-protein ligase activity involved in mitotic cell cycle |
| GO:0031145 | 0.0000454716 | 62.2454545455 | 0 | 3 | 58 | anaphase-promoting complex-dependent proteasomal ubiquitin-dependent protein catabolic process |
| GO:0051437 | 0.0000454716 | 62.2454545455 | 0 | 3 | 58 | positive regulation of ubiquitin-protein ligase activity involved in mitotic cell cycle |
| GO:0051352 | 0.0000503631 | 60.0438596491 | 0 | 3 | 60 | negative regulation of ligase activity |
| GO:0051444 | 0.0000503631 | 60.0438596491 | 0 | 3 | 60 | negative regulation of ubiquitin-protein ligase activity |
| GO:0051439 | 0.0000529335 | 59.0000000000 | 0 | 3 | 61 | regulation of ubiquitin-protein ligase activity involved in mitotic cell cycle |
| GO:0051443 | 0.0000611585 | 56.0737704918 | 0 | 3 | 64 | positive regulation of ubiquitin-protein ligase activity |
| GO:0051351 | 0.0000670832 | 54.2777777778 | 0 | 3 | 66 | positive regulation of ligase activity |
| GO:0016567 | 0.0000693079 | 28.1089005236 | 0 | 4 | 195 | protein ubiquitination |
| GO:0031397 | 0.0000800378 | 51.0074626866 | 0 | 3 | 70 | negative regulation of protein ubiquitination |
| GO:0051438 | 0.0000870882 | 49.5144927536 | 0 | 3 | 72 | regulation of ubiquitin-protein ligase activity |
| GO:0006511 | 0.0000908960 | 26.1346341463 | 0 | 4 | 209 | ubiquitin-dependent protein catabolic process |
| GO:0051340 | 0.0000945341 | 48.1056338028 | 0 | 3 | 74 | regulation of ligase activity |
| GO:0019941 | 0.0001015138 | 25.3687203791 | 0 | 4 | 215 | modification-dependent protein catabolic process |
| GO:0032446 | 0.0001015138 | 25.3687203791 | 0 | 4 | 215 | protein modification by small protein conjugation |
| GO:0043632 | 0.0001015138 | 25.3687203791 | 0 | 4 | 215 | modification-dependent macromolecule catabolic process |
| GO:0031398 | 0.0001193455 | 44.3181818182 | 0 | 3 | 80 | positive regulation of protein ubiquitination |
| GO:0070647 | 0.0001741471 | 21.9226337449 | 0 | 4 | 247 | protein modification by small protein conjugation or removal |
| GO:0051603 | 0.0001796786 | 21.7371428571 | 0 | 4 | 249 | proteolysis involved in cellular protein catabolic process |
| GO:0044257 | 0.0001824919 | 21.6455284553 | 0 | 4 | 250 | cellular protein catabolic process |
| GO:0031396 | 0.0002250178 | 35.4479166667 | 0 | 3 | 99 | regulation of protein ubiquitination |
| GO:0030163 | 0.0002867281 | 19.1335740072 | 0 | 4 | 281 | protein catabolic process |
| GO:0031400 | 0.0003595817 | 30.0398230088 | 0 | 3 | 116 | negative regulation of protein modification process |
| GO:0044265 | 0.0006578654 | 15.2046376812 | 0 | 4 | 349 | cellular macromolecule catabolic process |
| GO:0032269 | 0.0009232904 | 21.4808917197 | 0 | 3 | 160 | negative regulation of cellular protein metabolic process |
| GO:0031401 | 0.0009746169 | 21.0687500000 | 0 | 3 | 163 | positive regulation of protein modification process |

  


Gene to GO CC test for over-representation

| GOCCID | Pvalue | OddsRatio | ExpCount | Count | Size | Term |
| GO:0000502 | 0.0000370674 | 66.7272727273 | 0 | 3 | 58 | proteasome complex |
| GO:0005839 | 0.0001989605 | 131.7678571429 | 0 | 2 | 18 | proteasome core complex |

  


Gene to GO MF test for over-representation

| GOMFID | Pvalue | OddsRatio | ExpCount | Count | Size | Term |
| GO:0015078 | 0.0000447335 | 62.5175438596 | 0 | 3 | 60 | hydrogen ion transmembrane transporter activity |
| GO:0015077 | 0.0001100697 | 45.5512820513 | 0 | 3 | 81 | monovalent inorganic cation transmembrane transporter activity |
| GO:0004129 | 0.0001449094 | 157.6043956044 | 0 | 2 | 15 | cytochrome-c oxidase activity |
| GO:0015002 | 0.0001449094 | 157.6043956044 | 0 | 2 | 15 | heme-copper terminal oxidase activity |
| GO:0016675 | 0.0001449094 | 157.6043956044 | 0 | 2 | 15 | oxidoreductase activity, acting on heme group of donors |
| GO:0016676 | 0.0001449094 | 157.6043956044 | 0 | 2 | 15 | oxidoreductase activity, acting on heme group of donors, oxygen as acceptor |
| GO:0004298 | 0.0001874485 | 136.5523809524 | 0 | 2 | 17 | threonine-type endopeptidase activity |
| GO:0070003 | 0.0001874485 | 136.5523809524 | 0 | 2 | 17 | threonine-type peptidase activity |
| GO:0022890 | 0.0002250121 | 35.4200000000 | 0 | 3 | 103 | inorganic cation transmembrane transporter activity |

  


Gene to KEGG test for over-representation

| KEGGID | Pvalue | OddsRatio | ExpCount | Count | Size | Term |
| 03050 | 0.0001908120 | 38.6153846154 | 0 | 3 | 42 | Proteasome |
| 05012 | 0.0020620260 | 16.2637362637 | 0 | 3 | 94 | Parkinson's disease |
| 00190 | 0.0021913898 | 15.9032258065 | 0 | 3 | 96 | Oxidative phosphorylation |
| 05010 | 0.0048809116 | 11.8024193548 | 0 | 3 | 127 | Alzheimer's disease |
| 04260 | 0.0051178506 | 23.9285714286 | 0 | 2 | 38 | Cardiac muscle contraction |
| 05016 | 0.0059226608 | 10.9699248120 | 0 | 3 | 136 | Huntington's disease |

  


Gene to ChrMap test for over-representation

| ChrMapID | Pvalue | OddsRatio | ExpCount | Count | Size |
| 22q13.2 | 0.0001277693 | 166.6938775510 | 0 | 2 | 16 |

  
  

# CGS 46 SSBP1 n= 7

  
  
  
  


Gene to ChrMap test for over-representation

| ChrMapID | Pvalue | OddsRatio | ExpCount | Count | Size |
| 7 | 0.0000040712 | 52.0600000000 | 0 | 5 | 380 |
| 7q3 | 0.0000793166 | 56.6144859813 | 0 | 3 | 110 |
| 7q | 0.0008977194 | 24.1002024291 | 0 | 3 | 250 |

  
  

# CGS 47 ENPP2 n= 2


Gene to GO BP test for over-representation

| GOBPID | Pvalue | OddsRatio | ExpCount | Count | Size | Term |
| GO:0016042 | 0.0002114961 | Inf | 0 | 2 | 101 | lipid catabolic process |

  
  
  


Gene to KEGG test for over-representation

| KEGGID | Pvalue | OddsRatio | ExpCount | Count | Size | Term |
| 00565 | 0.0000290582 | Inf | 0 | 2 | 17 | Ether lipid metabolism |
| 00591 | 0.0078290355 | 277.0000000000 | 0 | 1 | 12 | Linoleic acid metabolism |

  
  
  

# CGS 48 CDC6 n= 7


Gene to GO BP test for over-representation

| GOBPID | Pvalue | OddsRatio | ExpCount | Count | Size | Term |
| GO:0006261 | 0.0000000010 | 300.3070175439 | 0 | 5 | 62 | DNA-dependent DNA replication |
| GO:0006270 | 0.0000000022 | 540.1568627451 | 0 | 4 | 21 | DNA-dependent DNA replication initiation |
| GO:0006260 | 0.0000001616 | 103.3895705521 | 0 | 5 | 168 | DNA replication |
| GO:0006259 | 0.0000002173 | 101.0387596899 | 0 | 6 | 393 | DNA metabolic process |
| GO:0031570 | 0.0000107951 | 114.3166666667 | 0 | 3 | 48 | DNA integrity checkpoint |
| GO:0000075 | 0.0000673040 | 60.1676470588 | 0 | 3 | 88 | cell cycle checkpoint |
| GO:0051052 | 0.0000744122 | 58.0909090909 | 0 | 3 | 91 | regulation of DNA metabolic process |
| GO:0090329 | 0.0000795712 | 229.7333333333 | 0 | 2 | 14 | regulation of DNA-dependent DNA replication |
| GO:0008156 | 0.0003049965 | 110.0640000000 | 0 | 2 | 27 | negative regulation of DNA replication |
| GO:0051053 | 0.0005450539 | 80.8235294118 | 0 | 2 | 36 | negative regulation of DNA metabolic process |

  


Gene to GO CC test for over-representation

| GOCCID | Pvalue | OddsRatio | ExpCount | Count | Size | Term |
| GO:0031981 | 0.0000011960 | Inf | 1 | 7 | 1058 | nuclear lumen |
| GO:0005654 | 0.0000036013 | 60.4401197605 | 1 | 6 | 674 | nucleoplasm |
| GO:0070013 | 0.0000045780 | Inf | 1 | 7 | 1281 | intracellular organelle lumen |
| GO:0043233 | 0.0000051590 | Inf | 1 | 7 | 1303 | organelle lumen |
| GO:0031974 | 0.0000059261 | Inf | 1 | 7 | 1329 | membrane-enclosed lumen |
| GO:0044428 | 0.0000063782 | Inf | 1 | 7 | 1343 | nuclear part |
| GO:0015630 | 0.0001693338 | 26.4488262911 | 0 | 4 | 359 | microtubule cytoskeleton |
| GO:0043228 | 0.0005686745 | 21.9132075472 | 2 | 6 | 1596 | non-membrane-bounded organelle |
| GO:0043232 | 0.0005686745 | 21.9132075472 | 2 | 6 | 1596 | intracellular non-membrane-bounded organelle |
| GO:0044430 | 0.0006487000 | 18.2354497354 | 0 | 4 | 508 | cytoskeletal part |

  
  


Gene to KEGG test for over-representation

| KEGGID | Pvalue | OddsRatio | ExpCount | Count | Size | Term |
| 04110 | 0.0000186403 | 58.4752475248 | 0 | 4 | 105 | Cell cycle |
| 03030 | 0.0015485068 | 50.4000000000 | 0 | 2 | 32 | DNA replication |

  
  
  

# CGS 49 CD72 n= 2

  
  
  
  


Gene to ChrMap test for over-representation

| ChrMapID | Pvalue | OddsRatio | ExpCount | Count | Size |
| 9p1 | 0.0000256692 | Inf | 0 | 2 | 42 |
| 9p | 0.0001089674 | Inf | 0 | 2 | 86 |

  
  

# CGS 50 IGLL3 n= 2

  
  
  
  


Gene to ChrMap test for over-representation

| ChrMapID | Pvalue | OddsRatio | ExpCount | Count | Size |
| 22q11.23 | 0.0000027130 | Inf | 0 | 2 | 14 |
| 22q11.2 | 0.0000282033 | Inf | 0 | 2 | 44 |
| 22q11 | 0.0000380119 | Inf | 0 | 2 | 51 |
| 22q1 | 0.0005240571 | Inf | 0 | 2 | 188 |
| 22q | 0.0005296620 | Inf | 0 | 2 | 189 |
| 22 | 0.0005409612 | Inf | 0 | 2 | 191 |

  
