# Supplementary material for: Massive Transcriptional Perturbation in Subgroups of Diffuse Large B-Cell Lymphomas
Source: PLoS One. 2013 Nov 4;8(11):e76287. doi: 10.1371/journal.pone.0076287 (PMC3817189; doi:10.1371/journal.pone.0076287)
Supplement: Table S1 — Incidence of biologic features in the CAPs. (PDF) [file pone.0076287.s019.pdf]

**Table S1**. Incidence of biologic features in the CAPs.							
	**HiGA-PRO**		**HiGA-SIR**		**LoGA**		**p-value**
	N = 106		N = 189		N = 69		
**Morphology**	No.	%	No.	%	No.	%	
centroblastic	59	56%	118	62%	37	54%	
immunoblastic	5	5%	5	3%	6	9%	
plasmoblastic	8	8%	7	4%	4	6%	0.116
NA	34	32%	59	31%	22	32%	
**Immunoblastic morphology**							
neg	59	56%	118	62%	37	54%	
pos	5	5%	5	3%	6	9%	0.079
NA	42	40%	66	35%	26	38%	
**CD10 expression**							
neg	72	68%	121	64%	36	52%	
pos	26	25%	47	25%	24	35%	0.165
NA	8	8%	21	11%	9	13%	
**CD5 expression**							
neg	92	87%	136	72%	51	74%	
pos	4	4%	26	14%	5	7%	0.009
NA	10	9%	27	14%	13	19%	
**BCL2 expression**							
neg	14	13%	36	19%	13	19%	
pos	87	82%	126	67%	50	72%	0.231
NA	5	5%	27	14%	6	9%	
**BCL6 expression**							
neg	14	13%	37	20%	9	13%	
pos	79	75%	123	65%	50	72%	0.219
NA	13	12%	29	15%	10	14%	
**IRF4/MUM1 expression**							
neg	17	16%	57	30%	15	22%	
pos	80	75%	102	54%	38	55%	0.006
NA	9	8%	30	16%	16	23%	
**KI67 ≥ 90%**							
neg	65	61%	133	70%	44	64%	
pos	34	32%	39	21%	19	28%	0.094
NA	7	7%	17	9%	6	9%	
**GENE EXPRESSION**							
**GCB/ABC GEP**							
ABC	43	41%	46	24%	17	25%	
GCB	34	32%	100	53%	43	62%	<0.001
NA	29	27%	43	23%	9	13%	
**Consensus Cluster**							
BCR	46	43%	4	2%	25	36%	
HR	7	7%	29	15%	19	28%	
OxPhos	10	9%	10	5%	0	0%	<0.001
NA	43	41%	146	77%	25	36%	
**PAP (details)**							
mind-L	30	28%	28	15%	25	36%	
PAP-1	19	18%	107	57%	25	36%	
PAP-2	17	16%	4	2%	18	26%	
PAP-3	26	25%	22	12%	0	0%	
PAP-4	14	13%	28	15%	1	1%	<0.001
**PAP1 GEP**							
neg	87	82%	82	43%	44	64%	
pos	19	18%	107	57%	25	36%	<0.001
**GENETICS**							
**MYC-break**							
neg	90	85%	175	93%	56	81%	
pos	13	12%	10	5%	11	16%	0.012

NA	3	3%	4	2%	2	3%	
**MYC-status (details)**							
IG-MYC	2	2%	5	3%	4	6%	
neg	90	85%	175	93%	56	81%	
non-IG-MYC	11	10%	5	3%	7	10%	0.011
NA	3	3%	4	2%	2	3%	
**IGH-BCL2 fusion**							
neg	95	90%	163	86%	52	75%	
pos	9	8%	22	12%	16	23%	0.019
NA	2	2%	4	2%	1	1%	
**BCL6-break**							
neg	71	67%	134	71%	55	80%	
pos	28	26%	51	27%	14	20%	0.442
NA	7	7%	4	2%	0	0%	
**IGH-break**							
neg	50	47%	62	33%	39	57%	
pos	23	22%	23	12%	12	17%	0.628
NA	33	31%	104	55%	18	26%	
**IRF4-break**							
neg	40	38%	69	37%	27	39%	
pos	4	4%	2	1%	1	1%	0.344
NA	62	58%	118	62%	41	59%	
**EZH2 mutation**							
neg	94	89%	165	87%	49	71%	
pos	5	5%	13	7%	9	13%	0.068
NA	7	7%	11	6%	11	16%	
**+1q21**							
neg	68	64%	130	69%	29	42%	
pos	19	18%	15	8%	12	17%	0.004
NA	19	18%	44	23%	28	41%	
**+2p16**							
neg	68	64%	122	65%	24	35%	
pos	19	18%	23	12%	17	25%	0.003
NA	19	18%	44	23%	28	41%	
**+3q27**							
neg	64	60%	126	67%	30	43%	
pos	23	22%	19	10%	11	16%	0.018
NA	19	18%	44	23%	28	41%	
**+6p25**							
neg	73	69%	115	61%	32	46%	
pos	14	13%	30	16%	9	13%	0.616
NA	19	18%	44	23%	28	41%	
**+6p22**							
neg	76	72%	119	63%	31	45%	
pos	11	10%	26	14%	10	14%	0.251
NA	19	18%	44	23%	28	41%	
**+7p22**							
neg	68	64%	130	69%	27	39%	
pos	19	18%	15	8%	14	20%	0.001
NA	19	18%	44	23%	28	41%	
**+7q21**							
neg	66	62%	129	68%	30	43%	
pos	21	20%	16	8%	11	16%	0.008
NA	19	18%	44	23%	28	41%	
**+8q24**							
neg	79	75%	135	71%	33	48%	
pos	8	8%	10	5%	8	12%	0.064
NA	19	18%	44	23%	28	41%	
**+9q33**							

neg	79	75%	134	71%	35	51%	
pos	8	8%	11	6%	6	9%	0.401
NA	19	18%	44	23%	28	41%	
**+11q12**							
neg	72	68%	128	68%	35	51%	
pos	15	14%	17	9%	6	9%	0.487
NA	19	18%	44	23%	28	41%	
**+11q23.1**							
neg	66	62%	129	68%	35	51%	
pos	21	20%	16	8%	6	9%	0.033
NA	19	18%	44	23%	28	41%	
**+11q23.3**							
neg	60	57%	118	62%	31	45%	
pos	27	25%	27	14%	10	14%	0.094
NA	19	18%	44	23%	28	41%	
**+12q13**							
neg	69	65%	122	65%	34	49%	
pos	18	17%	23	12%	7	10%	0.64
NA	19	18%	44	23%	28	41%	
**+18q21**							
neg	51	48%	118	62%	30	43%	
pos	36	34%	27	14%	11	16%	<0.001
NA	19	18%	44	23%	28	41%	
**+19q13**							
neg	70	66%	134	71%	37	54%	
pos	17	16%	11	6%	4	6%	0.026
NA	19	18%	44	23%	28	41%	
**+Xq21**							
neg	69	65%	111	59%	31	45%	
pos	14	13%	27	14%	7	10%	0.904
NA	23	22%	51	27%	31	45%	
**-6q21**							
neg	51	48%	118	62%	30	43%	
pos	36	34%	27	14%	11	16%	<0.001
NA	19	18%	44	23%	28	41%	
**-6q23**							
neg	55	52%	118	62%	33	48%	
pos	32	30%	27	14%	8	12%	0.007
NA	19	18%	44	23%	28	41%	
**-8p23**							
neg	73	69%	135	71%	34	49%	
pos	14	13%	10	5%	7	10%	0.039
NA	19	18%	44	23%	28	41%	
**-9p21**							
neg	70	66%	131	69%	31	45%	
pos	17	16%	14	7%	10	14%	0.02
NA	19	18%	44	23%	28	41%	
**-17p13**							
neg	66	62%	137	72%	31	45%	
pos	21	20%	8	4%	10	14%	<0.001
NA	19	18%	44	23%	28	41%	
**-Xp22**							
neg	76	72%	126	67%	37	54%	
pos	7	7%	12	6%	1	1%	0.553
NA	23	22%	51	27%	31	45%	
**Age > 45**							
neg	14	13%	45	24%	10	14%	
pos	92	87%	144	76%	59	86%	0.053
**Age > 60**							

neg	34	32%	90	48%	33	48%	
pos	72	68%	99	52%	36	52%	0.023
**HISTONE MODIFICATION**							
**H3K4me2**							
neg	0	0%	0	0%	1	1%	
pos	25	24%	57	30%	20	29%	
NA	81	76%	132	70%	48	70%	0.204
**H3K27me3**							
neg	0	0%	1	1%	0	0%	
pos	8	8%	19	10%	5	7%	
NA	98	92%	169	89%	64	93%	1
**H3K18ac**							
neg	0	0%	2	1%	3	4%	
pos	38	36%	60	32%	23	33%	
NA	68	64%	127	67%	43	62%	0.074

The p-values were computed using the Fisher's exact test. GCB/ABC: germinal center B-cell, activated B-cell lymphoma gene expression signatures; GEP: gene expression profiling; PAP ("pathway activation pattern"): classification according to Bentink et al (2008). The categories of the data on histone modifications are defined by 0% = negative, >0% = positive. See File S2 for the definitions of the categories of the other characteristics.
