# Supplementary material for: Massive Transcriptional Perturbation in Subgroups of Diffuse Large B-Cell Lymphomas
Source: PLoS One. 2013 Nov 4;8(11):e76287. doi: 10.1371/journal.pone.0076287 (PMC3817189; doi:10.1371/journal.pone.0076287)
Supplement: Text S1 — Supporting material. (PDF) [file pone.0076287.s020.pdf]

Supporting Information

Massive transcriptional perturbation in subgroups of diffuse large B-cell lymphomas

Maciej Rosolowski[1,*], Jürgen Läuter[1,2], Dmitriy Abramov[3,4], Hans G. Drexler[5], Michael Hummel[6], Wolfram Klapper[3], Roderick A.F. MacLeod[5], Shoji Pellissery[7], Friedemann Horn[8], Reiner Siebert[7], Markus Loeffler[1]

[1]Institute of Medical Informatics, Statistics and Epidemiology (IMISE), University of Leipzig, Leipzig, Germany; [2]Otto von Guericke University Magdeburg, Magdeburg, Germany; [3]Department of Pathology, Hematopathology Section and Lymph Node Registry, University of Kiel, Kiel, Germany; [4]Department of Pathology, Russian Federal Research Center, Moscow, Russia; [5]Department of Human and Animal Cell Lines, DSMZ - German Collection of Microorganisms and Cell Cultures, Inhoffenstr. 7B, 38124 Braunschweig, Germany; [6]Institute of Pathology, Campus Benjamin Franklin, Charité-Universitätsmedizin Berlin, Germany; [7]Institute of Human Genetics, University Hospital Schleswig-Holstein Campus Kiel/University of Kiel, Kiel, Germany; [8]Institute of Clinical Immunology, Medical Faculty, University of Leipzig, Leipzig, Germany

* Corresponding author. Dr. Maciej Rosolowski, Institute of Medical Informatics, Statistics and Epidemiology (IMISE), University of Leipzig, Härtelstr. 16-18, 04107 Leipzig, Germany. E-mail: maciej.rosolowski@imise.uni-leipzig.de, Telephone: +49 341 97 16 177, Fax: +49 341 97 16 109.

Table of contents

1. Members of the Network Project of the Deutsche Krebshilfe "Molecular Mechanisms in Malignant Lymphomas" (Alphabetical order) 2
2. Data preparation..... 3
3. Generation of correlated gene sets (CGSs)..... 4
4. Summaries of the CGSs..... 5
5. Unsupervised analysis using principal components of the CGSs..... 5
6. Validation of the CGSA method on two published BL/DLBCL data sets 6
   a) Testing whether the unsupervised ordering of samples in the heatmaps reproduces known molecular subtypes of B-cell lymphomas..... 7
   b) Rubustness of the results of the unsupervised analysis with respect to the number of the CGSs..... 7
   c) Rubustness of the results of the unsupervised analysis to the sampling error within one data set..... 7
   d) Reproducibility of the results of the unsupervised analysis across data sets..... 8
7. Testing for the association between the CGSs and other biologic features of the patients 8
8. Validation of the CAPs in an independent data set..... 9
9. Analysis of differential expression 10
10. LE and HE genes.....10
11. Kernel density estimation.....10
12. Pathway analysis with PAGE (Pathway Analysis of Gene Expression).....10
13. Discussion of the CGSA method and its relation to other approaches 10
14. References 11

1. Members of the Network Project of the Deutsche Krebshilfe "Molecular Mechanisms in Malignant Lymphomas" (Alphabetical order)

Pathology group: Thomas F.E. Barth[1], Heinz-Wolfram Bernd[2], Sergio B. Cogliatti[3], Alfred C. Feller[2], Martin L. Hansmann[4], Michael Hummel[5], Wolfram Klapper[6], Peter Möller[1], Hans-Konrad Müller-Hermelink[7], Ilske Oschlies[6], German Ott[20], Andreas Rosenwald[7], Harald Stein[5], Monika Szczepanowski[6], Hans-Heinrich Wacker[6]. **Genetics group:** Thomas F.E. Barth[1], Petra Behrmann[8], Peter Daniel[9], Judith Dierlamm[8], Stefan Gesk,[10] Eugenia Haralambieva[7], Lana Harder[10], Paul-Martin Holterhus[11], Ralf Küppers[12], Dieter Kube[13], Peter Lichter[14], Jose I. Martín-Subero[10], Peter Möller[1], Eva M. Murga-Peñas[8], German Ott[20], Claudia Philipp[12], Christiane Pott[15], Armin Pscherer[14], Julia Richter[10], Andreas Rosenwald[7], Itziar Salaverria[10],Carsten Schwaenen[16], Reiner Siebert[10], Heiko Trautmann[15], Martina Vockerodt[17], Swen Wessendorf[16], **Bioinformatics group:** Stefan Bentink[18], Hilmar Berger[19], Christian W Kohler[18], Dirk Hasenclever[19], Markus Kreuz[19], Markus Loeffler[19], Maciej Rosolowski[19], Rainer Spang[18]. **Project coordination:** Benjamin Stürzenhofecker[13], Lorenz Trümper[13], Maren Wehner[13]. **Steering committee:** Markus Loeffler[19], Reiner Siebert[10], Harald Stein[5], Lorenz Trümper[13].

[1]Institute of Pathology, University Hospital of Ulm, Germany, [2]Institute of Pathology, University Hospital Schleswig-Holstein Campus Lübeck, Germany, [3]Institute of Pathology, Kantonsspital St. Gallen, Switzerland, [4]Institute of Pathology, University Hospital of Frankfurt,

Germany, [5] Institute of Pathology, Campus Benjamin Franklin, Charité–Universitätsmedizin Berlin, Germany, [6] Institute of Hematopathology, University Hospital Schleswig-Holstein Campus Kiel/ Christian-Albrechts University Kiel, Germany, [7]Institute of Pathology, University of Würzburg, Germany, [8] University Medical Center Hamburg-Eppendorf, Hamburg, Germany, [9]Department of Hematology, Oncology and Tumor Immunology, University Medical Center Charité, Germany, [10] Institute of Human Genetics, University Hospital Schleswig-Holstein Campus Kiel/Christian-Albrechts University Kiel, Germany, [11]Division of Pediatric Endocrinology and Diabetes, Department of Pediatrics, University Hospital Schleswig-Holstein Campus Kiel / Christian-Albrechts University Kiel, Germany, [12]Institute for Cell Biology (Tumor Research), University of Duisburg-Essen, Germany, [13]Department of Hematology and Oncology, Georg-August University of Göttingen, Germany, [14]German Cancer Research Center (DKFZ), Heidelberg, Germany, [15] Second Medical Department, University Hospital Schleswig-Holstein Campus Kiel/ Christian-Albrechts University Kiel, Germany, [16] Cytogenetic and Molecular Diagnostics, Internal Medicine III, University Hospital of Ulm, Germany, [17]Department of Pediatrics I, Georg-August University of Göttingen, Germany, [18]Institute of Functional Genomics, University of Regensburg, Germany, [19]Institute for Medical Informatics, Statistics and Epidemiology, University of Leipzig, Germany [20] Institute of Clinical Pathology, Robert-Bosch-Krankenhaus, Stuttgart, Germany.

2. Data preparation

Prior to generating correlated gene sets (CGSs), the data sets were prepared to remove noisy measurements and to deal with multiple probes per gene which were additionally highly correlated. The aim of the data preparation was to eliminate noisy probesets and strong but uninteresting correlations among probesets.

In the BL/DLBCL data set of Hummel et al. (2006) [1] (accesion number GSE4475), probesets without Entrez ID or with variance below 0.05 were excluded. Then, the following procedure was applied to each group of probe sets having the same Entrez ID:

1. Select a probe set with the highest variance.
2. Find probe sets which correlate well with the probe set selected in step 1 (Pearson's correlation coefficient greater or equal 0.7).
3. Aggregate the selected probe sets by computing their average. From now on the average is a new "probe set" represented by the name of the probe set selected in step 1.
4. Go to 1 and repeat the procedure on the remaining probe sets until all probe sets are processed.

The procedure resulted in 9580 from the initial 22283 features.

In the BL/DLBCL data set of Dave et al. (2006) [2] (accession number GSE4732), we used gene symbols instead of Entrez IDs to identify multiple probes which measure expression of the same gene. Moreover, we did not apply the initial variance filter since the used platform (LymphDx) was customized for lymphoma. The 2745 probes in the original data set were aggregated with the above procedure to 2460 probes.

In the data set of 364 DLBCL and related mature aggressive B-cell lymphomas ("extended DLBCL data set", accession numbers GSE4475, GSE10172, GSE22470) which was used in

the main part of this study, probe sets were selected using the same procedure as the one applied to the BL/DLBCL data set of Hummel et al. (2006). As a result, 9473 probe sets remained in the data set.

The labels of consensus clusters in the BL/DLBCL data set of Hummel et al. (2006) were obtained from Stefan Bentink. He transferred them from the data set in which they were originally generated to the data set of Hummel et al. (2006) as previously described [3].

3. Generation of correlated gene sets (CGSs)

To identify sets of correlated genes we used a similar procedure as in [4]. Each gene $i_1 = 1, \ldots, p$ was considered as a "central" gene of a gene set. The gene set included a gene i if all of the following conditions were satisfied:

1. The total sum of squares of gene i was not greater than that of the "central" gene i_1, i.e., $(\mathbf{x}_i - \bar{\mathbf{x}}_i)'(\mathbf{x}_i - \bar{\mathbf{x}}_i) \le (\mathbf{x}_{i_1} - \bar{\mathbf{x}}_{i_1})'(\mathbf{x}_{i_1} - \bar{\mathbf{x}}_{i_1})$ where $\mathbf{x}_{i_1}$ and $\mathbf{x}_i$ are vectors of expression values of the central gene i_1 and the gene i, respectively.
2. The correlation of genes i_1 and i was positive and its square was at least equal to a given constant c.
3. Gene i was among the 30 genes which were most strongly positively correlated with gene i_1.

We used Spearman's rank correlation $r_{S;i_1,i}$ and $c = 0.5$. The value of 0.5 is the lowest possible value which ensures that any two genes in a set created in such a way have a non-negative correlation. Similar value for the correlation threshold has beeen recommended by other authors [5]. Next, gene sets which consisted only of the central gene were eliminated. To extract non-overlapping gene sets, all gene sets were first sorted into descending order by

$$O_m = (\mathbf{x}_{i_1} - \bar{\mathbf{x}}_{i_1})'(\mathbf{x}_{i_1} - \bar{\mathbf{x}}_{i_1}) \sum_{i \in m} r_{S;i_1 i} \ ,$$

where *m* is the set of indices of the genes in the gene set. Thus, a gene set had a high position in the ranking if its central gene showed high variability, if other genes from the set were highly correlated with the central gene and if the gene set contained a large number of genes. Next, we went from the top to the bottom of the list of the ranked gene sets each time selecting a gene set only if it did not overlap with any preceding, already accepted gene set from the list. Finally, from the remaining, non-overlapping gene sets, top 50 gene sets were selected for further analysis.

Our motivation for limiting the maximal size of a gene set to 30 was:

1. It made more probable that gene sets which represented correlated but distinct biological processes would remain in the analysis after the selection of non-overlapping gene sets. Without the limit on the size of a gene set such gene sets would likely overlap with each other and only one of them would remain in the analysis.
2. The presence of different tumor entities in our data set could generate correlations among functionally unrelated genes. For example, a large number of genes were correlated to some extent because of their differential expression between Burkitt lymphomas and the DLBCLs in the BL/DLBCL data set of Hummel et al. (2006). Setting the maximum size of a gene set to 30 meant that the effective correlation threshold for creating gene sets among such genes was higher than c. Thus, the modification made the method of creating gene sets more adaptive to the level of background correlation among the neighboring genes.

3. One consequence of introducing a limit on the size of a gene set was that several smaller gene sets were created instead of a large one. This fact had an impact on the unsupervised analyses of the samples giving more importance to biological processes which involved a large number of genes since they were represented by several and not only one gene set.

The 50 CGSs created with the above procedure in both BL/DLBCL data sets [1,2] contained overall only a small fraction of the assayed genes – 6.8% (649 from 9580) and 8.9% (219 from 2460), respectively. To examine the overall overlap between the gene sets generated in the two data sets, we considered only unique genes (mapped by gene symbol) which were measured on both platforms and belonged to the gene sets. There were 141 such genes in the data set of Hummel et al. (2006) and 168 such genes in the data set of Dave et al. (2006). From these, 65 overlapped which means that on average, about 40% of the genes from one list were also present in the other.

4. Summaries of the CGSs

For summarizing expression of a gene set we used the formulas developed in [6]. Specifically, given an $n \times p$ data matrix $\boldsymbol{X}$ with columns representing genes and rows representing samples, we define an "individual coordinate" for patient j and gene i by

$$k_{ji} = \sqrt{\frac{n}{n-1}} \frac{x_{ji} - \bar{x}_i}{\sqrt{(\boldsymbol{x}_i - \bar{\boldsymbol{x}}_i)'(\boldsymbol{x}_i - \bar{\boldsymbol{x}}_i)}}$$

where $\boldsymbol{x}_i$ is a vector of expression values of gene i. "Individual coordinate" is the individual contribution of patient j to variable i (the relative expression of gene i in patient j). Individual coordinates of the members of gene set m can be summarized with the "individual set coordinate" defined by

$$k_{jm} = \frac{\sum_{i \in m} k_{ji}}{\sqrt{\sum_{h \in m} \sum_{i \in m} r_{hi}}}$$

where r_{hi} in the denominator is the correlation between genes h and i, given by

$$r_{hi} = \frac{(\boldsymbol{x}_h - \bar{\boldsymbol{x}}_h)'(\boldsymbol{x}_i - \bar{\boldsymbol{x}}_i)}{\sqrt{(\boldsymbol{x}_h - \bar{\boldsymbol{x}}_h)'(\boldsymbol{x}_h - \bar{\boldsymbol{x}}_h) \cdot (\boldsymbol{x}_i - \bar{\boldsymbol{x}}_i)'(\boldsymbol{x}_i - \bar{\boldsymbol{x}}_i)}} .$$

Thus, the expression of gene set m in the n samples is characterized by the vector $\boldsymbol{k}_m = (k_{1m}\ k_{2m} \ldots k_{nm})'$, and the matrix of the set coordinates of all gene sets is given by

$$\boldsymbol{K} = (\boldsymbol{k}_{m_1} \quad \boldsymbol{k}_{m_2} \quad \boldsymbol{k}_{m_3} \quad \ldots).$$

For simplicity, we do not use the term "set coordinate" in the main text of this study. Instead, we use the term CGS (correlated gene set) to denote a set of correlated genes and also its summary, i.e., its set coordinate.

5. Unsupervised analysis using principal components of the CGSs

For sorting arrays and CGSs in the heat maps we used a previously published method [6,7]. We explain it here using the singular value decomposition $\boldsymbol{K} = \boldsymbol{V}\boldsymbol{\Lambda}^{1/2}\boldsymbol{D}'$ and denoting the ith eigenvalue and the corresponding eigenvectors by λ_i, $\boldsymbol{v}_i$ and $\boldsymbol{d}_i$, respectively. In a heat map, it is desirable that the arrays (the rows of matrix $\boldsymbol{K}$) which show similar expression with respect to their CGSs are arranged close to each other. Moreover, their arrangement should

not be sensitive to noise. One option to achieve it would be to sort the vectors representing the individual arrays (rows of matrix $\boldsymbol{K}$) according to the eigenvector $\boldsymbol{v}_1$. Another way is to include the first and the second principal component in the sorting algorithm.

In the practical implementation of this method, we standardized the n row vectors of $\boldsymbol{V}_{(2)} = (\boldsymbol{v}_1 \quad \boldsymbol{v}_2)$ to unit length so that they all lied on a unit circle. The order of the vectors on the circle determined the order of the corresponding arrays in the heat map. The leftmost and the rightmost arrays in the heat map were chosen such that the angle between the corresponding row vectors was maximal. For sorting the CGSs we used the row vectors of $\boldsymbol{D}_{(2)} = (\boldsymbol{d}_1 \quad \boldsymbol{d}_2) = \boldsymbol{K}'\left(\boldsymbol{v}_1\lambda_1^{-1/2} \quad \boldsymbol{v}_2\lambda_2^{-1/2}\right)$ in a similar way. In the simplified version of the procedure, arrays and the CGSs were ordered by the values of $\boldsymbol{v}_i$ and $\boldsymbol{d}_i$, respectively, if the i th principal component was used.

We modified the procedure sligthly for sorting arrays in one data set based on the vectors of principal component loadings computed in the other data set. Let $\boldsymbol{K}_A$ denote a matrix of set coordinates of gene sets generated in data set A, and $\boldsymbol{K}_B$ a matrix of set coordinates of gene sets generated in data set A and mapped to data set B. Moreover, let $\boldsymbol{K}_A = \boldsymbol{V}_A \boldsymbol{\Lambda}_A^{1/2} \boldsymbol{D}_A'$ and $\boldsymbol{K}_B = \boldsymbol{V}_B \boldsymbol{\Lambda}_B^{1/2} \boldsymbol{D}_B'$ be their singular value decompositions. For sorting arrays and gene sets in data set B we used the row vectors of $\boldsymbol{K}_B \boldsymbol{D}_{A(2)}$ and $\mathbf{D}_{A(2)}$, respectively. If a gene set could not be mapped from one data set to the other then it was omitted from the computation.

6. Validation of the CGSA method on two published BL/DLBCL data sets

To validate the CGSA method, we examined whether the dimension reduction from several thousands of genes to only 50 CGSs led to loss of information related to the previously characterized lymphoma subtypes. We assessed this by looking at how well the CGSs could reproduce known molecular tumor classifications in an unsupervised manner. We began by identifying 50 CGSs in each of the two analyzed data sets. Next, we summarized expression of the genes in every CGS. Finally, we ordered the samples with respect to the values of their 50 CGSs.

Figure 1 shows that the method of sorting the samples by their 1st principal component (PC1) reproduced the distinction into Burkitt lymphoma and other lymphoma types independently in the BL/DLBCL data set of Hummel et al. (2006) and in the BL/DLBCL data set of Dave et al. (2006). Moreover, Hummel et al. (2006) provided an index which quantifies similarity of a sample to a typical Burkitt lymphoma sample (mBL-index) with respect to gene expression. They used this index to split their cohort into molecular Burkitt lymphomas (mBL), intermediate cases and non-mBLs. Interestingly, this index shows a strong correlation (r = 0.82 P = 2.85e-54) with our PC1. This close agreement with the results of Hummel et al. (2006) and Dave et al. (2006) confirms the validity of the CGSA method. Notably, the discovery of cases of Burkitt lymphoma using gene expression was the main objective of the two studies. For this purpose, the authors used other types of measurements in addition to gene expression while our approach was fully unsupervised. Figures 1A,B also show that one of the recently found groups of B-cell lymphomas termed “pathway activation patterns” [3] (PAP), PAP-1 and Burkitt lymphomas lie at the opposite ends of the spectrum of the cases. This is in accordance with the fact that Bentink et al. observed inverse expression pattern in the PAP-1 group as compared to the Burkitt lymphoma cases. Figure 1A also demonstrates the apparent similarity between the “host response” category [8] and PAP-1.

Potentially more information about the structures present in the data can be gained by using the first two principal components instead of only the first principal component for ordering the samples and the CGSs. Figure S1A shows such an arrangement of the BL/DLBCL data set of Hummel et al. (2006). In addition to Burkitt lymphomas, tumors from several other PAPs are co-localized in Figure S1A. Whereas data from in vitro experiments were used to identify “pathway activation patterns”, our approach did not require any external data. When

we applied the same algorithm to the BL/DLBCL data set of Dave et al. (2006), a clear division in the activated B-cell-like diffuse large B-cell lymphomas (ABC), the germinal center B-cell-like diffuse large B-cell lymphomas (GCB) and Burkitt lymphoma emerged (Figure S1D). This is remarkable, since the original discovery of the ABC and GCB subtypes required biological expertise in the selection of the genes to be used for clustering samples. Further exploration of the BL/DLBCL data set of Hummel et al. (2006) revealed that in this data set the 5th principal component (PC5) discriminates between the ABC and GCB lymphomas. Using the PC1 and the PC5 to order the samples of this data set exhibited a pattern of ABC, GCB and Burkitt lymphomas (Figure S2A). Taken together, these results indicate that the 50 CGSs generated with our procedure retained information about the main molecular features of the analyzed data sets.

a) Testing whether the unsupervised ordering of samples in the heatmaps reproduces known molecular subtypes of B-cell lymphomas

To test whether the unsupervised ordering of samples by the principal components (PCs) of the CGSs (Figure 1, Figures S1 and S2) was non-random with respect to the known molecular subtypes of mature aggressive B-cell lymphomas we applied the strategy described in Section “Testing for association of the CGSs with other biologic features of the patients” to principal components of the CGSs (instead of the CGSs themselves). We tested all 50 PCs. Additionally, we computed (but did not use for testing) the areas under the receiver operating characteristic curves (AUC) and estimated their 95% confidence intervals by bootstrapping samples (1000 iterations). In case of multiple classes, the stated AUC is an average of all pairwise AUCs [9].

In the BL/DLBCL data sets of Hummel et al. (2006), the PC1 which was used to generate Figure 1A was significantly associated with the classification into mBL and other B-cell lymphomas (R^2 = 0.59, AUC = 0.99 [0.985-0.998]), PC1 and PC2 (Figure S1A) with the PAPs (R^2 = 0.72, 0.36, AUC = 0.82, 0.79) and with the consensus clusters (R^2 = 0.36, 0.19, AUC = 0.77, 0.73). PC5 (Figure S2) was associated with the ABC and the GCB subgroups (R^2 = 0.58, AUC = 0.95 [0.92-0.98], mBLs and cases unclassified with respect to ABC/GCB were excluded). All adjusted P = 0.001.

In the BL/DLBCL data set of Dave et al. (2006), the PC1 (Figure 1B) was significantly associated with the molecularly defined BL and DLBCL groups (R^2 = 0.53, AUC = 0.98 [0.96-0.99]) and PC1 and PC2 (Figure S1D) with the PAPs (R^2 = 0.75, 0.08 (adjusted P = 0.019), AUC = 0.83, 0.66). PC2 was associated with the ABC and GCB subgroups (R^2 = 0.62, AUC = 0.96 [0.92-0.98], subgroups other than ABC and GCB were excluded from the test). All adjusted P = 0.001, unless otherwise stated.

b) Rubustness of the results of the unsupervised analysis with respect to the number of the CGSs

To examine robustness of the unsupervised analysis with respect to the number of the CGSs, we sorted the samples by the PC1 and PC2 in both analyzed data sets using not the top 50 but only the top 40, 30 and 20 CGSs ranked by O_m (Text S1). Figure S3 shows that the results remain very similar even for 30 CGSs.

c) Rubustness of the results of the unsupervised analysis to the sampling error within one data set

To determine how the ordering of samples depends on the sampling error, we split the BL/DLBCL data set of Hummel et al. (2006) into two parts. We used the division in the training set (113 samples) and the test set (107 samples) as given by the authors. Next, we applied the procedure of creating 50 CGSs to the two parts separately. We observed that the arrangement of the samples in one part of the data set was virtually independent of whether the CGSs used to order the samples were generated in the same or in the other part of the data set (Spearman’s rank correlation equal to 0.94 and 0.99 (P < 0.00001), respectively). The details are given in the following two paragraphs.

To determine how the unsupervised ordering of samples in the BL/DLBCL data set of Hummel et al. (2006) depended on the sampling error, we split this data set in two parts. We used the division in the training set (113 samples) and the test set (107 samples) as given in the original publication [1]. Next, we generated 50 CGSs independently in the two parts of the data set using the same method as the one applied to the complete data set. We used the 50 CGSs created in the training set to order the samples in the training set and (separately) in the test set. Similarly, we ordered the samples in the training set and also in the test set using the 50 CGSs which originated from the test set. Thus, we obtained two orderings of the training set and two orderings of the test set. As described in the next paragraph, we found that the orderings were highly correlated, with Spearman's rank correlation equal to 0.94 and 0.99 ($P < 0.00001$) for the training set and for the test set, respectively.

A correlation of two orderings of samples was computed as follows. The method which we used to order samples with respect to the expression of the 50 CGSs [6] treats a sample as a vector in a space with 50 dimensions. These vectors are projected on a two dimensional plane using the first two principal components. Then, the samples can be ordered according to the direction of their vectors on the plane. The obtained ordering is described by a permutation of the sample names. Which sample name comes first in the permutation vector and whether the sample names in the vector are arranged clockwise or anti-clockwise is arbitrary, due to the circular nature of the ordering. As a correlation between two orderings of samples, we reported the absolute value of the maximum Spearman's rank correlation of the corresponding permutation vectors. The maximization was over all samples being taken as the first elements of the permutation vectors. To determine the significance of the reported correlation, we repeated the computation with 100,000 simulated random pairs of orderings of the 113 samples. The maximum absolute value of correlation obtained from this simulation of the null distribution of correlations was equal to 0.46, suggesting that the observed correlations of 0.94 and 0.99 had p-values far below 0.00001.

d) Reproducibility of the results of the unsupervised analysis across data sets

Next, we asked whether given an ordering of samples in one data set, we could obtain a similar ordering in the other data set. That would be desirable, if, say, we identified a biologically or clinically relevant distinction in one data set and wished to classify patients from another data set according to that distinction. We mapped the CGSs created in the BL/DLBCL data set of Hummel et al. (2006) to the data set of Dave et al. (2006) by gene symbols and ordered the latter using the principal component loadings of the first two PCs computed in the former (Text S1, Section "unsupervised analysis using principal components of the CGSs"). The result is depicted in Figure S1B. Patients labeled as Burkitt lymphomas or PAP-1 still cluster as they did in the data set of Hummel et al. (2006). The size of the other PAP-s is small and their ordering should be interpreted with caution. The same procedure applied in the reverse direction, i.e., from the BL/DLBCL data set of Dave et al. (2006) to the data set of Hummel et al. (2006) reproduced the ordering into ABC, GCB and Burkitt lymphomas with striking accuracy (Figure S1C). Furthermore, we also obtained consistent results using the PC-loadings of the PC1 and PC5 from the data set of Hummel et al. (2006) to sort the samples of the data set of Dave et al. (2006) (Figure S2B). These analyses show that the orderings of samples generated with the CGSs are robust not only to sampling variability but also to the differences between microarray platforms.

7. Testing for the association between the CGSs and other biologic features of the patients

The matrix $\boldsymbol{X}$ is assumed to be the $n \times p$ data matrix with columns representing genes (variables) and rows representing patients (samples). The matrix $\boldsymbol{K}$ of set coordinates of the gene sets can be written as

$$\boldsymbol{K} = \begin{pmatrix} \boldsymbol{k}_{m_1} & \boldsymbol{k}_{m_2} & \dots \end{pmatrix} = (\boldsymbol{X} - \overline{\boldsymbol{X}})\begin{pmatrix} \boldsymbol{e}_{m_1} & \boldsymbol{e}_{m_2} & \dots \end{pmatrix} = (\boldsymbol{X} - \overline{\boldsymbol{X}})\boldsymbol{E} \,,$$

where the matrix of weights $\boldsymbol{E}$ is a function of $(\boldsymbol{X}-\overline{\boldsymbol{X}})'(\boldsymbol{X}-\overline{\boldsymbol{X}})$. Our procedure is based on the general method by Läuter [4]. The method takes into account that matrix $\boldsymbol{E}$ is random, i.e, that randomly selected genes are used. Let F_h denote a statistic for testing association between the set coordinate $\boldsymbol{k}_{m_h}$ and a different feature of the same set of patients. For example, if this feature is the presence or absence of a specific genomic aberration, then F_h can be the two-sample Beta-statistic.

Our null hypothesis for a single set coordinate $\boldsymbol{k}_{m_h}$ (with a fixed h) is that its all $n!$ permutations $\boldsymbol{k}^*_{m_h}$ are equally likely. In other words, we assume that under the null hypothesis all patients are exchangeable. Consequently, we can use the permutation test to examine this hypothesis. If we wish to test for the association between $\boldsymbol{k}_{m_h}$ and several other features, we can use the procedure of Westfall and Young [10] to adjust for multiple testing. Specifically, let $F_h^{(1)}, F_h^{(2)}, F_h^{(3)}, \ldots$ be the statistics corresponding to each of the features to be tested. From the null hypothesis that all $n!$ permutations $\boldsymbol{k}^*_{m_h}$ are equally likely follows that the corresponding $n!$ vectors $\left(F_h^{(1)*} \quad F_h^{(2)*} \quad \ldots\right)$ of statistics and also their maxima $F_h^* = \max_j F_h^{(j)*}$ are equally likely. This property allows for the method of Westfall and Young to be used. An observed statistic $F_h^{(j)}$ leads to significance on the level α if the number of permutations with $F_h^{(j)} \le F_h^*$ is not higher than $n!\alpha$. The corresponding p-value is given by $P_h^{(j)} = \frac{\#(F_h^{(j)} \le F_h^*)}{n!}$, and the null hypothesis is rejected if $P_h^{(j)} \le \alpha$. If the number $n!$ of all permutations $\boldsymbol{k}^*_{m_h}$ is large, one should use a random subset consisting of *r* permutations. It is important, however, always to include the original, unpermuted $\boldsymbol{k}_{m_h}$ in this subset.

If several set coordinates are tested, the p-values obtained separately for each set coordinate from the above procedure can be adjusted across the set coordinates with the Bonferroni correction. In this second step of adjustment, we do not apply the Westfall-Young principle, because the necessary multivariate exchangeability conditions are not fulfilled in all cases.

In the tests for association between the set coordinates and the recurrent genomic abnormalities we used two-sample Beta-statistics. The two groups compared were the group with and without a specific abnormality. For testing association with features other than genomic aberrations, we applied Beta-test for correlation, a test for two or more groups of samples, depending on the type of the variable tested. Then, we used the observed p-values as the statistics $F_h^{(j)}$ in the procedure of Westfall and Young, i.e., we used a min*P*-adjustment instead of a max*T*-adjustment [10]. Accordingly, we substituted “min” for “max” and $\ge$ for $\le$ at the appropriate places in the procedure described above. Since we tested 50 set coordinates, we set the number of permutations to $r = 50000$ such that the minimal attainable adjusted p-value was 0.001.

8. Validation of the CAPs in an independent data set

We obtained raw expression data from an independent data set of 414 DLBCL samples hybridized to the HG-U133plus2 GeneChip platform [11]. We normalized and summarized the data using a similar procedure to that applied to our data from the MMML-project. Next, we mapped the CGSs generated in our extended DLBCL data set to the data set of Lenz et al. (2008) [11] using Affymetrix IDs. We observed differences in expression between the 181 CHOP-treated and the 233 Rituximab-CHOP-treated samples of Lenz et al. (2008) [11]. Therefore, we standardized the CGSs separately in these two data sets to remove this batch

effect. Finally, we clustered all 414 samples of Lenz et al. (2008) [11] with respect to the 50 CGSs using the same algorithm as the one used to obtain the 3 CAPs (Partitioning around Medoids with Euclidean distances) and setting the number of clusters to 3. The result of the clustering is shown in Figure 2B. To examine whether the obtained clusters corresponded to our CAPs, we found centroids of each of the clusters generated in the data set of Lenz et al. (2008) [11] and in our extended DLBCL data set by computing the mean of each CGS over all samples from the appropriate subtype. Next, we computed correlations between the centroids from these two data sets (Figure 2C). A related method for presenting similarities between gene expression patterns found in expression subtypes across data sets was introduced previously [12].

9. Analysis of differential expression

Analysis of differential expression was performed with the R-package limma (linear models for microarray data analysis) [13]. The p-values were adjusted to control the false discovery rate (FDR) at 0.05 using the method of Benjamini-Hochberg [14]. Since our gene expression data were normalized with the VSN method [15], we used generalized log-ratios as schrinkage estimators of the log fold changes [15]. The generalized log-ratios were computed as differences between the normalized intensity values. Since the generalized log-ratios are approximately on the natural log scale, they had to be exponentiated to obtain the estimated fold changes. Before computing differerentially expressed genes, we removed probe sets without Entrez IDs and, in case of multiple probe sets per Entrez ID, we kept the probe set with the highest interquartile range in the analysis. This procedure resulted in 12679 probe sets. From these, 10910 could be mapped to either an LE gene (3585) or an HE gene (7325).

10. LE and HE genes

We obtained a list of gene symbols of the LE and HE genes from the authors of the original publication [16]. We mapped them to Entrez IDs and then to Affymetrix IDs using Bioconductor [17]. From the 22283 probesets, 5248 could be mapped to an LE gene and 12590 to an HE gene.

11. Kernel density estimation

Kernel density estimates of the gene expression distributions were computed using the function “density()” from the statistical software package R [18]. Default settings of this function were used.

12. Pathway analysis with PAGE (Pathway Analysis of Gene Expression)

The gene universe for this analysis was given by all Entrez IDs of the HE genes (n = 7325). There were 3394 HE genes which were not overexpressed in any of the CAPs and, therefore, do not appear in the Venn diagram (Figure 6A). Overlaps between the lists of differentially expressed genes and the Gene Ontology (GO) terms were evaluated using the PAGE method [19]. We used Biological Process annotations of the Gene Ontology and the default significance threshold of $P<0.005$. The resulting (log10-transformed) p-values are shown as heatmaps (negative log10 p-values for overrepresentation and log10 p-values for underrepresentation). Redundantly informative GO terms [19] were removed in the version of the computiation presented in the main text (Figure 6B). Figure S9 shows the result of the computation without removing the redundant GO terms.

13. Discussion of the CGSA method and its relation to other approaches

From the methodological perspective, there are some important distinctions between our approach and the previous related work in the field of gene expression analysis. The guiding principle of the method presented here is that every aspect of the construction and filtering of the CGSs depends solely on permutation-invariant statistics such as the overall variance and covariance of the genes. As a consequence, the tests for association of the CGSs with other

characteristics of the patients control the type I error rate [4]. In other words, data-dependent summaries of genes can be constructed and tested in the same data set. This is different from the majority of the literature on gene sets [20,21] which assumes that the gene sets to be tested in a data set were created independently from it. Another feature of our method is the focus on relatively few coordinately expressed sets of genes with large variance across samples [22,23]. We reasoned that genes not belonging to such sets are more likely to represent noise and, therefore, are better excluded. This view is supported by recent studies which indicate that genes which are relevant in cancer tend to form well-connected sub-networks of interactions [24]. In contrast to our approach, most clustering techniques as, e.g., the popular method of hierarchical clustering [25], make use of all genes. Furthermore, our gene sets have a structure in which there is one central gene with which all other members of the gene set correlate. This construction can be justified by the observation that important genes tend to form highly connected hubs in coexpression- and other regulatory networks [5,26,27,28,29,30]. Moreover, this one-factor structure of the gene sets has advantages from the statistical point of view. Conditional on the central gene of a gene set, all other genes in this set tend to be partially uncorrelated. This adds to the statistical stability of a summary of the gene set [31].
Another general approach which is alternative to clustering is to decompose the data in a series of factors, mostly by principal component analysis (PCA) [7,32,33] but also using more complicated methods [22,34,35,36,37]. The CGSA relaxes the assumption made by the PCA about the orthogonality of the factors but remains computationally simple. The created factors (CGSs) adapt more closely and in a more flexible way to the correlation structure of the genes. Thus, correlated but biologically distinct phenomena can be potentially represented by separate factors. The interpretability of the results is enhanced by the fact that each factor is associated with a set of genes.
The view that sets of coexpressed genes may be of special interest in gene expression analysis has been present in the literature for some time [5,25,38,39,40]. Moreover, some authors expressed the idea that the dimension of gene expression data can be reduced to a relatively small number of factors [7,33,41]. There is also a multitude of methods for testing differential expression on the level of gene sets [20,21]. An advantage of the strategy presented here is that it combines these ideas and approaches which have been up to now considered mostly in separation. As we have shown, the effective dimension reduction achieved by our method simplifies the study of relations between transcriptional, genomic and phenotypic features. Moreover, our recent research suggests that interpretations of the observed gene expression patterns in terms of pathway activities can be gained by mapping the CGSs to interventional cell line data [42].

14. References

1. Hummel M, Bentink S, Berger H, Klapper W, Wessendorf S, et al. (2006) A biologic definition of Burkitt's lymphoma from transcriptional and genomic profiling. N Engl J Med 354: 2419-2430.
2. Dave SS, Fu K, Wright GW, Lam LT, Kluin P, et al. (2006) Molecular diagnosis of Burkitt's lymphoma. N Engl J Med 354: 2431-2442.
3. Bentink S, Wessendorf S, Schwaenen C, Rosolowski M, Klapper W, et al. (2008) Pathway activation patterns in diffuse large B-cell lymphomas. Leukemia 22: 1746-1754.
4. Läuter J, Rosolowski M, Glimm E (2012) Exact multivariate tests - a new effective principle of controlled model choice. arXiv:12022045v1 [statME].
5. Zhang B, Horvath S (2005) A general framework for weighted gene co-expression network analysis. Stat Appl Genet Mol Biol 4: Article17.
6. Läuter J, Horn F, Rosolowski M, Glimm E (2009) High-dimensional data analysis: selection of variables, data compression and graphics--application to gene expression. Biom J 51: 235-251.
7. Alter O, Brown PO, Botstein D (2000) Singular value decomposition for genome-wide expression data processing and modeling. Proc Natl Acad Sci U S A 97: 10101-10106.

8. Monti S, Savage KJ, Kutok JL, Feuerhake F, Kurtin P, et al. (2005) Molecular profiling of diffuse large B-cell lymphoma identifies robust subtypes including one characterized by host inflammatory response. Blood 105: 1851-1861.
9. Hand DJ, Till RJ (2001) A Simple Generalisation of the Area Under the ROC Curve for Multiple Class Classification Problems. Machine Learning 45: 171-186.
10. Westfall PH, Young SS (1993) Resampling-based Multiple Testing. New York: John Wiley & Sons.
11. Lenz G, Wright G, Dave SS, Xiao W, Powell J, et al. (2008) Stromal gene signatures in large-B-cell lymphomas. N Engl J Med 359: 2313-2323.
12. Wilkerson MD, Yin X, Hoadley KA, Liu Y, Hayward MC, et al. (2010) Lung squamous cell carcinoma mRNA expression subtypes are reproducible, clinically important, and correspond to normal cell types. Clin Cancer Res 16: 4864-4875.
13. Smyth GK (2004) Linear models and empirical bayes methods for assessing differential expression in microarray experiments. Stat Appl Genet Mol Biol 3: Article3.
14. Benjamini Y, Hochberg Y (1995) Controlling the false discovery rate: A practical and powerful approach to multiple testing. J Roy Statist Soc Ser B 57: 289-300.
15. Huber W, von Heydebreck A, Sultmann H, Poustka A, Vingron M (2002) Variance stabilization applied to microarray data calibration and to the quantification of differential expression. Bioinformatics 18 Suppl 1: S96-104.
16. Hebenstreit D, Fang M, Gu M, Charoensawan V, van Oudenaarden A, et al. (2011) RNA sequencing reveals two major classes of gene expression levels in metazoan cells. Mol Syst Biol 7: 497.
17. Gentleman RC, Carey VJ, Bates DM, and others (2004) Bioconductor: Open software development for computational biology and bioinformatics. Genome Biology 5: R80.
18. R Development Core Team (2008) R: A language and environment for statistical computing. Vienna, Austria: R Foundation for Statistical Computing.
19. Goodarzi H, Elemento O, Tavazoie S (2009) Revealing global regulatory perturbations across human cancers. Mol Cell 36: 900-911.
20. Ackermann M, Strimmer K (2009) A general modular framework for gene set enrichment analysis. BMC Bioinformatics 10: 47.
21. Nam D, Kim SY (2008) Gene-set approach for expression pattern analysis. Brief Bioinform 9: 189-197.
22. Hastie T, Tibshirani R, Eisen MB, Alizadeh A, Levy R, et al. (2000) 'Gene shaving' as a method for identifying distinct sets of genes with similar expression patterns. Genome Biol 1: RESEARCH0003.
23. Segal E, Friedman N, Koller D, Regev A (2004) A module map showing conditional activity of expression modules in cancer. Nat Genet 36: 1090-1098.
24. Ideker T, Sharan R (2008) Protein networks in disease. Genome Res 18: 644-652.
25. Eisen MB, Spellman PT, Brown PO, Botstein D (1998) Cluster analysis and display of genome-wide expression patterns. Proc Natl Acad Sci U S A 95: 14863-14868.
26. Barabasi AL, Albert R (1999) Emergence of scaling in random networks. Science 286: 509-512.
27. Han JD, Bertin N, Hao T, Goldberg DS, Berriz GF, et al. (2004) Evidence for dynamically organized modularity in the yeast protein-protein interaction network. Nature 430: 88-93.
28. Jeong H, Tombor B, Albert R, Oltvai ZN, Barabasi AL (2000) The large-scale organization of metabolic networks. Nature 407: 651-654.
29. Basso K, Margolin AA, Stolovitzky G, Klein U, Dalla-Favera R, et al. (2005) Reverse engineering of regulatory networks in human B cells. Nat Genet 37: 382-390.
30. Horvath S, Zhang B, Carlson M, Lu KV, Zhu S, et al. (2006) Analysis of oncogenic signaling networks in glioblastoma identifies ASPM as a molecular target. Proc Natl Acad Sci U S A 103: 17402-17407.
31. Dettling M, Bühlmann P (2004) Finding predictive gene groups from microarray data. Journal of Multivariate Analysis 90: 106-131.

32. Raychaudhuri S, Stuart JM, Altman RB (2000) Principal components analysis to summarize microarray experiments: application to sporulation time series. Pac Symp Biocomput: 455-466.
33. Fehrmann RS, de Jonge HJ, Ter Elst A, de Vries A, Crijns AG, et al. (2008) A new perspective on transcriptional system regulation (TSR): towards TSR profiling. PLoS One 3: e1656.
34. Hastie T, Tibshirani R, Botstein D, Brown P (2001) Supervised harvesting of expression trees. Genome Biol 2: RESEARCH0003.
35. Chang JT, Carvalho C, Mori S, Bild AH, Gatza ML, et al. (2009) A genomic strategy to elucidate modules of oncogenic pathway signaling networks. Mol Cell 34: 104-114.
36. Witten DM, Tibshirani R, Hastie T (2009) A penalized matrix decomposition, with applications to sparse principal components and canonical correlation analysis. Biostatistics 10: 515-534.
37. Yu T An exploratory data analysis method to reveal modular latent structures in high-throughput data. BMC Bioinformatics 11: 440.
38. Segal E, Shapira M, Regev A, Pe'er D, Botstein D, et al. (2003) Module networks: identifying regulatory modules and their condition-specific regulators from gene expression data. Nat Genet 34: 166-176.
39. Alizadeh AA, Eisen MB, Davis RE, Ma C, Lossos IS, et al. (2000) Distinct types of diffuse large B-cell lymphoma identified by gene expression profiling. Nature 403: 503-511.
40. Ihmels J, Friedlander G, Bergmann S, Sarig O, Ziv Y, et al. (2002) Revealing modular organization in the yeast transcriptional network. Nat Genet 31: 370-377.
41. Liu A, Zhang Y, Gehan E, Clarke R (2002) Block principal component analysis with application to gene microarray data classification. Stat Med 21: 3465-3474.
42. Sander S, Calado DP, Srinivasan L, Kochert K, Zhang B, et al. (2012) Synergy between PI3K Signaling and MYC in Burkitt Lymphomagenesis. Cancer Cell 22: 167-179.
